# Evaluating parameters for ligand-based modeling with random forest on sparse data sets

**DOI:** 10.1186/s13321-018-0304-9

**Published:** 2018-10-11

**Authors:** Alexander Kensert, Jonathan Alvarsson, Ulf Norinder, Ola Spjuth

**Affiliations:** 1 Department of Pharmaceutical Biosciences, Uppsala University, Uppsala, Sweden; 2 Unit of Toxicology Sciences, Karolinska Institutet, Swetox, Forskargatan 20, SE-15136 Södertälje, Sweden; 3 Department of Computer and Systems Sciences, Stockholm University, Box 7003, SE-164 07 Kista, Sweden

**Keywords:** Random forest, Support vector machines, Sparse representation, Fingerprint, Machine learning

## Abstract

Ligand-based predictive modeling is widely used to generate predictive models aiding decision making in e.g. drug discovery projects. With growing data sets and requirements on low modeling time comes the necessity to analyze data sets efficiently to support rapid and robust modeling. In this study we analyzed four data sets and studied the efficiency of machine learning methods on sparse data structures, utilizing Morgan fingerprints of different radii and hash sizes, and compared with molecular signatures descriptor of different height. We specifically evaluated the effect these parameters had on modeling time, predictive performance, and memory requirements using two implementations of random forest; Scikit-learn as well as FEST. We also compared with a support vector machine implementation. Our results showed that unhashed fingerprints yield significantly better accuracy than hashed fingerprints ($p \le 0.05$), with no pronounced deterioration in modeling time and memory usage. Furthermore, the fast execution and low memory usage of the FEST algorithm suggest that it is a good alternative for large, high dimensional sparse data. Both support vector machines and random forest performed equally well but results indicate that the support vector machine was better at using the extra information from larger values of the Morgan fingerprint’s radius.

**Electronic supplementary material:**

The online version of this article (10.1186/s13321-018-0304-9) contains supplementary material, which is available to authorized users.

## Background

Ligand-based modelling is a widely used method where the ligand’s activity for a biological target, usually a measurement obtained from a bioassay, can be correlated to certain features of the ligand. The derived model can then be used for predicting the biological activity of new novel chemical compounds. Examples of applications include studies on bio-availability [1], bioactivity of GPCR-associated ligands [2], mitochondrial toxicity [3], organ toxicity [4], hepatotoxicity [5] and aquatic toxicity [6].

In quantitative structure–activity relationships (QSAR), chemical structures are represented as numerical features via algorithms referred to as molecular descriptors. An important example is ECFP (Extended-Connectivity Fingerprints), which are molecular descriptors specifically developed for structure–activity modelling. The original article of ECFP illustrate the strengths of the algorithm and how it can be applied in a variety of computational chemistry domains [7]. In classification problems ECFP has, for example, been valuable for predicting inhibition of Cytochrome P450, 2D6 and 3A4 [8] and of *Escherichia coli* dihydrofolate reductase [9]. It has also been applied in quantitative structure–property relationships (QSPR) for studying melting points and aqueous solubility of organic compounds [10].

Machine learning (ML) algorithms is an important component in structure–activity modelling and analysis of compounds, and an example of a widely used method is support vector machines (SVMs). This method has proven to be successful for correlating molecular structures to toxicity and activity of compounds [3, 4, 11, 12]. SVMs have also shown to be useful for drug transport predictions [13] and to model and study interactions of antibiotic compounds [14]. Another important and successful machine learning method is the random forest (RF) [15], which like SVM has proven to be valuable in toxicity and bio-activity studies [2, 5, 6, 16, 17], and also for investigating diverse representations of clinical events [18] as well as predicting adverse drug events from electronic health records [19]. RF has been one of the most widely used ML algorithms together with SVM, and studies have demonstrated that RF is a powerful yet easy-to-implement method in both QSAR and descriptor selection [20, 21].

Although extensive and rigorous research have been performed in the area of QSAR and ML methods, there are few comprehensive studies on RF together with *Morgan fingerprints* [22], a powerful ECFP variant [7], so we set out to study random forest in general, and the random forest implementation FEST (Fast Ensembles of Sparse Trees) in particular, together with Morgan fingerprints.

The Python package Scikit-learn contains a well known and much used random forest implementation which we decided to use as a reference point. Since SVM is highly used in QSAR and since SVM together with molecular signatures has been shown to perform well for this kind of QSAR tasks [23, 24] we decided to include an SVM as well in the study as another reference point.

In this article the following thoughts and ideas are examined further: Perhaps working with hashed fingerprints of smaller sizes would speed up the modeling or make it require less memory without resulting in worse models? How many collisions would hashing to different fingerprint size give rise to in typical QSAR data sets? What effect does different values for the random forest parameter *Max features* have on the prediction models? What radii should be used for the Morgan fingerprints? Higher values would mean more data and it would seem reasonable to expect more data to give better models but at the cost of modeling time. Would there be a trade-off there? Random forests do not require the extended parameter search that support vector machines require so can they be trained faster for the QSAR problem?

## Methods

### Data

Four different public datasets were used in this study, containing 5000–7000 compounds each (Table 1). Three of them were obtained from the tox21 challenge (https://tripod.nih.gov/tox21/challenge/data.jsp), and contains data from QHTS assays to identify small molecules that: (1) activate the aryl hydrocarbon receptor (nr-ahr), (2) act as agonists of the estrogen receptor alpha signaling pathway using the BG1 cell line (nr-er), and (3) disrupt the mitochondrial membrane (sr-mmp) [25]. The fourth data set was obtained from the paper “Benchmark Data Set for in Silico Prediction of Ames Mutagenicity” by Hansen and co-workers [26].

**Table 1:** The data sets used in the study

Dataset	Negatives	Positives	Sum
sr-mmp	4763	884	5647
nr-ahr	5599	700	6299
nr-er	5235	623	5858
cas-N6512	3007	3502	6509

Structure standardization was performed using the IMI eTOX project standardizer (version 0.1.7. https://pypi.python.org/pypi/standardiser) in combination with the MolVS standardizer (version 0.0.9. https://pypi.python.org/pypi/MolVS) for tautomer standardization. The Python libraries Matplotlib (version 2.1.0) and Seaborn (version 0.8.0) were used to illustrate the results of this study [27, 28].

### Morgan fingerprints

There are numerous ways of generating molecular fingerprints. In this study, the open-source Python framework RDKit (version 2017.09.1) was used to generate Morgan fingerprints [29]. The atomic invariants of these fingerprints use connectivity information similar to the the Extended Connectivity Fingerprints (ECFP) family of fingerprints [7, 29]. The Morgan algorithm initially assigns an integer identifier to each non-hydrogen atom, then iteratively, by extending the connectivity of each atom to its neighbouring atoms, updates the numerical identifiers based on these neighbouring atoms [7]. There are mainly two parameters to be set for the generation of the Morgan fingerprint: (1) bit size (or hash size)—the length of the bit string for the molecular features to be contained in; (2) radius—the number of neighbours $\times$ bond lengths away to take into account when calculating the identifiers of the atoms. A fingerprint collision occur when a feature falls into a bin (a dataset column) of another feature—resulting in more than one molecular substructure being compressed into a single, now hashed, feature. It is also possible to use an unhashed version of the fingerprint, which means that the compression of the bit string is bypassed and hence encode explicitly defined patterns. In this study, both the unhashed and hashed Morgan fingerprints were generated and compared (Table 2).

**Table 2:** Overview of the molecular descriptors used in the study

Morgan fingerprints		Molecular signatures
Hash size	Radius	Height
128	1	1–1
256	2	1–2
512	3	1–3
1024		
2048		
4096		
Unhashed		

### Molecular signatures

In addition to the Morgan fingerprints, molecular signatures [30] were generated and evaluated. The molecular signatures is a molecular descriptor similar to the Morgan fingerprints in the sense that its identifiers are based on the neighbouring of atoms. Contrary to the Morgan fingerprint, the signature descriptors do not hash the information into an index, but generate explicitly defined substructures, which are then mapped to numerical identifiers. Equivalent to the radius of Morgan fingerprints, signatures have a height parameter which extends away one or several bonds from the investigated atom (Table 2).

### Random forests

Random forests are ensembles of decision trees [31]. According to the strong law of large numbers, growing a large number of decision trees lead to better generalization and prevention of over-fitting. This generalization error converges as the number of trees grow and depend on the strength of and correlation between the individual trees [15, 32]. Importantly, the correlation between the individual trees is reduced by several random processes in the random forest algorithm. First, for the *S* number of trees in the forest, *S* number of new datasets are randomly sampled (with replacement). Second, a random subspace method is used which selects a subset of *m* features from the total number of features *M* before each split—normally determined by the information gain or Gini impurity metric [33]. The splitting, or branching, continues until it reaches a leaf node, which contains a class label probability. Hence the ensemble of trees produces *S* outputs for an input *x*, each with its own probability for the classes. This is then averaged across the ensemble of trees to generate a final prediction of the class with the highest probability.

*Fast ensembles of sparse trees* (FEST) is a software written in C for learning various types of decision tree committees from high dimensional sparse data [34] that efficiently handles sparse data structures. The current implementation allows for setting different hyperparameter values for RF, such as (1) Max features (the maximum number of allowed splitting features to be considered), (2) number of trees, (3) maximum depth of the tree, and (4) relative weight for the negative class.

*Scikit-learn’s random forest classifier* (Scikit RF) is part of an open source machine learning framework in Python [35]. Scikit RF allows for experimenting with the same hyperparameters as the FEST implementation and many more. Like FEST, Scikit RF supports sparse matrix operations, but also parallelization, which can speed up model training when using multiple CPUs.

In this study the information gain metric was used for both RF implementations, and *Maximum Depth* parameter was set to large enough (1000) to not affect the tree depth. To accommodate for inbalanced datasets, the parameter *relative negative weights* (FEST) and *class weights* (Scikit RF) were set to balance the classes. *Max features* and *Number of trees* were two hyperparameters selected for investigations (Table 3).

**Table 3:** Overview of the different machine learning methods and parameter settings used in the study

FEST and Scikit RF		Scikit SVM	
Max features$^{\hbox {a}}$	Trees	*C*	$\gamma$
0.1	10	0.01	$1\times 10^{-6}$
0.3	30	0.1	$3\times 10^{-6}$
1.0	100	1	$1\times 10^{-5}$
3.0	300	10	$3\times 10^{-5}$
10.0	1000	100	$1\times 10^{-4}$
		1000	$3\times 10^{-4}$
		$1\times 10^{4}$	0.001
		$1\times 10^{5}$	0.003
		$1\times 10^{6}$	0.01
		$1\times 10^{7}$	0.03
		$1\times 10^{8}$	0.1

$^{\hbox {a}}$Values indicate the multiplicating factor by the square root of the number of features

### Support vector machines

*Scikit-learn’s C-support vector classifier* (Scikit SVM) [35] is based on the Libsvm implementation of support vector machines [36]. Like the FEST and Scikit RF implementations, Scikit SVM handles sparse data structures in an efficient way. Support vector machines use kernel functions to non-linearly map inseparable inputs *X* to a higher dimensional space $\phi ({\textit{X}})$ where they can be linearly separated by a hyper-plane. Scikit SVM handles high dimensional data efficiently by solving the dual problem, where instead of learning a weight vector *w* of possibly thousands of dimensions which require significant computational power, instead learns a vector $\alpha$ in the dual problem which contains all zeros except for the support vectors. In this study, the radial basis function (RBF) was used as kernel, and the hyperparameters investigated were *Cost (C)* and the kernel parameter *gamma (*$\gamma$*)* (Table 3).

### Model evaluation

In this investigation, ROC–AUC was used as metric for evaluating the different models. A receiver operating characteristic curve (ROC-curve) is a graphical plot illustrating the true positive rates (TPR) against the false positive rate (FPR) at different thresholds. The AUC is then the area under this curve, which has shown to be a valid and advantageous metric for evaluating ML algorithms [37]. For each hyperparameter combination, a 5-fold randomly shuffled cross-validation was utilized to yield a cross-validated ROC–AUC score; the procedure was repeated five times to produce a more robust metric with respect to the models performance. In the case of Scikit SVM, ROC–AUC scores were calculated with respect to the decision function.

## Results and discussion

We studied the effect of two random forest implementations (Scikit RF and FEST) and a C-support vector classifier (Scikit SVM) on sparse datasets for ligand-based modelling. Specifically evaluating combinations of parameters according to Tables 2 and 3, and their effect on ROC–AUC, memory usage and run-time.

All datasets were subjected to descriptor generation using Morgan fingerprints and molecular signatures (Tables 2 and 4). Hashed versions of the Morgan fingerprints were generated with 128, 256, 512, 1024, 2048, and 4096 bins.

**Table 4:** Number of of compounds and number of descriptors generated for the different data sets and molecular descriptors

	Data sets			
	sr-mmp	nr-ahr	nr-er	cas N6512
*Morgan fingerprints*				
#Compounds	6299	5647	5858	6509
Radius 1	3352	3525	3350	2935
Radius 2	21,542	23,695	21,974	19,131
Radius 3	49,764	55,725	51,200	48,325
*Molecular signatures*				
#Compounds$^{{\hbox {a}}}$	6193	5546	5761	6396
Height 1–1	504	514	487	405
Height 1–2	7524	8021	7547	6758
Height 1–3	33,237	36,601	33,900	31,581

$^{{\hbox {a}}}$Number of compounds for molecular signatures are lower because the algorithm couldn’t generate descriptors for some compounds

Every hyperparameter combination of each ML method was trained on the datasets with every descriptor parameter combination (Tables 1, 2 and 3). ROC–AUC, memory usage and run-time were measured for all runs.

Overall the tested machine learning algorithms and descriptors produce models of similar prediction capacity (Fig. 5) which is not surprising considering these are all commonly used methods in QSAR and should be expected to produce good results. There are however some differences that might be relevant depending on use cases.

### Effect of hashed versus non-hashed features

**Fig. 1:**
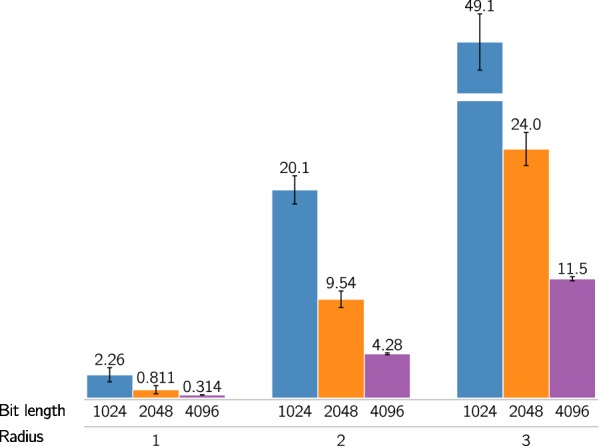
Collisions per feature. Number of collisions per feature for different hash sizes averaged over the four datasets using the Morgan fingerprint descriptor. Error bars are standard deviation of the mean

To illustrate both the way hashing reduces the dimensions of a data set, as well as decreases the “resolution” of the fingerprints, collisions for all data sets using the Morgan fingerprint were plotted (Fig. 1).

**Fig. 2:**
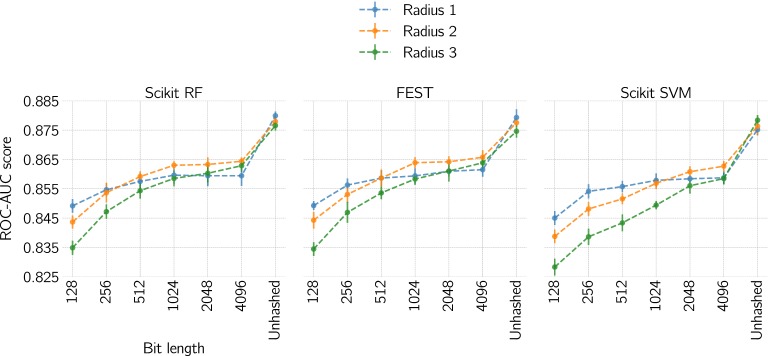
Effect of hash size and radius on predictive performance with Scikit RF, FEST and Scikit SVM. Each data point is an average of the best ROC-AUC score for each dataset. Error bars are pooled standard deviations

**Fig. 3:**
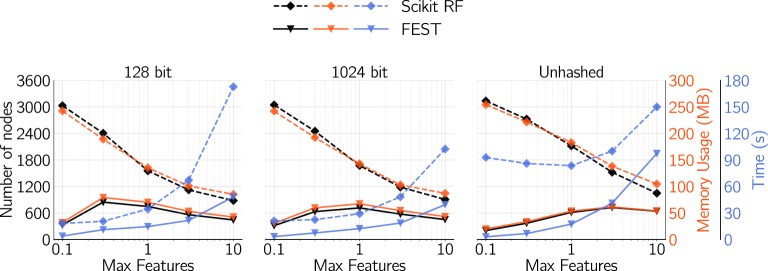
Effect of* Max features* and hash size on number of nodes (per tree), memory usage and run time for the two random forest implementations with 1000 trees. Data points are average values of the four datasets, and although imperceptible due to minuscule values: error bars are pooled standard deviations

**Fig. 4:**
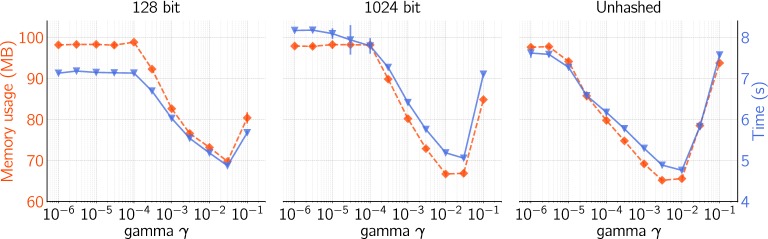
Effect of* gamma* ($\gamma$) and hash size on memory usage and run-time for Scikit SVM with* Cost* (*C*) equal to 1. Data points are average values of the four datasets, error bars are pooled standard deviations

Further investigation into hash sizes illustrated that there are no obvious differences between Scikit SVM, Scikit RF and FEST in terms of ROC–AUC scores, with a plateauing of performance beyond 1024 bit, and a spike from 4096 to unhashed (Fig. 2; “Appendix”). However, our results show that unhashed fingerprints yield better performance compared to hashed fingerprints according to the difference between the areas under the two ROC curves using the method of Hanley and McNeil [38] (for the full result table, see Additional file 1: Table S1). For all statistically significant cases ($p \le 0.05$) were the ROC curve area for unhashed fingerprints larger than for hashed fingerprints for each dataset, respectively. Also, for almost all of the non-significant cases (265 out of 268 cases) were the ROC curve area larger for unhashed fingerprints than for hashed fingerprints. Considering the significant improvements in predictive performance, with no pronounced difference in memory usage or run-time (Figs. 3, 4), the preferred choice should be to use unhashed fingerprints. This result is also in line with previously reported results on fingerprints in a virtual screening setting [39].

An advantage of using unhashed fingerprints is that the features have a particular substructure assigned to them and can therefore be traced back to the actual molecular features. Thus using unhashed fingerprints mean that each feature represents a certain molecular substructure, and by assessing feature importance, this can be helpful in interpreting model results in a chemical context [40–42].

An explanation of how memory usage and run-time for RF models trained on unhashed Morgan fingerprints are similar to hashed fingerprints could be that more information with unhashed fingerprints results in shorter trees, i.e. the splits are better. Concretely, hashed fingerprints are compressed and contain a lot of noise, which makes it harder for the trees to separate the class labels and reach leaf nodes.

### Effect of Max features for the two random forests

In the case of Scikit RF, a clear decrease in memory usage can be observed with increasing number of features. This is highly correlated with the number of nodes in the trees (Fig. 3) indicating that more features to select from at each node results in better splits and shorter trees. Interestingly, this pattern cannot be observed with FEST. FEST however has a lower memory consumption and faster training than Scikit RF for all tested values of the *Max features* setting. Figure 4 illustrates the fast training-time of Scikit SVM, as well as its memory usage. Although the Scikit SVM could outperform RF in terms of run-time for a single run, Scikit SVM was sensitive to hyperparameter settings and required extensive grid-searches (see “Hyperparameter space of Scikit SVM” section).

**Fig. 5:**
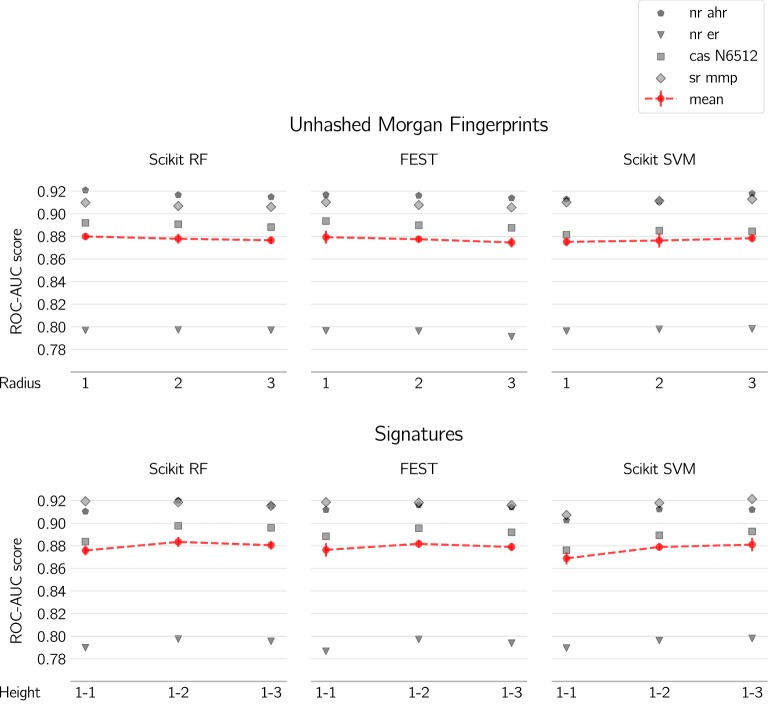
Effect of fingerprint radii/height on predictive performance with Scikit RF, FEST and Scikit SVM. Grey markers represent the four datasets. Red markers/dashed lines indicate the average of the four datasets, with error bars being pooled standard deviations

### Effect of fingerprint radii

We further investigated unhashed fingerprint radii including molecular signatures for comparison (Fig. 5). This is important in order to evaluate how increased sizes of substructures improve the separation of the two classes (i.e. improve predictive performance) by the ML methods.

For SVM it seems that more data does indeed results in a better model whereas for the random forest implementations no upward trend can be observed when increasing the radius (Fig. 5). One reason why no upward trend is seen with random forest could be that the number of features of radius/height 1 “drown” in the much larger number of features of radius/height 2 and 3. The random subspace methods randomly selects a subset of features *m* among the total number of features *M*, where *M* is dominated by radii/heights 2 and 3, which are more abundant than radii/heights of 1 and hence cannot reduce entropy to the same extent.

### Random forest and support vector machines

As stated before it seems that SVM and RF perform very equally well on our data sets but it is interesting to note that as more data is included by adding more heights to the molecular signatures, or larger radii to the Morgan fingerprints, the RF seems to decrease in performance where SVM seems to increase. Based on this it seems reasonable to theorize that with more data and extensive grid search for the SVM parameters it is possible that SVM could perform better than RF but at much larger computational costs. If and when it is worth it probably varies from project to project. Also, we did not see this in our case, our Scikit SVM models did not perform better than our random forest models.

### Hyperparameter space of Scikit SVM

**Fig. 6:**
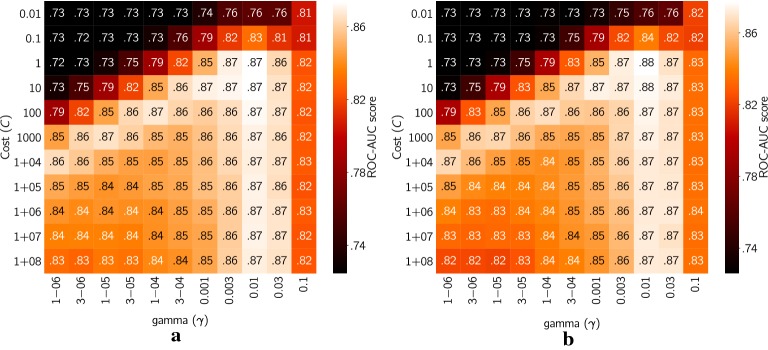
Heat plots illustrating ROC-AUC scores with different hyperparameter combinations of Scikit SVM.** a** modelled on unhashed Morgan fingerprints with radius 3;** b** modelled on molecular signatures with height 1-3. Both plots are averages of the four datasets

For the comparison between RF and Scikit SVM, an extensive grid search for the hyperparameters of Scikit SVM was needed. We evaluated *Cost (C)* and *gamma* ($\gamma$) according to Table 3, by projecting the ROC–AUC scores of different hyperparameter combinations to a heat map (Fig. 6).

These heat plots illustrate the delicacy with which SVM models in QSAR (Fig. 6) must be treated where just a small space of possible hyperparameter values gives scores that compare with (and sometimes exceed) models from the random forest implementations. However, these observations agree with previous results from a study by Alvarsson et al. [43], where heat plots of models trained on molecular signatures were made with seven different public QSAR datasets. This suggest that the “hot spots” are found at very similar hyperparameter combinations for Morgan fingerprints and molecular signatures meaning that the parameter values that needs to be tested in order to optimise the SVM models actually are feasible and even though it requires more computation than random forest it could be justified in some cases.

### Availability

All datasets and code for the analysis is available at: https://github.com/pharmbio/kensert_rf_sparse, with an archived release at Zenodo http://doi.org/10.5281/zenodo.1291787.

## Conclusions

We present evidence that hashing of Morgan fingerprints descriptors for QSAR modeling has a negative effect on predictive performance, with no significant improvement in computational efficiency. The FEST implementation was found to be capable of producing models of the same prediction quality as Scikit RF (SciKit), using less computational time and with lower memory requirements, however Scikit RF can be more easily parallelized on multi-core computers. The usefullness of this depends on the problem; building multiple smaller models is “embarrassingly parallelizable” and then the faster FEST implementation can be recommended but when building fewer large models than the built in parallelisation of Scikit RF will be relevant. For the Scikit implementation of random forest it was found that higher values for the *Max features* setting actually resulted in lower memory use but this could not be seen for FEST. Furthermore, no clear trend was identified that an increased number of features, i.e. increased radii/height, impacts favorably on the predictive performance for the random forest implementations. Evaluations of Scikit SVM and random forests demonstrate that both methods perform well but that SVM requires a more extensive grid search and tuning to reach high ROC–AUC scores but perhaps is better at taking advantage of the additional data found in higher radii of molecular signatures/Morgan fingerprints. Considering the easy and robust implementation of random forests this method could be considered a good initial choice for most cases and when the best results are needed SVM can be tested as well but at possibly a higher computational cost.

### Additional file

**Additional file 1: Table S1.** Computed *p* values according to the difference between the areas under two ROC curves using the method of Hanley and McNeil.

## Appendix

See Table 5.

**Table 5:** Effect of hash size on predictive performance with Scikit RF, Fest and Scikit SVM

Method	Radius	Hash size	Mean	SD
			ROC AUC	
Scikit RF	1	128	0.849175	0.0023297
Scikit RF	1	256	0.854675	0.00229456
Scikit RF	1	512	0.857525	0.00262345
Scikit RF	1	1024	0.85965	0.00294236
Scikit RF	1	2096	0.85945	0.00357561
Scikit RF	1	4096	0.85945	0.00348676
Scikit RF	1	Unhashed	0.879925	0.00144135
Scikit RF	2	128	0.843675	0.00222542
Scikit RF	2	256	0.853675	0.0033908
Scikit RF	2	512	0.859175	0.00176494
Scikit RF	2	1024	0.863025	0.0014151
Scikit RF	2	2096	0.8633	0.00241454
Scikit RF	2	4096	0.8644	0.00129808
Scikit RF	2	Unhashed	0.87795	0.00214126
Scikit RF	3	128	0.834875	0.00240936
Scikit RF	3	256	0.8472	0.00245917
Scikit RF	3	512	0.85435	0.00269165
Scikit RF	3	1024	0.858525	0.00269258
Scikit RF	3	2096	0.8603	0.0031249
Scikit RF	3	4096	0.862875	0.00200624
Scikit RF	3	Unhashed	0.876575	0.00171391
FEST	1	128	0.849275	0.00152151
FEST	1	256	0.856275	0.00226661
FEST	1	512	0.85865	0.00271616
FEST	1	1024	0.8594	0.00257633
FEST	1	2096	0.860975	0.00175784
FEST	1	4096	0.861525	0.00239008
FEST	1	Unhashed	0.879325	0.00281158
FEST	2	128	0.844275	0.00282975
FEST	2	256	0.8531	0.00292104
FEST	2	512	0.85865	0.00290086
FEST	2	1024	0.8639	0.00189143
FEST	2	2096	0.864225	0.00191703
FEST	2	4096	0.86575	0.00242178
FEST	2	Unhashed	0.87755	0.00140624
FEST	3	128	0.83445	0.00234254
FEST	3	256	0.84695	0.00355633
FEST	3	512	0.8536	0.00216102
FEST	3	1024	0.858325	0.00195704
FEST	3	2096	0.86105	0.00356686
FEST	3	4096	0.863875	0.00230326
FEST	3	Unhashed	0.874625	0.00217658
Scikit SVM	1	128	0.845025	0.00236326
Scikit SVM	1	256	0.854125	0.00246475
Scikit SVM	1	512	0.85575	0.00195512
Scikit SVM	1	1024	0.857875	0.0023516
Scikit SVM	1	2096	0.858425	0.00126293
Scikit SVM	1	4096	0.85875	0.00224666
Scikit SVM	1	Unhashed	0.875175	0.00193197
Scikit SVM	2	128	0.83875	0.00233024
Scikit SVM	2	256	0.848125	0.00232433
Scikit SVM	2	512	0.85155	0.00185876
Scikit SVM	2	1024	0.856875	0.00197927
Scikit SVM	2	2096	0.860825	0.00186615
Scikit SVM	2	4096	0.862725	0.00172699
Scikit SVM	2	Unhashed	0.87635	0.00306716
Scikit SVM	3	128	0.8283	0.00287359
Scikit SVM	3	256	0.838625	0.00278298
Scikit SVM	3	512	0.843375	0.00289093
Scikit SVM	3	1024	0.849425	0.00141067
Scikit SVM	3	2096	0.85605	0.00269629
Scikit SVM	3	4096	0.858475	0.00194936
Scikit SVM	3	Unhashed	0.8784	0.0017713

Each data point is a mean of the four data sets. These values are plotted as Fig. 2 in the publication
