# Supplementary material for: Evaluating parameters for ligand-based modeling with random forest on sparse data sets
Source: J Cheminform. 2018 Oct 11;10:49. doi: 10.1186/s13321-018-0304-9 (PMC6755600; doi:10.1186/s13321-018-0304-9)
Supplement: Supplementary file 1 — Additional file 1: Table S1. Computed p values according to the difference between the areas under two ROC curves using the method of Hanley and McNeil. [file 13321_2018_304_MOESM1_ESM.pdf]

Additional Table 1.
Computed p-values according to the difference between the areas under two ROC curves using the method of Hanley and McNeil

infile_a	auc_a	fptype_a	radius_a	infile_b	auc_b	fptype_b	radius_b	P: non-directional (two-tailed)	difference auc_b-auc_a	stat significant
cas-N6512_RFC	0.8787	512	2	cas-N6512_RFC	0.8908	UNHASHED	2	0.038965526	0.0121	y
cas-N6512_RFC	0.8798	512	1	cas-N6512_RFC	0.892	UNHASHED	1	0.036433456	0.0122	y
cas-N6512_FEST	0.8775	1024	3	cas-N6512_FEST	0.8899	UNHASHED	2	0.035187047	0.0124	y
cas-N6512_FEST	0.8811	4096	3	cas-N6512_FEST	0.8936	UNHASHED	1	0.03098692	0.0125	y
cas-N6512_FEST	0.875	256	2	cas-N6512_FEST	0.8876	UNHASHED	3	0.034131289	0.0126	y
cas-N6512_RFC	0.8776	256	1	cas-N6512_RFC	0.8908	UNHASHED	2	0.024644999	0.0132	y
cas-N6512_FEST	0.8804	512	2	cas-N6512_FEST	0.8936	UNHASHED	1	0.022933832	0.0132	y
cas-N6512_RFC	0.8787	512	2	cas-N6512_RFC	0.892	UNHASHED	1	0.022891293	0.0133	y
cas-N6512_FEST	0.8741	128	1	cas-N6512_FEST	0.8876	UNHASHED	3	0.023476226	0.0135	y
cas-N6512_FEST	0.8799	2048	3	cas-N6512_FEST	0.8936	UNHASHED	1	0.018368689	0.0137	y
cas-N6512_RFC	0.8742	256	2	cas-N6512_RFC	0.8882	UNHASHED	3	0.01862462	0.014	y
cas-N6512_RFC	0.8766	1024	3	cas-N6512_RFC	0.8908	UNHASHED	2	0.015865822	0.0142	y
cas-N6512_RFC	0.8776	256	1	cas-N6512_RFC	0.892	UNHASHED	1	0.013987602	0.0144	y
cas-N6512_FEST	0.875	256	2	cas-N6512_FEST	0.8899	UNHASHED	2	0.011820086	0.0149	y
cas-N6512_RFC	0.8766	1024	3	cas-N6512_RFC	0.892	UNHASHED	1	0.008725806	0.0154	y
cas-N6512_FEST	0.8741	128	1	cas-N6512_FEST	0.8899	UNHASHED	2	0.007710398	0.0158	y
cas-N6512_FEST	0.8775	1024	3	cas-N6512_FEST	0.8936	UNHASHED	1	0.005837951	0.0161	y
cas-N6512_RFC	0.8742	256	2	cas-N6512_RFC	0.8908	UNHASHED	2	0.005025975	0.0166	y
cas-N6512_FEST	0.8707	512	3	cas-N6512_FEST	0.8876	UNHASHED	3	0.004856086	0.0169	y
cas-N6512_RFC	0.8742	256	2	cas-N6512_RFC	0.892	UNHASHED	1	0.002562254	0.0178	y
cas-N6512_RFC	0.8697	512	3	cas-N6512_RFC	0.8882	UNHASHED	3	0.002065756	0.0185	y
cas-N6512_FEST	0.875	256	2	cas-N6512_FEST	0.8936	UNHASHED	1	0.001536475	0.0186	y
cas-N6512_FEST	0.8707	512	3	cas-N6512_FEST	0.8899	UNHASHED	2	0.001304186	0.0192	y
cas-N6512_FEST	0.8741	128	1	cas-N6512_FEST	0.8936	UNHASHED	1	0.000917825	0.0195	y
sr-mmp_RFC	0.8869	4096	3	sr-mmp_RFC	0.9069	UNHASHED	2	0.048137431	0.02	y
sr-mmp_RFC	0.8859	2048	2	sr-mmp_RFC	0.9061	UNHASHED	3	0.046755911	0.0202	y
cas-N6512_RFC	0.8679	128	1	cas-N6512_RFC	0.8882	UNHASHED	3	0.000757209	0.0203	y

sr-mmp_FEST	0.8872	4096	3	sr-mmp_FEST	0.9078	UNHASHED	2	0.04128674	0.0206	y
sr-mmp_SVM	0.8896	4096	3	sr-mmp_SVM	0.9102	UNHASHED	1	0.039245564	0.0206	y
sr-mmp_SVM	0.892	4096	2	sr-mmp_SVM	0.9129	UNHASHED	3	0.034413235	0.0209	y
sr-mmp_RFC	0.8859	2048	2	sr-mmp_RFC	0.9069	UNHASHED	2	0.038377901	0.021	y
sr-mmp_FEST	0.8846	4096	2	sr-mmp_FEST	0.9057	UNHASHED	3	0.038453004	0.0211	y
cas-N6512_RFC	0.8697	512	3	cas-N6512_RFC	0.8908	UNHASHED	2	0.000411715	0.0211	y
nr-ahr_RFC	0.8997	512	3	nr-ahr_RFC	0.9209	UNHASHED	1	0.046486219	0.0212	y
nr-ahr_SVM	0.8967	512	2	nr-ahr_SVM	0.918	UNHASHED	3	0.048561995	0.0213	y
sr-mmp_RFC	0.8885	4096	2	sr-mmp_RFC	0.9099	UNHASHED	1	0.032732376	0.0214	y
sr-mmp_SVM	0.8896	4096	3	sr-mmp_SVM	0.9114	UNHASHED	2	0.028700716	0.0218	y
nr-ahr_FEST	0.895	128	1	nr-ahr_FEST	0.9169	UNHASHED	1	0.043909273	0.0219	y
sr-mmp_RFC	0.8841	1024	2	sr-mmp_RFC	0.9061	UNHASHED	3	0.0309289	0.022	y
sr-mmp_RFC	0.8841	1024	3	sr-mmp_RFC	0.9061	UNHASHED	3	0.0309289	0.022	y
sr-mmp_RFC	0.8841	2048	3	sr-mmp_RFC	0.9061	UNHASHED	3	0.0309289	0.022	y
sr-mmp_SVM	0.888	2048	2	sr-mmp_SVM	0.9102	UNHASHED	1	0.026803826	0.0222	y
cas-N6512_RFC	0.8697	512	3	cas-N6512_RFC	0.892	UNHASHED	1	0.000182159	0.0223	y
nr-ahr_FEST	0.8915	256	3	nr-ahr_FEST	0.9139	UNHASHED	3	0.042246009	0.0224	y
sr-mmp_FEST	0.8832	2048	2	sr-mmp_FEST	0.9057	UNHASHED	3	0.027716018	0.0225	y
sr-mmp_SVM	0.8877	4096	1	sr-mmp_SVM	0.9102	UNHASHED	1	0.024903258	0.0225	y
nr-ahr_RFC	0.8983	128	1	nr-ahr_RFC	0.9209	UNHASHED	1	0.034386057	0.0226	y
nr-ahr_SVM	0.8885	128	1	nr-ahr_SVM	0.9112	UNHASHED	2	0.042048441	0.0227	y
nr-ahr_RFC	0.8922	256	3	nr-ahr_RFC	0.9149	UNHASHED	3	0.038816739	0.0227	y
sr-mmp_RFC	0.8841	1024	2	sr-mmp_RFC	0.9069	UNHASHED	2	0.025074043	0.0228	y
sr-mmp_RFC	0.8841	1024	3	sr-mmp_RFC	0.9069	UNHASHED	2	0.025074043	0.0228	y
sr-mmp_RFC	0.8841	2048	3	sr-mmp_RFC	0.9069	UNHASHED	2	0.025074043	0.0228	y
sr-mmp_FEST	0.8828	1024	2	sr-mmp_FEST	0.9057	UNHASHED	3	0.02517736	0.0229	y
cas-N6512_FEST	0.8707	512	3	cas-N6512_FEST	0.8936	UNHASHED	1	0.000111487	0.0229	y
cas-N6512_RFC	0.8679	128	1	cas-N6512_RFC	0.8908	UNHASHED	2	0.000133797	0.0229	y
sr-mmp_RFC	0.8869	4096	3	sr-mmp_RFC	0.9099	UNHASHED	1	0.022168462	0.023	y
sr-mmp_RFC	0.883	4096	1	sr-mmp_RFC	0.9061	UNHASHED	3	0.023758965	0.0231	y
sr-mmp_FEST	0.8872	4096	3	sr-mmp_FEST	0.9103	UNHASHED	1	0.021399774	0.0231	y
nr-ahr_FEST	0.8907	128	2	nr-ahr_FEST	0.9139	UNHASHED	3	0.035722772	0.0232	y
sr-mmp_FEST	0.8825	2048	3	sr-mmp_FEST	0.9057	UNHASHED	3	0.023410038	0.0232	y

sr-mmp_FEST	0.8846	4096	2	sr-mmp_FEST	0.9078	UNHASHED	2	0.022241404	0.0232	y
cas-N6512_SVM	0.773	4096	2	cas-N6512_SVM	0.7962	UNHASHED	1	0.00329637	0.0232	y
nr-ahr_RFC	0.8916	128	2	nr-ahr_RFC	0.9149	UNHASHED	3	0.034173785	0.0233	y
sr-mmp_SVM	0.8896	4096	3	sr-mmp_SVM	0.9129	UNHASHED	3	0.01897343	0.0233	y
sr-mmp_SVM	0.888	2048	2	sr-mmp_SVM	0.9114	UNHASHED	2	0.019267989	0.0234	y
sr-mmp_RFC	0.8826	1024	1	sr-mmp_RFC	0.9061	UNHASHED	3	0.021541109	0.0235	y
sr-mmp_SVM	0.8877	4096	1	sr-mmp_SVM	0.9114	UNHASHED	2	0.017844113	0.0237	y
cas-N6512_SVM	0.7725	2048	2	cas-N6512_SVM	0.7962	UNHASHED	1	0.00269542	0.0237	y
sr-mmp_RFC	0.883	4096	1	sr-mmp_RFC	0.9069	UNHASHED	2	0.019115654	0.0239	y
sr-mmp_RFC	0.8859	2048	2	sr-mmp_RFC	0.9099	UNHASHED	1	0.017215525	0.024	y
sr-mmp_RFC	0.882	2048	1	sr-mmp_RFC	0.9061	UNHASHED	3	0.018557616	0.0241	y
cas-N6512_RFC	0.8679	128	1	cas-N6512_RFC	0.892	UNHASHED	1	5.60E-05	0.0241	y
nr-ahr_SVM	0.8885	128	1	nr-ahr_SVM	0.9128	UNHASHED	1	0.028953422	0.0243	y
sr-mmp_RFC	0.8826	1024	1	sr-mmp_RFC	0.9069	UNHASHED	2	0.017283403	0.0243	y
nr-ahr_SVM	0.8935	1024	3	nr-ahr_SVM	0.918	UNHASHED	3	0.024282136	0.0245	y
nr-ahr_RFC	0.8922	256	3	nr-ahr_RFC	0.9167	UNHASHED	2	0.025133225	0.0245	y
sr-mmp_SVM	0.8857	1024	1	sr-mmp_SVM	0.9102	UNHASHED	1	0.01500267	0.0245	y
sr-mmp_SVM	0.8857	2048	3	sr-mmp_SVM	0.9102	UNHASHED	1	0.01500267	0.0245	y
sr-mmp_FEST	0.8832	2048	2	sr-mmp_FEST	0.9078	UNHASHED	2	0.015628674	0.0246	y
nr-ahr_FEST	0.8915	256	3	nr-ahr_FEST	0.9162	UNHASHED	2	0.024348281	0.0247	y
cas-N6512_SVM	0.773	4096	2	cas-N6512_SVM	0.7978	UNHASHED	2	0.001651658	0.0248	y
nr-ahr_SVM	0.8864	128	2	nr-ahr_SVM	0.9112	UNHASHED	2	0.026990175	0.0248	y
nr-ahr_SVM	0.8863	512	3	nr-ahr_SVM	0.9112	UNHASHED	2	0.026409912	0.0249	y
sr-mmp_RFC	0.882	2048	1	sr-mmp_RFC	0.9069	UNHASHED	2	0.014827976	0.0249	y
sr-mmp_SVM	0.888	2048	2	sr-mmp_SVM	0.9129	UNHASHED	3	0.012463336	0.0249	y
sr-mmp_FEST	0.8807	2048	1	sr-mmp_FEST	0.9057	UNHASHED	3	0.014929979	0.025	y
sr-mmp_FEST	0.8828	1024	2	sr-mmp_FEST	0.9078	UNHASHED	2	0.014094305	0.025	y
sr-mmp_FEST	0.8806	1024	3	sr-mmp_FEST	0.9057	UNHASHED	3	0.014551964	0.0251	y
nr-ahr_RFC	0.8916	128	2	nr-ahr_RFC	0.9167	UNHASHED	2	0.021950518	0.0251	y
sr-mmp_RFC	0.8809	512	2	sr-mmp_RFC	0.9061	UNHASHED	3	0.014028303	0.0252	y
sr-mmp_SVM	0.8877	4096	1	sr-mmp_SVM	0.9129	UNHASHED	3	0.011495266	0.0252	y
cas-N6512_SVM	0.773	4096	2	cas-N6512_SVM	0.7982	UNHASHED	3	0.001380352	0.0252	y
cas-N6512_SVM	0.7725	2048	2	cas-N6512_SVM	0.7978	UNHASHED	2	0.001334513	0.0253	y

sr-mmp_FEST	0.8825	2048	3	sr-mmp_FEST	0.9078	UNHASHED	2	0.013033662	0.0253	y
nr-ahr_FEST	0.8915	256	3	nr-ahr_FEST	0.9169	UNHASHED	1	0.020384316	0.0254	y
nr-ahr_FEST	0.8907	128	2	nr-ahr_FEST	0.9162	UNHASHED	2	0.020311595	0.0255	y
sr-mmp_FEST	0.8801	4096	1	sr-mmp_FEST	0.9057	UNHASHED	3	0.012787482	0.0256	y
nr-ahr_SVM	0.8923	256	2	nr-ahr_SVM	0.918	UNHASHED	3	0.01843443	0.0257	y
sr-mmp_FEST	0.8846	4096	2	sr-mmp_FEST	0.9103	UNHASHED	1	0.010885671	0.0257	y
sr-mmp_SVM	0.8857	1024	1	sr-mmp_SVM	0.9114	UNHASHED	2	0.010521095	0.0257	y
sr-mmp_SVM	0.8857	2048	3	sr-mmp_SVM	0.9114	UNHASHED	2	0.010521095	0.0257	y
cas-N6512_SVM	0.7725	2048	2	cas-N6512_SVM	0.7982	UNHASHED	3	0.001111962	0.0257	y
cas-N6512_FEST	0.8618	128	2	cas-N6512_FEST	0.8876	UNHASHED	3	2.42E-05	0.0258	y
sr-mmp_RFC	0.8841	1024	2	sr-mmp_RFC	0.9099	UNHASHED	1	0.010727132	0.0258	y
sr-mmp_RFC	0.8841	1024	3	sr-mmp_RFC	0.9099	UNHASHED	1	0.010727132	0.0258	y
sr-mmp_RFC	0.8841	2048	3	sr-mmp_RFC	0.9099	UNHASHED	1	0.010727132	0.0258	y
sr-mmp_RFC	0.8809	512	2	sr-mmp_RFC	0.9069	UNHASHED	2	0.011123975	0.026	y
nr-ahr_FEST	0.8907	128	2	nr-ahr_FEST	0.9169	UNHASHED	1	0.016933813	0.0262	y
sr-mmp_RFC	0.8799	512	1	sr-mmp_RFC	0.9061	UNHASHED	3	0.010799082	0.0262	y
nr-ahr_SVM	0.8864	128	2	nr-ahr_SVM	0.9128	UNHASHED	1	0.018142546	0.0264	y
sr-mmp_SVM	0.8838	2048	1	sr-mmp_SVM	0.9102	UNHASHED	1	0.009029313	0.0264	y
nr-ahr_SVM	0.8863	512	3	nr-ahr_SVM	0.9128	UNHASHED	1	0.017732138	0.0265	y
cas-N6512_RFC	0.8617	128	2	cas-N6512_RFC	0.8882	UNHASHED	3	1.42E-05	0.0265	y
sr-mmp_SVM	0.8834	512	1	sr-mmp_SVM	0.9102	UNHASHED	1	0.008087665	0.0268	y
cas-N6512_SVM	0.7694	1024	2	cas-N6512_SVM	0.7962	UNHASHED	1	0.000718265	0.0268	y
cas-N6512_FEST	0.8607	256	3	cas-N6512_FEST	0.8876	UNHASHED	3	1.12E-05	0.0269	y
sr-mmp_RFC	0.883	4096	1	sr-mmp_RFC	0.9099	UNHASHED	1	0.007944339	0.0269	y
sr-mmp_SVM	0.8833	1024	2	sr-mmp_SVM	0.9102	UNHASHED	1	0.007866638	0.0269	y
sr-mmp_FEST	0.8787	512	2	sr-mmp_FEST	0.9057	UNHASHED	3	0.008823471	0.027	y
sr-mmp_RFC	0.8799	512	1	sr-mmp_RFC	0.9069	UNHASHED	2	0.00850425	0.027	y
sr-mmp_FEST	0.8807	2048	1	sr-mmp_FEST	0.9078	UNHASHED	2	0.00804449	0.0271	y
sr-mmp_FEST	0.8832	2048	2	sr-mmp_FEST	0.9103	UNHASHED	1	0.007416259	0.0271	y
nr-ahr_SVM	0.884	256	3	nr-ahr_SVM	0.9112	UNHASHED	2	0.015774414	0.0272	y
sr-mmp_FEST	0.8806	1024	3	sr-mmp_FEST	0.9078	UNHASHED	2	0.007826549	0.0272	y
sr-mmp_SVM	0.8857	1024	1	sr-mmp_SVM	0.9129	UNHASHED	3	0.006595648	0.0272	y
sr-mmp_SVM	0.8857	2048	3	sr-mmp_SVM	0.9129	UNHASHED	3	0.006595648	0.0272	y

sr-mmp_RFC	0.8826	1024	1	sr-mmp_RFC	0.9099	UNHASHED	1	0.007107519	0.0273	y
sr-mmp_FEST	0.8828	1024	2	sr-mmp_FEST	0.9103	UNHASHED	1	0.00662931	0.0275	y
sr-mmp_FEST	0.8781	512	1	sr-mmp_FEST	0.9057	UNHASHED	3	0.007495442	0.0276	y
sr-mmp_SVM	0.8838	2048	1	sr-mmp_SVM	0.9114	UNHASHED	2	0.006203886	0.0276	y
sr-mmp_RFC	0.8784	256	1	sr-mmp_RFC	0.9061	UNHASHED	3	0.00720068	0.0277	y
sr-mmp_FEST	0.8801	4096	1	sr-mmp_FEST	0.9078	UNHASHED	2	0.00681523	0.0277	y
sr-mmp_FEST	0.8825	2048	3	sr-mmp_FEST	0.9103	UNHASHED	1	0.006089934	0.0278	y
sr-mmp_RFC	0.882	2048	1	sr-mmp_RFC	0.9099	UNHASHED	1	0.006002061	0.0279	y
sr-mmp_SVM	0.8834	512	1	sr-mmp_SVM	0.9114	UNHASHED	2	0.005533025	0.028	y
cas-N6512_RFC	0.8602	256	3	cas-N6512_RFC	0.8882	UNHASHED	3	4.84E-06	0.028	y
sr-mmp_FEST	0.8776	1024	1	sr-mmp_FEST	0.9057	UNHASHED	3	0.006530556	0.0281	y
sr-mmp_RFC	0.878	512	3	sr-mmp_RFC	0.9061	UNHASHED	3	0.006446302	0.0281	y
sr-mmp_SVM	0.8833	1024	2	sr-mmp_SVM	0.9114	UNHASHED	2	0.005376025	0.0281	y
cas-N6512_FEST	0.8618	128	2	cas-N6512_FEST	0.8899	UNHASHED	2	3.88E-06	0.0281	y
cas-N6512_SVM	0.7694	1024	2	cas-N6512_SVM	0.7978	UNHASHED	2	0.000330244	0.0284	y
sr-mmp_RFC	0.8784	256	1	sr-mmp_RFC	0.9069	UNHASHED	2	0.005611952	0.0285	y
nr-ahr_RFC	0.8922	256	3	nr-ahr_RFC	0.9209	UNHASHED	1	0.008055666	0.0287	y
nr-ahr_SVM	0.884	256	3	nr-ahr_SVM	0.9128	UNHASHED	1	0.010315252	0.0288	y
cas-N6512_SVM	0.7694	1024	2	cas-N6512_SVM	0.7982	UNHASHED	3	0.000270112	0.0288	y
sr-mmp_RFC	0.878	512	3	sr-mmp_RFC	0.9069	UNHASHED	2	0.005010131	0.0289	y
cas-N6512_SVM	0.7673	2048	3	cas-N6512_SVM	0.7962	UNHASHED	1	0.00027274	0.0289	y
sr-mmp_RFC	0.8809	512	2	sr-mmp_RFC	0.9099	UNHASHED	1	0.004374049	0.029	y
sr-mmp_FEST	0.8766	512	3	sr-mmp_FEST	0.9057	UNHASHED	3	0.004932314	0.0291	y
sr-mmp_FEST	0.8787	512	2	sr-mmp_FEST	0.9078	UNHASHED	2	0.004584231	0.0291	y
sr-mmp_SVM	0.8838	2048	1	sr-mmp_SVM	0.9129	UNHASHED	3	0.003789934	0.0291	y
cas-N6512_RFC	0.8617	128	2	cas-N6512_RFC	0.8908	UNHASHED	2	1.69E-06	0.0291	y
cas-N6512_FEST	0.8607	256	3	cas-N6512_FEST	0.8899	UNHASHED	2	1.70E-06	0.0292	y
nr-ahr_RFC	0.8916	128	2	nr-ahr_RFC	0.9209	UNHASHED	1	0.006901614	0.0293	y
nr-ahr_SVM	0.8885	128	1	nr-ahr_SVM	0.918	UNHASHED	3	0.00729005	0.0295	y
sr-mmp_SVM	0.8834	512	1	sr-mmp_SVM	0.9129	UNHASHED	3	0.003361776	0.0295	y
sr-mmp_SVM	0.8806	1024	3	sr-mmp_SVM	0.9102	UNHASHED	1	0.003626091	0.0296	y
sr-mmp_FEST	0.8807	2048	1	sr-mmp_FEST	0.9103	UNHASHED	1	0.003612257	0.0296	y
sr-mmp_SVM	0.8833	1024	2	sr-mmp_SVM	0.9129	UNHASHED	3	0.003261949	0.0296	y

sr-mmp_FEST	0.8806	1024	3	sr-mmp_FEST	0.9103	UNHASHED	1	0.003506644	0.0297	y
sr-mmp_FEST	0.8781	512	1	sr-mmp_FEST	0.9078	UNHASHED	2	0.003851964	0.0297	y
sr-mmp_RFC	0.8799	512	1	sr-mmp_RFC	0.9099	UNHASHED	1	0.003256974	0.03	y
sr-mmp_FEST	0.8776	1024	1	sr-mmp_FEST	0.9078	UNHASHED	2	0.003325712	0.0302	y
sr-mmp_FEST	0.8801	4096	1	sr-mmp_FEST	0.9103	UNHASHED	1	0.003020016	0.0302	y
cas-N6512_RFC	0.8617	128	2	cas-N6512_RFC	0.892	UNHASHED	1	5.89E-07	0.0303	y
sr-mmp_FEST	0.8753	256	1	sr-mmp_FEST	0.9057	UNHASHED	3	0.003390085	0.0304	y
cas-N6512_SVM	0.7673	2048	3	cas-N6512_SVM	0.7978	UNHASHED	2	0.000119301	0.0305	y
cas-N6512_RFC	0.8602	256	3	cas-N6512_RFC	0.8908	UNHASHED	2	5.25E-07	0.0306	y
sr-mmp_RFC	0.8754	256	2	sr-mmp_RFC	0.9061	UNHASHED	3	0.003058087	0.0307	y
sr-mmp_SVM	0.8806	1024	3	sr-mmp_SVM	0.9114	UNHASHED	2	0.002407178	0.0308	y
sr-mmp_SVM	0.8793	256	1	sr-mmp_SVM	0.9102	UNHASHED	1	0.002453018	0.0309	y
cas-N6512_SVM	0.7673	2048	3	cas-N6512_SVM	0.7982	UNHASHED	3	9.64E-05	0.0309	y
nr-er_SVM	0.765	512	2	nr-er_SVM	0.7962	UNHASHED	1	0.049522832	0.0312	y
sr-mmp_FEST	0.8766	512	3	sr-mmp_FEST	0.9078	UNHASHED	2	0.002466547	0.0312	y
cas-N6512_SVM	0.765	512	2	cas-N6512_SVM	0.7962	UNHASHED	1	8.84E-05	0.0312	y
nr-er_SVM	0.7649	4096	3	nr-er_SVM	0.7962	UNHASHED	1	0.048811607	0.0313	y
nr-er_RFC	0.7658	256	3	nr-er_RFC	0.7971	UNHASHED	3	0.048529695	0.0313	y
cas-N6512_SVM	0.7649	4096	3	cas-N6512_SVM	0.7962	UNHASHED	1	8.40E-05	0.0313	y
nr-er_SVM	0.7648	256	2	nr-er_SVM	0.7962	UNHASHED	1	0.048109054	0.0314	y
cas-N6512_SVM	0.7648	256	2	cas-N6512_SVM	0.7962	UNHASHED	1	7.99E-05	0.0314	y
sr-mmp_RFC	0.8754	256	2	sr-mmp_RFC	0.9069	UNHASHED	2	0.00233445	0.0315	y
sr-mmp_RFC	0.8784	256	1	sr-mmp_RFC	0.9099	UNHASHED	1	0.00206605	0.0315	y
nr-ahr_SVM	0.8864	128	2	nr-ahr_SVM	0.918	UNHASHED	3	0.004213862	0.0316	y
nr-er_FEST	0.7647	512	1	nr-er_FEST	0.7963	UNHASHED	2	0.04669926	0.0316	y
nr-er_RFC	0.7658	256	3	nr-er_RFC	0.7974	UNHASHED	2	0.046364107	0.0316	y
sr-mmp_FEST	0.8787	512	2	sr-mmp_FEST	0.9103	UNHASHED	1	0.001969652	0.0316	y
nr-ahr_SVM	0.8863	512	3	nr-ahr_SVM	0.918	UNHASHED	3	0.00410275	0.0317	y
nr-er_FEST	0.7646	256	2	nr-er_FEST	0.7963	UNHASHED	2	0.046022535	0.0317	y
nr-er_FEST	0.7647	512	1	nr-er_FEST	0.7965	UNHASHED	1	0.045294509	0.0318	y
cas-N6512_FEST	0.8618	128	2	cas-N6512_FEST	0.8936	UNHASHED	1	1.47E-07	0.0318	y
cas-N6512_RFC	0.8602	256	3	cas-N6512_RFC	0.892	UNHASHED	1	1.76E-07	0.0318	y
nr-er_FEST	0.7646	256	2	nr-er_FEST	0.7965	UNHASHED	1	0.044634986	0.0319	y

nr-er_RFC	0.765	512	1	nr-er_RFC	0.7969	UNHASHED	1	0.044516675	0.0319	y
sr-mmp_RFC	0.878	512	3	sr-mmp_RFC	0.9099	UNHASHED	1	0.001825186	0.0319	y
nr-er_RFC	0.765	512	1	nr-er_RFC	0.7971	UNHASHED	3	0.043166333	0.0321	y
sr-mmp_SVM	0.8793	256	1	sr-mmp_SVM	0.9114	UNHASHED	2	0.001605903	0.0321	y
sr-mmp_FEST	0.8735	256	2	sr-mmp_FEST	0.9057	UNHASHED	3	0.001979951	0.0322	y
sr-mmp_FEST	0.8781	512	1	sr-mmp_FEST	0.9103	UNHASHED	1	0.001633307	0.0322	y
sr-mmp_SVM	0.8806	1024	3	sr-mmp_SVM	0.9129	UNHASHED	3	0.001408062	0.0323	y
nr-er_RFC	0.7646	256	2	nr-er_RFC	0.7969	UNHASHED	1	0.041964279	0.0323	y
nr-er_FEST	0.7589	128	2	nr-er_FEST	0.7913	UNHASHED	3	0.042905226	0.0324	y
nr-er_RFC	0.765	512	1	nr-er_RFC	0.7974	UNHASHED	2	0.041205014	0.0324	y
nr-er_RFC	0.7645	4096	1	nr-er_RFC	0.7969	UNHASHED	1	0.041346061	0.0324	y
nr-ahr_RFC	0.8825	128	3	nr-ahr_RFC	0.9149	UNHASHED	3	0.003845862	0.0324	y
nr-er_FEST	0.7638	256	1	nr-er_FEST	0.7963	UNHASHED	2	0.040902829	0.0325	y
nr-ahr_SVM	0.8787	128	3	nr-ahr_SVM	0.9112	UNHASHED	2	0.004303725	0.0325	y
nr-er_RFC	0.7646	256	2	nr-er_RFC	0.7971	UNHASHED	3	0.040679819	0.0325	y
sr-mmp_FEST	0.8753	256	1	sr-mmp_FEST	0.9078	UNHASHED	2	0.001655766	0.0325	y
nr-er_RFC	0.7645	4096	1	nr-er_RFC	0.7971	UNHASHED	3	0.040077679	0.0326	y
nr-er_FEST	0.7638	256	1	nr-er_FEST	0.7965	UNHASHED	1	0.039647175	0.0327	y
sr-mmp_FEST	0.8776	1024	1	sr-mmp_FEST	0.9103	UNHASHED	1	0.00139474	0.0327	y
nr-er_SVM	0.765	512	2	nr-er_SVM	0.7978	UNHASHED	2	0.038706356	0.0328	y
cas-N6512_SVM	0.765	512	2	cas-N6512_SVM	0.7978	UNHASHED	2	3.66E-05	0.0328	y
nr-er_RFC	0.7646	256	2	nr-er_RFC	0.7974	UNHASHED	2	0.038814934	0.0328	y
nr-er_SVM	0.7649	4096	3	nr-er_SVM	0.7978	UNHASHED	2	0.038128845	0.0329	y
cas-N6512_SVM	0.7649	4096	3	cas-N6512_SVM	0.7978	UNHASHED	2	3.47E-05	0.0329	y
cas-N6512_FEST	0.8607	256	3	cas-N6512_FEST	0.8936	UNHASHED	1	5.89E-08	0.0329	y
nr-er_RFC	0.7645	4096	1	nr-er_RFC	0.7974	UNHASHED	2	0.03823632	0.0329	y
nr-er_SVM	0.7648	256	2	nr-er_SVM	0.7978	UNHASHED	2	0.03755874	0.033	y
cas-N6512_SVM	0.7648	256	2	cas-N6512_SVM	0.7978	UNHASHED	2	3.30E-05	0.033	y
nr-er_RFC	0.7639	2048	1	nr-er_RFC	0.7969	UNHASHED	1	0.037797857	0.033	y
nr-er_RFC	0.7639	2048	1	nr-er_RFC	0.7971	UNHASHED	3	0.036622708	0.0332	y
nr-er_SVM	0.765	512	2	nr-er_SVM	0.7982	UNHASHED	3	0.036336083	0.0332	y
cas-N6512_SVM	0.765	512	2	cas-N6512_SVM	0.7982	UNHASHED	3	2.92E-05	0.0332	y
nr-er_SVM	0.7649	4096	3	nr-er_SVM	0.7982	UNHASHED	3	0.035788832	0.0333	y

cas-N6512_SVM	0.7649	4096	3	cas-N6512_SVM	0.7982	UNHASHED	3	2.77E-05	0.0333	y
nr-er_SVM	0.7648	256	2	nr-er_SVM	0.7982	UNHASHED	3	0.035248686	0.0334	y
cas-N6512_SVM	0.7648	256	2	cas-N6512_SVM	0.7982	UNHASHED	3	2.62E-05	0.0334	y
nr-er_RFC	0.7639	2048	1	nr-er_RFC	0.7974	UNHASHED	2	0.034917716	0.0335	y
nr-er_SVM	0.7626	256	1	nr-er_SVM	0.7962	UNHASHED	1	0.034688354	0.0336	y
sr-mmp_SVM	0.8793	256	1	sr-mmp_SVM	0.9129	UNHASHED	3	0.000923014	0.0336	y
cas-N6512_SVM	0.7626	256	1	cas-N6512_SVM	0.7962	UNHASHED	1	2.54E-05	0.0336	y
sr-mmp_FEST	0.8766	512	3	sr-mmp_FEST	0.9103	UNHASHED	1	0.001011949	0.0337	y
nr-ahr_SVM	0.884	256	3	nr-ahr_SVM	0.918	UNHASHED	3	0.002185077	0.034	y
nr-er_FEST	0.7573	128	3	nr-er_FEST	0.7913	UNHASHED	3	0.033787526	0.034	y
nr-ahr_SVM	0.8787	128	3	nr-ahr_SVM	0.9128	UNHASHED	1	0.002648373	0.0341	y
sr-mmp_FEST	0.8715	128	1	sr-mmp_FEST	0.9057	UNHASHED	3	0.001062344	0.0342	y
nr-ahr_RFC	0.8825	128	3	nr-ahr_RFC	0.9167	UNHASHED	2	0.00218795	0.0342	y
sr-mmp_SVM	0.876	512	2	sr-mmp_SVM	0.9102	UNHASHED	1	0.000863944	0.0342	y
sr-mmp_FEST	0.8735	256	2	sr-mmp_FEST	0.9078	UNHASHED	2	0.000936025	0.0343	y
sr-mmp_RFC	0.8754	256	2	sr-mmp_RFC	0.9099	UNHASHED	1	0.000794368	0.0345	y
nr-er_SVM	0.7616	1024	1	nr-er_SVM	0.7962	UNHASHED	1	0.029743081	0.0346	y
nr-er_SVM	0.7616	2048	1	nr-er_SVM	0.7962	UNHASHED	1	0.029743081	0.0346	y
cas-N6512_SVM	0.7616	1024	1	cas-N6512_SVM	0.7962	UNHASHED	1	1.48E-05	0.0346	y
cas-N6512_SVM	0.7616	2048	1	cas-N6512_SVM	0.7962	UNHASHED	1	1.48E-05	0.0346	y
nr-er_FEST	0.7565	128	1	nr-er_FEST	0.7913	UNHASHED	3	0.029891793	0.0348	y
nr-ahr_FEST	0.8791	128	3	nr-ahr_FEST	0.9139	UNHASHED	3	0.002089217	0.0348	y
sr-mmp_FEST	0.8753	256	1	sr-mmp_FEST	0.9103	UNHASHED	1	0.000660238	0.035	y
nr-er_SVM	0.7626	256	1	nr-er_SVM	0.7978	UNHASHED	2	0.026744139	0.0352	y
sr-mmp_RFC	0.8709	128	1	sr-mmp_RFC	0.9061	UNHASHED	3	0.000756463	0.0352	y
cas-N6512_SVM	0.7626	256	1	cas-N6512_SVM	0.7978	UNHASHED	2	9.95E-06	0.0352	y
nr-er_SVM	0.7609	1024	3	nr-er_SVM	0.7962	UNHASHED	1	0.026656053	0.0353	y
cas-N6512_SVM	0.7609	1024	3	cas-N6512_SVM	0.7962	UNHASHED	1	1.00E-05	0.0353	y
sr-mmp_SVM	0.876	512	2	sr-mmp_SVM	0.9114	UNHASHED	2	0.000546046	0.0354	y
nr-er_SVM	0.7607	4096	1	nr-er_SVM	0.7962	UNHASHED	1	0.025826929	0.0355	y
sr-mmp_FEST	0.8702	256	3	sr-mmp_FEST	0.9057	UNHASHED	3	0.000698954	0.0355	y
sr-mmp_RFC	0.8706	256	3	sr-mmp_RFC	0.9061	UNHASHED	3	0.000685965	0.0355	y
cas-N6512_SVM	0.7607	4096	1	cas-N6512_SVM	0.7962	UNHASHED	1	8.98E-06	0.0355	y

nr-er_RFC	0.7613	256	1	nr-er_RFC	0.7969	UNHASHED	1	0.025280949	0.0356	y
nr-er_SVM	0.7626	256	1	nr-er_SVM	0.7982	UNHASHED	3	0.025020305	0.0356	y
cas-N6512_SVM	0.7626	256	1	cas-N6512_SVM	0.7982	UNHASHED	3	7.82E-06	0.0356	y
nr-er_RFC	0.7613	256	1	nr-er_RFC	0.7971	UNHASHED	3	0.024449526	0.0358	y
nr-er_SVM	0.7602	512	1	nr-er_SVM	0.7962	UNHASHED	1	0.023851746	0.036	y
sr-mmp_RFC	0.8709	128	1	sr-mmp_RFC	0.9069	UNHASHED	2	0.000559887	0.036	y
cas-N6512_SVM	0.7602	512	1	cas-N6512_SVM	0.7962	UNHASHED	1	6.78E-06	0.036	y
nr-er_SVM	0.7601	512	3	nr-er_SVM	0.7962	UNHASHED	1	0.023472949	0.0361	y
nr-er_RFC	0.7613	256	1	nr-er_RFC	0.7974	UNHASHED	2	0.023246351	0.0361	y
cas-N6512_SVM	0.7601	512	3	cas-N6512_SVM	0.7962	UNHASHED	1	6.41E-06	0.0361	y
nr-er_SVM	0.7616	1024	1	nr-er_SVM	0.7978	UNHASHED	2	0.022800621	0.0362	y
nr-er_SVM	0.7616	2048	1	nr-er_SVM	0.7978	UNHASHED	2	0.022800621	0.0362	y
cas-N6512_SVM	0.7616	1024	1	cas-N6512_SVM	0.7978	UNHASHED	2	5.66E-06	0.0362	y
cas-N6512_SVM	0.7616	2048	1	cas-N6512_SVM	0.7978	UNHASHED	2	5.66E-06	0.0362	y
sr-mmp_RFC	0.8706	256	3	sr-mmp_RFC	0.9069	UNHASHED	2	0.00050667	0.0363	y
sr-mmp_FEST	0.8715	128	1	sr-mmp_FEST	0.9078	UNHASHED	2	0.000484444	0.0363	y
nr-er_SVM	0.7616	1024	1	nr-er_SVM	0.7982	UNHASHED	3	0.021300317	0.0366	y
nr-er_SVM	0.7616	2048	1	nr-er_SVM	0.7982	UNHASHED	3	0.021300317	0.0366	y
cas-N6512_SVM	0.7616	1024	1	cas-N6512_SVM	0.7982	UNHASHED	3	4.43E-06	0.0366	y
cas-N6512_SVM	0.7616	2048	1	cas-N6512_SVM	0.7982	UNHASHED	3	4.43E-06	0.0366	y
sr-mmp_FEST	0.8735	256	2	sr-mmp_FEST	0.9103	UNHASHED	1	0.000358875	0.0368	y
nr-er_SVM	0.7609	1024	3	nr-er_SVM	0.7978	UNHASHED	2	0.0203523	0.0369	y
cas-N6512_SVM	0.7609	1024	3	cas-N6512_SVM	0.7978	UNHASHED	2	3.79E-06	0.0369	y
sr-mmp_SVM	0.876	512	2	sr-mmp_SVM	0.9129	UNHASHED	3	0.000300265	0.0369	y
nr-er_SVM	0.7607	4096	1	nr-er_SVM	0.7978	UNHASHED	2	0.019696624	0.0371	y
cas-N6512_SVM	0.7607	4096	1	cas-N6512_SVM	0.7978	UNHASHED	2	3.37E-06	0.0371	y
nr-ahr_FEST	0.8791	128	3	nr-ahr_FEST	0.9162	UNHASHED	2	0.000976421	0.0371	y
nr-er_RFC	0.7596	128	1	nr-er_RFC	0.7969	UNHASHED	1	0.019207149	0.0373	y
nr-er_SVM	0.7609	1024	3	nr-er_SVM	0.7982	UNHASHED	3	0.018993924	0.0373	y
cas-N6512_SVM	0.7609	1024	3	cas-N6512_SVM	0.7982	UNHASHED	3	2.95E-06	0.0373	y
nr-er_FEST	0.7589	128	2	nr-er_FEST	0.7963	UNHASHED	2	0.018990699	0.0374	y
nr-er_RFC	0.7596	128	1	nr-er_RFC	0.7971	UNHASHED	3	0.018552811	0.0375	y
nr-er_SVM	0.7607	4096	1	nr-er_SVM	0.7982	UNHASHED	3	0.018376705	0.0375	y

cas-N6512_SVM	0.7607	4096	1	cas-N6512_SVM	0.7982	UNHASHED	3	2.62E-06	0.0375	y
nr-er_FEST	0.7589	128	2	nr-er_FEST	0.7965	UNHASHED	1	0.018343426	0.0376	y
nr-er_SVM	0.7602	512	1	nr-er_SVM	0.7978	UNHASHED	2	0.018138088	0.0376	y
cas-N6512_SVM	0.7602	512	1	cas-N6512_SVM	0.7978	UNHASHED	2	2.52E-06	0.0376	y
sr-mmp_FEST	0.8702	256	3	sr-mmp_FEST	0.9078	UNHASHED	2	0.00031139	0.0376	y
nr-er_SVM	0.7601	512	3	nr-er_SVM	0.7978	UNHASHED	2	0.01783977	0.0377	y
cas-N6512_SVM	0.7601	512	3	cas-N6512_SVM	0.7978	UNHASHED	2	2.38E-06	0.0377	y
nr-er_RFC	0.7596	128	1	nr-er_RFC	0.7974	UNHASHED	2	0.017607495	0.0378	y
nr-ahr_FEST	0.8791	128	3	nr-ahr_FEST	0.9169	UNHASHED	1	0.000766377	0.0378	y
nr-er_SVM	0.7602	512	1	nr-er_SVM	0.7982	UNHASHED	3	0.016910394	0.038	y
cas-N6512_SVM	0.7602	512	1	cas-N6512_SVM	0.7982	UNHASHED	3	1.96E-06	0.038	y
nr-er_SVM	0.7601	512	3	nr-er_SVM	0.7982	UNHASHED	3	0.016629864	0.0381	y
cas-N6512_SVM	0.7601	512	3	cas-N6512_SVM	0.7982	UNHASHED	3	1.84E-06	0.0381	y
nr-ahr_RFC	0.8825	128	3	nr-ahr_RFC	0.9209	UNHASHED	1	0.000514897	0.0384	y
sr-mmp_FEST	0.8715	128	1	sr-mmp_FEST	0.9103	UNHASHED	1	0.000177868	0.0388	y
nr-er_FEST	0.7573	128	3	nr-er_FEST	0.7963	UNHASHED	2	0.01453628	0.039	y
sr-mmp_RFC	0.8709	128	1	sr-mmp_RFC	0.9099	UNHASHED	1	0.000169503	0.039	y
nr-er_FEST	0.7573	128	3	nr-er_FEST	0.7965	UNHASHED	1	0.014024661	0.0392	y
nr-ahr_SVM	0.8787	128	3	nr-ahr_SVM	0.918	UNHASHED	3	0.000458339	0.0393	y
sr-mmp_RFC	0.8706	256	3	sr-mmp_RFC	0.9099	UNHASHED	1	0.000152213	0.0393	y
sr-mmp_SVM	0.8707	512	3	sr-mmp_SVM	0.9102	UNHASHED	1	0.000139121	0.0395	y
nr-er_SVM	0.7565	128	1	nr-er_SVM	0.7962	UNHASHED	1	0.012910441	0.0397	y
cas-N6512_SVM	0.7565	128	1	cas-N6512_SVM	0.7962	UNHASHED	1	7.76E-07	0.0397	y
nr-er_FEST	0.7565	128	1	nr-er_FEST	0.7963	UNHASHED	2	0.012678324	0.0398	y
sr-mmp_FEST	0.8657	128	2	sr-mmp_FEST	0.9057	UNHASHED	3	0.000151011	0.04	y
nr-er_FEST	0.7565	128	1	nr-er_FEST	0.7965	UNHASHED	1	0.012225033	0.04	y
nr-er_RFC	0.7569	128	2	nr-er_RFC	0.7969	UNHASHED	1	0.012178607	0.04	y
sr-mmp_FEST	0.8702	256	3	sr-mmp_FEST	0.9103	UNHASHED	1	0.000111182	0.0401	y
nr-er_RFC	0.7569	128	2	nr-er_RFC	0.7971	UNHASHED	3	0.011740799	0.0402	y
nr-er_RFC	0.7566	128	3	nr-er_RFC	0.7969	UNHASHED	1	0.01156059	0.0403	y
nr-er_RFC	0.7566	128	3	nr-er_RFC	0.7971	UNHASHED	3	0.011142577	0.0405	y
nr-er_RFC	0.7569	128	2	nr-er_RFC	0.7974	UNHASHED	2	0.011109991	0.0405	y
sr-mmp_SVM	0.8696	256	2	sr-mmp_SVM	0.9102	UNHASHED	1	9.31E-05	0.0406	y

sr-mmp_SVM	0.8707	512	3	sr-mmp_SVM	0.9114	UNHASHED	2	8.32E-05	0.0407	y
nr-er_RFC	0.7566	128	3	nr-er_RFC	0.7974	UNHASHED	2	0.010540469	0.0408	y
nr-er_SVM	0.7565	128	1	nr-er_SVM	0.7978	UNHASHED	2	0.009610189	0.0413	y
cas-N6512_SVM	0.7565	128	1	cas-N6512_SVM	0.7978	UNHASHED	2	2.66E-07	0.0413	y
nr-er_SVM	0.7546	128	2	nr-er_SVM	0.7962	UNHASHED	1	0.009259363	0.0416	y
sr-mmp_RFC	0.8645	128	2	sr-mmp_RFC	0.9061	UNHASHED	3	8.30E-05	0.0416	y
cas-N6512_SVM	0.7546	128	2	cas-N6512_SVM	0.7962	UNHASHED	1	2.40E-07	0.0416	y
nr-er_SVM	0.7565	128	1	nr-er_SVM	0.7982	UNHASHED	3	0.008911732	0.0417	y
cas-N6512_SVM	0.7565	128	1	cas-N6512_SVM	0.7982	UNHASHED	3	2.03E-07	0.0417	y
sr-mmp_SVM	0.8696	256	2	sr-mmp_SVM	0.9114	UNHASHED	2	5.51E-05	0.0418	y
cas-N6512_FEST	0.8456	128	3	cas-N6512_FEST	0.8876	UNHASHED	3	4.37E-11	0.042	y
sr-mmp_SVM	0.8682	128	1	sr-mmp_SVM	0.9102	UNHASHED	1	5.53E-05	0.042	y
sr-mmp_FEST	0.8657	128	2	sr-mmp_FEST	0.9078	UNHASHED	2	6.21E-05	0.0421	y
sr-mmp_SVM	0.8707	512	3	sr-mmp_SVM	0.9129	UNHASHED	3	4.27E-05	0.0422	y
sr-mmp_RFC	0.8645	128	2	sr-mmp_RFC	0.9069	UNHASHED	2	5.88E-05	0.0424	y
cas-N6512_RFC	0.8452	128	3	cas-N6512_RFC	0.8882	UNHASHED	3	1.59E-11	0.043	y
nr-er_SVM	0.7546	128	2	nr-er_SVM	0.7978	UNHASHED	2	0.00681675	0.0432	y
cas-N6512_SVM	0.7546	128	2	cas-N6512_SVM	0.7978	UNHASHED	2	7.94E-08	0.0432	y
sr-mmp_SVM	0.8682	128	1	sr-mmp_SVM	0.9114	UNHASHED	2	3.22E-05	0.0432	y
sr-mmp_SVM	0.8696	256	2	sr-mmp_SVM	0.9129	UNHASHED	3	2.78E-05	0.0433	y
nr-er_SVM	0.7527	256	3	nr-er_SVM	0.7962	UNHASHED	1	0.006563667	0.0435	y
cas-N6512_SVM	0.7527	256	3	cas-N6512_SVM	0.7962	UNHASHED	1	7.16E-08	0.0435	y
nr-er_SVM	0.7546	128	2	nr-er_SVM	0.7982	UNHASHED	3	0.006303812	0.0436	y
cas-N6512_SVM	0.7546	128	2	cas-N6512_SVM	0.7982	UNHASHED	3	5.99E-08	0.0436	y
cas-N6512_FEST	0.8456	128	3	cas-N6512_FEST	0.8899	UNHASHED	2	3.60E-12	0.0443	y
sr-mmp_FEST	0.8657	128	2	sr-mmp_FEST	0.9103	UNHASHED	1	2.02E-05	0.0446	y
sr-mmp_SVM	0.8655	256	3	sr-mmp_SVM	0.9102	UNHASHED	1	1.95E-05	0.0447	y
sr-mmp_SVM	0.8682	128	1	sr-mmp_SVM	0.9129	UNHASHED	3	1.60E-05	0.0447	y
nr-er_SVM	0.7527	256	3	nr-er_SVM	0.7978	UNHASHED	2	0.004778938	0.0451	y
cas-N6512_SVM	0.7527	256	3	cas-N6512_SVM	0.7978	UNHASHED	2	2.28E-08	0.0451	y
sr-mmp_RFC	0.8645	128	2	sr-mmp_RFC	0.9099	UNHASHED	1	1.51E-05	0.0454	y
nr-er_SVM	0.7527	256	3	nr-er_SVM	0.7982	UNHASHED	3	0.004407061	0.0455	y
cas-N6512_SVM	0.7527	256	3	cas-N6512_SVM	0.7982	UNHASHED	3	1.71E-08	0.0455	y

cas-N6512_RFC	0.8452	128	3	cas-N6512_RFC	0.8908	UNHASHED	2	9.07E-13	0.0456	y
sr-mmp_SVM	0.8655	256	3	sr-mmp_SVM	0.9114	UNHASHED	2	1.11E-05	0.0459	y
cas-N6512_RFC	0.8452	128	3	cas-N6512_RFC	0.892	UNHASHED	1	2.34E-13	0.0468	y
sr-mmp_SVM	0.8655	256	3	sr-mmp_SVM	0.9129	UNHASHED	3	5.33E-06	0.0474	y
cas-N6512_FEST	0.8456	128	3	cas-N6512_FEST	0.8936	UNHASHED	1	5.48E-14	0.048	y
sr-mmp_FEST	0.8558	128	3	sr-mmp_FEST	0.9057	UNHASHED	3	3.38E-06	0.0499	y
sr-mmp_RFC	0.8552	128	3	sr-mmp_RFC	0.9061	UNHASHED	3	2.16E-06	0.0509	y
sr-mmp_RFC	0.8552	128	3	sr-mmp_RFC	0.9069	UNHASHED	2	1.45E-06	0.0517	y
sr-mmp_FEST	0.8558	128	3	sr-mmp_FEST	0.9078	UNHASHED	2	1.18E-06	0.052	y
sr-mmp_FEST	0.8558	128	3	sr-mmp_FEST	0.9103	UNHASHED	1	3.16E-07	0.0545	y
sr-mmp_RFC	0.8552	128	3	sr-mmp_RFC	0.9099	UNHASHED	1	3.00E-07	0.0547	y
sr-mmp_SVM	0.855	128	2	sr-mmp_SVM	0.9102	UNHASHED	1	2.33E-07	0.0552	y
nr-er_SVM	0.7407	128	3	nr-er_SVM	0.7962	UNHASHED	1	0.000570332	0.0555	y
cas-N6512_SVM	0.7407	128	3	cas-N6512_SVM	0.7962	UNHASHED	1	1.69E-11	0.0555	y
sr-mmp_SVM	0.855	128	2	sr-mmp_SVM	0.9114	UNHASHED	2	1.20E-07	0.0564	y
nr-er_SVM	0.7407	128	3	nr-er_SVM	0.7978	UNHASHED	2	0.000386994	0.0571	y
cas-N6512_SVM	0.7407	128	3	cas-N6512_SVM	0.7978	UNHASHED	2	4.53E-12	0.0571	y
nr-er_SVM	0.7407	128	3	nr-er_SVM	0.7982	UNHASHED	3	0.000350628	0.0575	y
cas-N6512_SVM	0.7407	128	3	cas-N6512_SVM	0.7982	UNHASHED	3	3.25E-12	0.0575	y
sr-mmp_SVM	0.855	128	2	sr-mmp_SVM	0.9129	UNHASHED	3	5.13E-08	0.0579	y
sr-mmp_SVM	0.8506	128	3	sr-mmp_SVM	0.9102	UNHASHED	1	3.10E-08	0.0596	y
sr-mmp_SVM	0.8506	128	3	sr-mmp_SVM	0.9114	UNHASHED	2	1.54E-08	0.0608	y
sr-mmp_SVM	0.8506	128	3	sr-mmp_SVM	0.9129	UNHASHED	3	6.32E-09	0.0623	y
cas-N6512_FEST	0.8741	128	1	cas-N6512_FEST	0.8864	4096	2	0.039491166	0.0123	y
cas-N6512_RFC	0.8679	128	1	cas-N6512_RFC	0.8808	4096	3	0.034980767	0.0129	y
cas-N6512_FEST	0.875	256	2	cas-N6512_FEST	0.888	4096	1	0.028697602	0.013	y
cas-N6512_FEST	0.8741	128	1	cas-N6512_FEST	0.888	4096	1	0.019559901	0.0139	y
cas-N6512_RFC	0.8679	128	1	cas-N6512_RFC	0.8823	4096	1	0.018235794	0.0144	y
cas-N6512_SVM	0.7565	128	1	cas-N6512_SVM	0.773	4096	2	0.04430082	0.0165	y
cas-N6512_RFC	0.8679	128	1	cas-N6512_RFC	0.8856	4096	2	0.003487478	0.0177	y
cas-N6512_SVM	0.7546	128	2	cas-N6512_SVM	0.773	4096	2	0.025150688	0.0184	y
cas-N6512_RFC	0.8617	128	2	cas-N6512_RFC	0.8808	4096	3	0.002036074	0.0191	y
cas-N6512_FEST	0.8618	128	2	cas-N6512_FEST	0.8811	4096	3	0.001810829	0.0193	y

cas-N6512_SVM	0.7407	128	3	cas-N6512_SVM	0.7607	4096	1	0.017338577	0.02	y
cas-N6512_SVM	0.7527	256	3	cas-N6512_SVM	0.773	4096	2	0.013655505	0.0203	y
cas-N6512_FEST	0.8607	256	3	cas-N6512_FEST	0.8811	4096	3	0.001000026	0.0204	y
cas-N6512_RFC	0.8617	128	2	cas-N6512_RFC	0.8823	4096	1	0.000847364	0.0206	y
cas-N6512_RFC	0.8602	256	3	cas-N6512_RFC	0.8808	4096	3	0.000907563	0.0206	y
sr-mmp_SVM	0.8682	128	1	sr-mmp_SVM	0.8896	4096	3	0.048194547	0.0214	y
sr-mmp_FEST	0.8657	128	2	sr-mmp_FEST	0.8872	4096	3	0.049009076	0.0215	y
cas-N6512_RFC	0.8602	256	3	cas-N6512_RFC	0.8823	4096	1	0.000357692	0.0221	y
sr-mmp_SVM	0.8655	256	3	sr-mmp_SVM	0.8877	4096	1	0.041980368	0.0222	y
sr-mmp_RFC	0.8645	128	2	sr-mmp_RFC	0.8869	4096	3	0.040764053	0.0224	y
sr-mmp_SVM	0.8696	256	2	sr-mmp_SVM	0.892	4096	2	0.037365883	0.0224	y
nr-ahr_SVM	0.884	256	3	nr-ahr_SVM	0.9067	4096	1	0.046000175	0.0227	y
nr-ahr_SVM	0.884	256	3	nr-ahr_SVM	0.907	4096	2	0.043069016	0.023	y
sr-mmp_SVM	0.8682	128	1	sr-mmp_SVM	0.892	4096	2	0.027336081	0.0238	y
cas-N6512_RFC	0.8617	128	2	cas-N6512_RFC	0.8856	4096	2	9.78E-05	0.0239	y
sr-mmp_RFC	0.8645	128	2	sr-mmp_RFC	0.8885	4096	2	0.027938762	0.024	y
sr-mmp_SVM	0.8655	256	3	sr-mmp_SVM	0.8896	4096	3	0.026751591	0.0241	y
nr-ahr_RFC	0.8825	128	3	nr-ahr_RFC	0.9067	4096	3	0.033913885	0.0242	y
cas-N6512_SVM	0.7407	128	3	cas-N6512_SVM	0.7649	4096	3	0.003857623	0.0242	y
sr-mmp_FEST	0.8558	128	3	sr-mmp_FEST	0.8801	4096	1	0.030346397	0.0243	y
cas-N6512_FEST	0.8618	128	2	cas-N6512_FEST	0.8864	4096	2	5.90E-05	0.0246	y
cas-N6512_RFC	0.8602	256	3	cas-N6512_RFC	0.8856	4096	2	3.66E-05	0.0254	y
nr-ahr_RFC	0.8825	128	3	nr-ahr_RFC	0.908	4096	1	0.025016185	0.0255	y
cas-N6512_FEST	0.8607	256	3	cas-N6512_FEST	0.8864	4096	2	2.83E-05	0.0257	y
nr-ahr_SVM	0.8787	128	3	nr-ahr_SVM	0.9047	4096	3	0.024240725	0.026	y
cas-N6512_FEST	0.8618	128	2	cas-N6512_FEST	0.888	4096	1	1.78E-05	0.0262	y
sr-mmp_SVM	0.8655	256	3	sr-mmp_SVM	0.892	4096	2	0.01444014	0.0265	y
cas-N6512_FEST	0.8607	256	3	cas-N6512_FEST	0.888	4096	1	8.18E-06	0.0273	y
nr-ahr_RFC	0.8825	128	3	nr-ahr_RFC	0.91	4096	2	0.01521409	0.0275	y
sr-mmp_RFC	0.8552	128	3	sr-mmp_RFC	0.883	4096	1	0.012886032	0.0278	y
nr-ahr_SVM	0.8787	128	3	nr-ahr_SVM	0.9067	4096	1	0.014827025	0.028	y
nr-ahr_SVM	0.8787	128	3	nr-ahr_SVM	0.907	4096	2	0.013731606	0.0283	y
nr-ahr_FEST	0.8791	128	3	nr-ahr_FEST	0.9077	4096	3	0.012566688	0.0286	y

sr-mmp_FEST	0.8558	128	3	sr-mmp_FEST	0.8846	4096	2	0.009725583	0.0288	y
nr-ahr_FEST	0.8791	128	3	nr-ahr_FEST	0.9083	4096	1	0.010730041	0.0292	y
nr-ahr_FEST	0.8791	128	3	nr-ahr_FEST	0.9104	4096	2	0.006014501	0.0313	y
sr-mmp_FEST	0.8558	128	3	sr-mmp_FEST	0.8872	4096	3	0.004638939	0.0314	y
sr-mmp_RFC	0.8552	128	3	sr-mmp_RFC	0.8869	4096	3	0.004315385	0.0317	y
nr-er_SVM	0.7407	128	3	nr-er_SVM	0.773	4096	2	0.048398169	0.0323	y
cas-N6512_SVM	0.7407	128	3	cas-N6512_SVM	0.773	4096	2	0.000102821	0.0323	y
sr-mmp_SVM	0.855	128	2	sr-mmp_SVM	0.8877	4096	1	0.003206145	0.0327	y
sr-mmp_RFC	0.8552	128	3	sr-mmp_RFC	0.8885	4096	2	0.002644634	0.0333	y
sr-mmp_SVM	0.855	128	2	sr-mmp_SVM	0.8896	4096	3	0.001756785	0.0346	y
cas-N6512_FEST	0.8456	128	3	cas-N6512_FEST	0.8811	4096	3	2.88E-08	0.0355	y
cas-N6512_RFC	0.8452	128	3	cas-N6512_RFC	0.8808	4096	3	2.74E-08	0.0356	y
sr-mmp_SVM	0.855	128	2	sr-mmp_SVM	0.892	4096	2	0.000781348	0.037	y
sr-mmp_SVM	0.8506	128	3	sr-mmp_SVM	0.8877	4096	1	0.000893214	0.0371	y
cas-N6512_RFC	0.8452	128	3	cas-N6512_RFC	0.8823	4096	1	6.66E-09	0.0371	y
sr-mmp_SVM	0.8506	128	3	sr-mmp_SVM	0.8896	4096	3	0.000459483	0.039	y
cas-N6512_RFC	0.8452	128	3	cas-N6512_RFC	0.8856	4096	2	2.48E-10	0.0404	y
cas-N6512_FEST	0.8456	128	3	cas-N6512_FEST	0.8864	4096	2	1.55E-10	0.0408	y
sr-mmp_SVM	0.8506	128	3	sr-mmp_SVM	0.892	4096	2	0.000188566	0.0414	y
cas-N6512_FEST	0.8456	128	3	cas-N6512_FEST	0.888	4096	1	2.85E-11	0.0424	y
cas-N6512_RFC	0.8679	128	1	cas-N6512_RFC	0.8811	2048	3	0.030857841	0.0132	y
cas-N6512_FEST	0.8741	128	1	cas-N6512_FEST	0.8876	2048	1	0.023476226	0.0135	y
cas-N6512_RFC	0.8679	128	1	cas-N6512_RFC	0.8836	2048	1	0.009861934	0.0157	y
cas-N6512_RFC	0.8679	128	1	cas-N6512_RFC	0.8852	2048	2	0.004333338	0.0173	y
cas-N6512_SVM	0.7546	128	2	cas-N6512_SVM	0.7725	2048	2	0.029463158	0.0179	y
cas-N6512_FEST	0.8618	128	2	cas-N6512_FEST	0.8799	2048	3	0.003512805	0.0181	y
cas-N6512_RFC	0.8617	128	2	cas-N6512_RFC	0.8811	2048	3	0.001717438	0.0194	y
cas-N6512_SVM	0.7407	128	3	cas-N6512_SVM	0.7616	2048	1	0.012830521	0.0209	y
cas-N6512_RFC	0.8617	128	2	cas-N6512_RFC	0.8836	2048	1	0.000376088	0.0219	y
cas-N6512_FEST	0.8618	128	2	cas-N6512_FEST	0.8852	2048	2	0.000137675	0.0234	y
cas-N6512_RFC	0.8617	128	2	cas-N6512_RFC	0.8852	2048	2	0.000129275	0.0235	y
nr-ahr_SVM	0.8787	128	3	nr-ahr_SVM	0.9023	2048	3	0.041806698	0.0236	y
nr-ahr_RFC	0.8825	128	3	nr-ahr_RFC	0.9061	2048	3	0.038836324	0.0236	y

sr-mmp_FEST	0.8558	128	3	sr-mmp_FEST	0.8807	2048	1	0.026340863	0.0249	y
cas-N6512_FEST	0.8618	128	2	cas-N6512_FEST	0.8876	2048	1	2.42E-05	0.0258	y
nr-ahr_RFC	0.8825	128	3	nr-ahr_RFC	0.9083	2048	1	0.023270858	0.0258	y
cas-N6512_SVM	0.7407	128	3	cas-N6512_SVM	0.7673	2048	3	0.001458622	0.0266	y
sr-mmp_FEST	0.8558	128	3	sr-mmp_FEST	0.8825	2048	3	0.016909607	0.0267	y
sr-mmp_RFC	0.8552	128	3	sr-mmp_RFC	0.882	2048	1	0.01668623	0.0268	y
nr-ahr_RFC	0.8825	128	3	nr-ahr_RFC	0.9096	2048	2	0.016853499	0.0271	y
sr-mmp_FEST	0.8558	128	3	sr-mmp_FEST	0.8832	2048	2	0.014123923	0.0274	y
nr-ahr_FEST	0.8791	128	3	nr-ahr_FEST	0.9068	2048	3	0.015831787	0.0277	y
sr-mmp_SVM	0.855	128	2	sr-mmp_SVM	0.8838	2048	1	0.009909394	0.0288	y
sr-mmp_RFC	0.8552	128	3	sr-mmp_RFC	0.8841	2048	3	0.009598423	0.0289	y
nr-ahr_SVM	0.8787	128	3	nr-ahr_SVM	0.9077	2048	1	0.011444425	0.029	y
nr-ahr_SVM	0.8787	128	3	nr-ahr_SVM	0.908	2048	2	0.010570581	0.0293	y
nr-ahr_FEST	0.8791	128	3	nr-ahr_FEST	0.9088	2048	1	0.009382936	0.0297	y
nr-ahr_FEST	0.8791	128	3	nr-ahr_FEST	0.9095	2048	2	0.007746489	0.0304	y
sr-mmp_SVM	0.855	128	2	sr-mmp_SVM	0.8857	2048	3	0.005820027	0.0307	y
sr-mmp_RFC	0.8552	128	3	sr-mmp_RFC	0.8859	2048	2	0.005789172	0.0307	y
cas-N6512_SVM	0.7407	128	3	cas-N6512_SVM	0.7725	2048	2	0.000132316	0.0318	y
sr-mmp_SVM	0.855	128	2	sr-mmp_SVM	0.888	2048	2	0.002922336	0.033	y
sr-mmp_SVM	0.8506	128	3	sr-mmp_SVM	0.8838	2048	1	0.003136527	0.0332	y
cas-N6512_FEST	0.8456	128	3	cas-N6512_FEST	0.8799	2048	3	8.59E-08	0.0343	y
sr-mmp_SVM	0.8506	128	3	sr-mmp_SVM	0.8857	2048	3	0.001731734	0.0351	y
cas-N6512_RFC	0.8452	128	3	cas-N6512_RFC	0.8811	2048	3	2.07E-08	0.0359	y
sr-mmp_SVM	0.8506	128	3	sr-mmp_SVM	0.888	2048	2	0.0008061	0.0374	y
cas-N6512_RFC	0.8452	128	3	cas-N6512_RFC	0.8836	2048	1	1.87E-09	0.0384	y
cas-N6512_FEST	0.8456	128	3	cas-N6512_FEST	0.8852	2048	2	5.32E-10	0.0396	y
cas-N6512_RFC	0.8452	128	3	cas-N6512_RFC	0.8852	2048	2	3.74E-10	0.04	y
cas-N6512_FEST	0.8456	128	3	cas-N6512_FEST	0.8876	2048	1	4.37E-11	0.042	y
sr-mmp_SVM	0.892	4096	2	sr-mmp_SVM	0.8707	512	3	0.047346288	-0.0213	y
cas-N6512_FEST	0.888	4096	1	cas-N6512_FEST	0.8707	512	3	0.003908171	-0.0173	y
cas-N6512_FEST	0.8878	1024	1	cas-N6512_FEST	0.8707	512	3	0.004359022	-0.0171	y
cas-N6512_FEST	0.8876	2048	1	cas-N6512_FEST	0.8707	512	3	0.004856086	-0.0169	y
cas-N6512_RFC	0.8856	4096	2	cas-N6512_RFC	0.8697	512	3	0.008449538	-0.0159	y

cas-N6512_FEST	0.8864	4096	2	cas-N6512_FEST	0.8707	512	3	0.009055037	-0.0157	y
cas-N6512_RFC	0.8852	2048	2	cas-N6512_RFC	0.8697	512	3	0.010311349	-0.0155	y
cas-N6512_FEST	0.886	512	1	cas-N6512_FEST	0.8707	512	3	0.011041558	-0.0153	y
cas-N6512_FEST	0.8852	1024	2	cas-N6512_FEST	0.8707	512	3	0.016192427	-0.0145	y
cas-N6512_FEST	0.8852	2048	2	cas-N6512_FEST	0.8707	512	3	0.016192427	-0.0145	y
cas-N6512_RFC	0.8836	2048	1	cas-N6512_RFC	0.8697	512	3	0.021850856	-0.0139	y
cas-N6512_RFC	0.8834	1024	2	cas-N6512_RFC	0.8697	512	3	0.023880592	-0.0137	y
cas-N6512_FEST	0.8838	256	1	cas-N6512_FEST	0.8707	512	3	0.030296498	-0.0131	y
cas-N6512_RFC	0.8823	4096	1	cas-N6512_RFC	0.8697	512	3	0.038166441	-0.0126	y
cas-N6512_RFC	0.8822	1024	1	cas-N6512_RFC	0.8697	512	3	0.039763543	-0.0125	y
cas-N6512_FEST	0.8741	128	1	cas-N6512_FEST	0.886	512	1	0.046545547	0.0119	y
cas-N6512_RFC	0.8617	128	2	cas-N6512_RFC	0.8787	512	2	0.00624435	0.017	y
cas-N6512_RFC	0.8617	128	2	cas-N6512_RFC	0.8798	512	1	0.003525382	0.0181	y
cas-N6512_RFC	0.8602	256	3	cas-N6512_RFC	0.8787	512	2	0.003001379	0.0185	y
cas-N6512_FEST	0.8618	128	2	cas-N6512_FEST	0.8804	512	2	0.002678468	0.0186	y
cas-N6512_SVM	0.7407	128	3	cas-N6512_SVM	0.7601	512	3	0.021061257	0.0194	y
cas-N6512_SVM	0.7407	128	3	cas-N6512_SVM	0.7602	512	1	0.020396493	0.0195	y
cas-N6512_RFC	0.8602	256	3	cas-N6512_RFC	0.8798	512	1	0.001629096	0.0196	y
cas-N6512_FEST	0.8607	256	3	cas-N6512_FEST	0.8804	512	2	0.001506901	0.0197	y
sr-mmp_FEST	0.8558	128	3	sr-mmp_FEST	0.8781	512	1	0.047592596	0.0223	y
sr-mmp_RFC	0.8552	128	3	sr-mmp_RFC	0.878	512	3	0.043040291	0.0228	y
nr-ahr_RFC	0.8825	128	3	nr-ahr_RFC	0.9054	512	1	0.04531395	0.0229	y
sr-mmp_FEST	0.8558	128	3	sr-mmp_FEST	0.8787	512	2	0.041727951	0.0229	y
nr-ahr_RFC	0.8825	128	3	nr-ahr_RFC	0.906	512	2	0.039711724	0.0235	y
cas-N6512_FEST	0.8618	128	2	cas-N6512_FEST	0.886	512	1	7.86E-05	0.0242	y
cas-N6512_SVM	0.7407	128	3	cas-N6512_SVM	0.765	512	2	0.00371063	0.0243	y
cas-N6512_RFC	0.8452	128	3	cas-N6512_RFC	0.8697	512	3	0.000167008	0.0245	y
sr-mmp_RFC	0.8552	128	3	sr-mmp_RFC	0.8799	512	1	0.027909863	0.0247	y
cas-N6512_FEST	0.8456	128	3	cas-N6512_FEST	0.8707	512	3	0.000110791	0.0251	y
cas-N6512_FEST	0.8607	256	3	cas-N6512_FEST	0.886	512	1	3.81E-05	0.0253	y
sr-mmp_SVM	0.8506	128	3	sr-mmp_SVM	0.876	512	2	0.025590758	0.0254	y
sr-mmp_RFC	0.8552	128	3	sr-mmp_RFC	0.8809	512	2	0.021949978	0.0257	y
nr-ahr_FEST	0.8791	128	3	nr-ahr_FEST	0.9057	512	2	0.020793327	0.0266	y

nr-ahr_FEST	0.8791	128	3	nr-ahr_FEST	0.9058	512	1	0.020293173	0.0267	y
sr-mmp_SVM	0.855	128	2	sr-mmp_SVM	0.8834	512	1	0.011038109	0.0284	y
sr-mmp_SVM	0.8506	128	3	sr-mmp_SVM	0.8834	512	1	0.003539213	0.0328	y
cas-N6512_RFC	0.8452	128	3	cas-N6512_RFC	0.8787	512	2	1.82E-07	0.0335	y
cas-N6512_RFC	0.8452	128	3	cas-N6512_RFC	0.8798	512	1	6.84E-08	0.0346	y
cas-N6512_FEST	0.8456	128	3	cas-N6512_FEST	0.8804	512	2	5.47E-08	0.0348	y
cas-N6512_FEST	0.8456	128	3	cas-N6512_FEST	0.886	512	1	2.34E-10	0.0404	y
cas-N6512_FEST	0.8878	1024	1	cas-N6512_FEST	0.8607	256	3	9.59E-06	-0.0271	y
cas-N6512_FEST	0.8876	2048	1	cas-N6512_FEST	0.8607	256	3	1.12E-05	-0.0269	y
cas-N6512_RFC	0.8852	2048	2	cas-N6512_RFC	0.8602	256	3	4.91E-05	-0.025	y
cas-N6512_FEST	0.8852	1024	2	cas-N6512_FEST	0.8607	256	3	6.81E-05	-0.0245	y
cas-N6512_FEST	0.8852	2048	2	cas-N6512_FEST	0.8607	256	3	6.81E-05	-0.0245	y
nr-ahr_SVM	0.908	2048	2	nr-ahr_SVM	0.884	256	3	0.034389869	-0.024	y
nr-ahr_SVM	0.9077	2048	1	nr-ahr_SVM	0.884	256	3	0.036825116	-0.0237	y
cas-N6512_RFC	0.8836	2048	1	cas-N6512_RFC	0.8602	256	3	0.000151409	-0.0234	y
cas-N6512_RFC	0.8834	1024	2	cas-N6512_RFC	0.8602	256	3	0.000173375	-0.0232	y
cas-N6512_FEST	0.8838	256	1	cas-N6512_FEST	0.8607	256	3	0.000180201	-0.0231	y
sr-mmp_SVM	0.888	2048	2	sr-mmp_SVM	0.8655	256	3	0.0391817	-0.0225	y
cas-N6512_RFC	0.8822	1024	1	cas-N6512_RFC	0.8602	256	3	0.000381368	-0.022	y
cas-N6512_RFC	0.8811	2048	3	cas-N6512_RFC	0.8602	256	3	0.000757266	-0.0209	y
cas-N6512_SVM	0.7725	2048	2	cas-N6512_SVM	0.7527	256	3	0.016201175	-0.0198	y
cas-N6512_FEST	0.8799	2048	3	cas-N6512_FEST	0.8607	256	3	0.00200258	-0.0192	y
cas-N6512_RFC	0.8776	256	1	cas-N6512_RFC	0.8602	256	3	0.005347539	-0.0174	y
cas-N6512_FEST	0.8775	1024	3	cas-N6512_FEST	0.8607	256	3	0.007118129	-0.0168	y
cas-N6512_SVM	0.7694	1024	2	cas-N6512_SVM	0.7527	256	3	0.043155951	-0.0167	y
cas-N6512_RFC	0.8766	1024	3	cas-N6512_RFC	0.8602	256	3	0.008785806	-0.0164	y
cas-N6512_FEST	0.875	256	2	cas-N6512_FEST	0.8607	256	3	0.022608135	-0.0143	y
cas-N6512_RFC	0.8742	256	2	cas-N6512_RFC	0.8602	256	3	0.025962735	-0.014	y
cas-N6512_FEST	0.8741	128	1	cas-N6512_FEST	0.8607	256	3	0.032932942	-0.0134	y
cas-N6512_FEST	0.8878	1024	1	cas-N6512_FEST	0.875	256	2	0.031314621	-0.0128	y
cas-N6512_FEST	0.8876	2048	1	cas-N6512_FEST	0.875	256	2	0.034131289	-0.0126	y
cas-N6512_RFC	0.8617	128	2	cas-N6512_RFC	0.8742	256	2	0.046185069	0.0125	y
cas-N6512_FEST	0.8618	128	2	cas-N6512_FEST	0.875	256	2	0.034952912	0.0132	y

cas-N6512_RFC	0.8452	128	3	cas-N6512_RFC	0.8602	256	3	0.023326441	0.015	y
cas-N6512_FEST	0.8456	128	3	cas-N6512_FEST	0.8607	256	3	0.022221397	0.0151	y
cas-N6512_RFC	0.8617	128	2	cas-N6512_RFC	0.8776	256	1	0.010700123	0.0159	y
cas-N6512_SVM	0.7407	128	3	cas-N6512_SVM	0.7626	256	1	0.009060959	0.0219	y
cas-N6512_FEST	0.8618	128	2	cas-N6512_FEST	0.8838	256	1	0.000350723	0.022	y
nr-ahr_FEST	0.8791	128	3	nr-ahr_FEST	0.9022	256	1	0.046219762	0.0231	y
sr-mmp_RFC	0.8552	128	3	sr-mmp_RFC	0.8784	256	1	0.039387369	0.0232	y
cas-N6512_SVM	0.7407	128	3	cas-N6512_SVM	0.7648	256	2	0.004009859	0.0241	y
sr-mmp_SVM	0.855	128	2	sr-mmp_SVM	0.8793	256	1	0.030753384	0.0243	y
sr-mmp_SVM	0.8506	128	3	sr-mmp_SVM	0.8793	256	1	0.01123287	0.0287	y
cas-N6512_RFC	0.8452	128	3	cas-N6512_RFC	0.8742	256	2	7.18E-06	0.029	y
cas-N6512_FEST	0.8456	128	3	cas-N6512_FEST	0.875	256	2	5.14E-06	0.0294	y
cas-N6512_RFC	0.8452	128	3	cas-N6512_RFC	0.8776	256	1	4.68E-07	0.0324	y
cas-N6512_FEST	0.8456	128	3	cas-N6512_FEST	0.8838	256	1	2.17E-09	0.0382	y
cas-N6512_FEST	0.8878	1024	1	cas-N6512_FEST	0.8456	128	3	3.53E-11	-0.0422	y
cas-N6512_FEST	0.8852	1024	2	cas-N6512_FEST	0.8456	128	3	5.32E-10	-0.0396	y
cas-N6512_RFC	0.8834	1024	2	cas-N6512_RFC	0.8452	128	3	2.28E-09	-0.0382	y
cas-N6512_RFC	0.8822	1024	1	cas-N6512_RFC	0.8452	128	3	7.33E-09	-0.037	y
sr-mmp_SVM	0.8857	1024	1	sr-mmp_SVM	0.8506	128	3	0.001731734	-0.0351	y
sr-mmp_SVM	0.8833	1024	2	sr-mmp_SVM	0.8506	128	3	0.00364688	-0.0327	y
cas-N6512_FEST	0.8775	1024	3	cas-N6512_FEST	0.8456	128	3	6.88E-07	-0.0319	y
cas-N6512_RFC	0.8766	1024	3	cas-N6512_RFC	0.8452	128	3	1.08E-06	-0.0314	y
sr-mmp_SVM	0.8857	1024	1	sr-mmp_SVM	0.855	128	2	0.005820027	-0.0307	y
sr-mmp_SVM	0.8806	1024	3	sr-mmp_SVM	0.8506	128	3	0.007915416	-0.03	y
nr-ahr_FEST	0.9086	1024	2	nr-ahr_FEST	0.8791	128	3	0.009902895	-0.0295	y
sr-mmp_RFC	0.8841	1024	2	sr-mmp_RFC	0.8552	128	3	0.009598423	-0.0289	y
sr-mmp_RFC	0.8841	1024	3	sr-mmp_RFC	0.8552	128	3	0.009598423	-0.0289	y
cas-N6512_SVM	0.7694	1024	2	cas-N6512_SVM	0.7407	128	3	0.000580965	-0.0287	y
cas-N6512_FEST	0.8741	128	1	cas-N6512_FEST	0.8456	128	3	1.02E-05	-0.0285	y
sr-mmp_SVM	0.8833	1024	2	sr-mmp_SVM	0.855	128	2	0.011337292	-0.0283	y
sr-mmp_RFC	0.8826	1024	1	sr-mmp_RFC	0.8552	128	3	0.014304558	-0.0274	y
sr-mmp_FEST	0.8828	1024	2	sr-mmp_FEST	0.8558	128	3	0.015662407	-0.027	y
nr-ahr_SVM	0.905	1024	1	nr-ahr_SVM	0.8787	128	3	0.022567224	-0.0263	y

nr-ahr_FEST	0.9052	1024	1	nr-ahr_FEST	0.8791	128	3	0.023454823	-0.0261	y
cas-N6512_FEST	0.8878	1024	1	cas-N6512_FEST	0.8618	128	2	2.08E-05	-0.026	y
sr-mmp_SVM	0.8806	1024	3	sr-mmp_SVM	0.855	128	2	0.022575089	-0.0256	y
nr-ahr_RFC	0.9076	1024	2	nr-ahr_RFC	0.8825	128	3	0.027514711	-0.0251	y
nr-ahr_RFC	0.9074	1024	1	nr-ahr_RFC	0.8825	128	3	0.028840922	-0.0249	y
sr-mmp_FEST	0.8806	1024	3	sr-mmp_FEST	0.8558	128	3	0.026975446	-0.0248	y
nr-ahr_SVM	0.9031	1024	2	nr-ahr_SVM	0.8787	128	3	0.035047587	-0.0244	y
nr-ahr_FEST	0.9029	1024	3	nr-ahr_FEST	0.8791	128	3	0.039718412	-0.0238	y
cas-N6512_FEST	0.8852	1024	2	cas-N6512_FEST	0.8618	128	2	0.000137675	-0.0234	y
cas-N6512_RFC	0.8679	128	1	cas-N6512_RFC	0.8452	128	3	0.000506698	-0.0227	y
cas-N6512_RFC	0.8834	1024	2	cas-N6512_RFC	0.8617	128	2	0.000427521	-0.0217	y
cas-N6512_SVM	0.7616	1024	1	cas-N6512_SVM	0.7407	128	3	0.012830521	-0.0209	y
cas-N6512_RFC	0.8822	1024	1	cas-N6512_RFC	0.8617	128	2	0.000900169	-0.0205	y
cas-N6512_SVM	0.7609	1024	3	cas-N6512_SVM	0.7407	128	3	0.016232157	-0.0202	y
cas-N6512_RFC	0.8617	128	2	cas-N6512_RFC	0.8452	128	3	0.012380683	-0.0165	y
cas-N6512_FEST	0.8618	128	2	cas-N6512_FEST	0.8456	128	3	0.013984834	-0.0162	y
cas-N6512_FEST	0.8775	1024	3	cas-N6512_FEST	0.8618	128	2	0.011727839	-0.0157	y
cas-N6512_RFC	0.8834	1024	2	cas-N6512_RFC	0.8679	128	1	0.010873579	-0.0155	y
cas-N6512_RFC	0.8766	1024	3	cas-N6512_RFC	0.8617	128	2	0.016973962	-0.0149	y
cas-N6512_RFC	0.8822	1024	1	cas-N6512_RFC	0.8679	128	1	0.019081595	-0.0143	y
cas-N6512_FEST	0.8878	1024	1	cas-N6512_FEST	0.8741	128	1	0.021441156	-0.0137	y
cas-N6512_FEST	0.8741	128	1	cas-N6512_FEST	0.8618	128	2	0.049786413	-0.0123	y
cas-N6512_FEST	0.8936	UNHASHED	1	cas-N6512_FEST	0.8876	UNHASHED	3	0.293394436	-0.006	
nr-ahr_RFC	0.9209	UNHASHED	1	nr-ahr_RFC	0.9149	UNHASHED	3	0.558355104	-0.006	
nr-er_FEST	0.7965	UNHASHED	1	nr-er_FEST	0.7913	UNHASHED	3	0.738784009	-0.0052	
nr-er_FEST	0.7963	UNHASHED	2	nr-er_FEST	0.7913	UNHASHED	3	0.74852249	-0.005	
sr-mmp_FEST	0.9103	UNHASHED	1	sr-mmp_FEST	0.9057	UNHASHED	3	0.633192769	-0.0046	
nr-ahr_RFC	0.9209	UNHASHED	1	nr-ahr_RFC	0.9167	UNHASHED	2	0.680570839	-0.0042	
cas-N6512_RFC	0.892	UNHASHED	1	cas-N6512_RFC	0.8882	UNHASHED	3	0.506739415	-0.0038	
sr-mmp_RFC	0.9099	UNHASHED	1	sr-mmp_RFC	0.9061	UNHASHED	3	0.693407451	-0.0038	
cas-N6512_FEST	0.8936	UNHASHED	1	cas-N6512_FEST	0.8899	UNHASHED	2	0.514808875	-0.0037	
sr-mmp_RFC	0.9099	UNHASHED	1	sr-mmp_RFC	0.9069	UNHASHED	2	0.755169617	-0.003	
nr-ahr_FEST	0.9169	UNHASHED	1	nr-ahr_FEST	0.9139	UNHASHED	3	0.772745224	-0.003	

cas-N6512_RFC	0.8908	UNHASHED	2	cas-N6512_RFC	0.8882	UNHASHED	3	0.650529834	-0.0026
sr-mmp_FEST	0.9103	UNHASHED	1	sr-mmp_FEST	0.9078	UNHASHED	2	0.794346809	-0.0025
cas-N6512_FEST	0.8899	UNHASHED	2	cas-N6512_FEST	0.8876	UNHASHED	3	0.689598545	-0.0023
nr-ahr_FEST	0.9162	UNHASHED	2	nr-ahr_FEST	0.9139	UNHASHED	3	0.825092826	-0.0023
sr-mmp_FEST	0.9078	UNHASHED	2	sr-mmp_FEST	0.9057	UNHASHED	3	0.82853616	-0.0021
nr-ahr_RFC	0.9167	UNHASHED	2	nr-ahr_RFC	0.9149	UNHASHED	3	0.862155907	-0.0018
nr-ahr_SVM	0.9128	UNHASHED	1	nr-ahr_SVM	0.9112	UNHASHED	2	0.879674769	-0.0016
cas-N6512_RFC	0.892	UNHASHED	1	cas-N6512_RFC	0.8908	UNHASHED	2	0.832955767	-0.0012
sr-mmp_RFC	0.9069	UNHASHED	2	sr-mmp_RFC	0.9061	UNHASHED	3	0.934321456	-0.0008
nr-ahr_FEST	0.9169	UNHASHED	1	nr-ahr_FEST	0.9162	UNHASHED	2	0.94595389	-0.0007
cas-N6512_FEST	0.888	4096	1	cas-N6512_FEST	0.8876	UNHASHED	3	0.944857764	-0.0004
nr-er_RFC	0.7974	UNHASHED	2	nr-er_RFC	0.7971	UNHASHED	3	0.984570568	-0.0003
cas-N6512_FEST	0.8878	1024	1	cas-N6512_FEST	0.8876	UNHASHED	3	0.972424727	-0.0002
nr-er_FEST	0.7965	UNHASHED	1	nr-er_FEST	0.7963	UNHASHED	2	0.989727235	-0.0002
cas-N6512_FEST	0.8876	2048	1	cas-N6512_FEST	0.8876	UNHASHED	3	1	0
nr-er_RFC	0.7969	UNHASHED	1	nr-er_RFC	0.7971	UNHASHED	3	0.989717454	0.0002
cas-N6512_SVM	0.7978	UNHASHED	2	cas-N6512_SVM	0.7982	UNHASHED	3	0.958429236	0.0004
nr-er_SVM	0.7978	UNHASHED	2	nr-er_SVM	0.7982	UNHASHED	3	0.979403733	0.0004
nr-er_RFC	0.7969	UNHASHED	1	nr-er_RFC	0.7974	UNHASHED	2	0.974291198	0.0005
sr-mmp_SVM	0.9102	UNHASHED	1	sr-mmp_SVM	0.9114	UNHASHED	2	0.899578597	0.0012
cas-N6512_FEST	0.8864	4096	2	cas-N6512_FEST	0.8876	UNHASHED	3	0.836195294	0.0012
sr-mmp_SVM	0.9114	UNHASHED	2	sr-mmp_SVM	0.9129	UNHASHED	3	0.873821139	0.0015
cas-N6512_SVM	0.7962	UNHASHED	1	cas-N6512_SVM	0.7978	UNHASHED	2	0.835200594	0.0016
nr-er_SVM	0.7962	UNHASHED	1	nr-er_SVM	0.7978	UNHASHED	2	0.917882249	0.0016
cas-N6512_FEST	0.886	512	1	cas-N6512_FEST	0.8876	UNHASHED	3	0.782977956	0.0016
cas-N6512_FEST	0.888	4096	1	cas-N6512_FEST	0.8899	UNHASHED	2	0.741221668	0.0019
cas-N6512_SVM	0.7962	UNHASHED	1	cas-N6512_SVM	0.7982	UNHASHED	3	0.794739202	0.002
nr-er_SVM	0.7962	UNHASHED	1	nr-er_SVM	0.7982	UNHASHED	3	0.897422146	0.002
cas-N6512_FEST	0.8878	1024	1	cas-N6512_FEST	0.8899	UNHASHED	2	0.715235719	0.0021
cas-N6512_FEST	0.8876	2048	1	cas-N6512_FEST	0.8899	UNHASHED	2	0.689598545	0.0023
cas-N6512_FEST	0.8852	1024	2	cas-N6512_FEST	0.8876	UNHASHED	3	0.680021457	0.0024
cas-N6512_FEST	0.8852	2048	2	cas-N6512_FEST	0.8876	UNHASHED	3	0.680021457	0.0024
cas-N6512_RFC	0.8856	4096	2	cas-N6512_RFC	0.8882	UNHASHED	3	0.654305528	0.0026

sr-mmp_SVM	0.9102	UNHASHED	1	sr-mmp_SVM	0.9129	UNHASHED	3	0.775645425	0.0027
cas-N6512_RFC	0.8852	2048	2	cas-N6512_RFC	0.8882	UNHASHED	3	0.605697952	0.003
nr-ahr_SVM	0.908	2048	2	nr-ahr_SVM	0.9112	UNHASHED	2	0.764756619	0.0032
nr-ahr_SVM	0.9077	2048	1	nr-ahr_SVM	0.9112	UNHASHED	2	0.743622628	0.0035
cas-N6512_FEST	0.8864	4096	2	cas-N6512_FEST	0.8899	UNHASHED	2	0.544414544	0.0035
nr-ahr_FEST	0.9104	4096	2	nr-ahr_FEST	0.9139	UNHASHED	3	0.740341423	0.0035
cas-N6512_FEST	0.8838	256	1	cas-N6512_FEST	0.8876	UNHASHED	3	0.515032299	0.0038
cas-N6512_FEST	0.886	512	1	cas-N6512_FEST	0.8899	UNHASHED	2	0.499788297	0.0039
nr-ahr_SVM	0.907	4096	2	nr-ahr_SVM	0.9112	UNHASHED	2	0.695191995	0.0042
nr-ahr_FEST	0.9095	2048	2	nr-ahr_FEST	0.9139	UNHASHED	3	0.677632056	0.0044
nr-ahr_SVM	0.9067	4096	1	nr-ahr_SVM	0.9112	UNHASHED	2	0.674845075	0.0045
cas-N6512_RFC	0.8836	2048	1	cas-N6512_RFC	0.8882	UNHASHED	3	0.430246291	0.0046
cas-N6512_FEST	0.8852	1024	2	cas-N6512_FEST	0.8899	UNHASHED	2	0.416904812	0.0047
cas-N6512_FEST	0.8852	2048	2	cas-N6512_FEST	0.8899	UNHASHED	2	0.416904812	0.0047
cas-N6512_RFC	0.8834	1024	2	cas-N6512_RFC	0.8882	UNHASHED	3	0.410675569	0.0048
nr-ahr_SVM	0.908	2048	2	nr-ahr_SVM	0.9128	UNHASHED	1	0.652269909	0.0048
nr-ahr_RFC	0.91	4096	2	nr-ahr_RFC	0.9149	UNHASHED	3	0.642170101	0.0049
nr-ahr_FEST	0.9088	2048	1	nr-ahr_FEST	0.9139	UNHASHED	3	0.630512394	0.0051
nr-ahr_SVM	0.9077	2048	1	nr-ahr_SVM	0.9128	UNHASHED	1	0.63234517	0.0051
nr-ahr_SVM	0.9128	UNHASHED	1	nr-ahr_SVM	0.918	UNHASHED	3	0.616665786	0.0052
cas-N6512_RFC	0.8856	4096	2	cas-N6512_RFC	0.8908	UNHASHED	2	0.367709052	0.0052
nr-ahr_FEST	0.9086	1024	2	nr-ahr_FEST	0.9139	UNHASHED	3	0.617339043	0.0053
nr-ahr_RFC	0.9096	2048	2	nr-ahr_RFC	0.9149	UNHASHED	3	0.615595706	0.0053
cas-N6512_FEST	0.888	4096	1	cas-N6512_FEST	0.8936	UNHASHED	1	0.326319359	0.0056
cas-N6512_RFC	0.8852	2048	2	cas-N6512_RFC	0.8908	UNHASHED	2	0.332446181	0.0056
nr-ahr_FEST	0.9083	4096	1	nr-ahr_FEST	0.9139	UNHASHED	3	0.597831992	0.0056
cas-N6512_FEST	0.8878	1024	1	cas-N6512_FEST	0.8936	UNHASHED	1	0.309560312	0.0058
nr-ahr_SVM	0.907	4096	2	nr-ahr_SVM	0.9128	UNHASHED	1	0.587003815	0.0058
nr-ahr_FEST	0.9104	4096	2	nr-ahr_FEST	0.9162	UNHASHED	2	0.580690491	0.0058
cas-N6512_RFC	0.8823	4096	1	cas-N6512_RFC	0.8882	UNHASHED	3	0.313058559	0.0059
cas-N6512_RFC	0.8822	1024	1	cas-N6512_RFC	0.8882	UNHASHED	3	0.305032404	0.006
cas-N6512_FEST	0.8876	2048	1	cas-N6512_FEST	0.8936	UNHASHED	1	0.293394436	0.006
cas-N6512_FEST	0.8838	256	1	cas-N6512_FEST	0.8899	UNHASHED	2	0.293552171	0.0061

nr-ahr_SVM	0.9067	4096	1	nr-ahr_SVM	0.9128	UNHASHED	1	0.568088023	0.0061	
nr-ahr_SVM	0.905	1024	1	nr-ahr_SVM	0.9112	UNHASHED	2	0.564828778	0.0062	
nr-ahr_FEST	0.9077	4096	3	nr-ahr_FEST	0.9139	UNHASHED	3	0.559763831	0.0062	
cas-N6512_RFC	0.8856	4096	2	cas-N6512_RFC	0.892	UNHASHED	1	0.266294132	0.0064	
cas-N6512_FEST	0.8811	4096	3	cas-N6512_FEST	0.8876	UNHASHED	3	0.268251491	0.0065	
nr-ahr_FEST	0.9104	4096	2	nr-ahr_FEST	0.9169	UNHASHED	1	0.535158445	0.0065	
nr-ahr_SVM	0.9047	4096	3	nr-ahr_SVM	0.9112	UNHASHED	2	0.546430341	0.0065	
nr-ahr_RFC	0.9083	2048	1	nr-ahr_RFC	0.9149	UNHASHED	3	0.533108843	0.0066	
nr-ahr_RFC	0.91	4096	2	nr-ahr_RFC	0.9167	UNHASHED	2	0.523300615	0.0067	
nr-ahr_FEST	0.9095	2048	2	nr-ahr_FEST	0.9162	UNHASHED	2	0.524345686	0.0067	
nr-ahr_SVM	0.9112	UNHASHED	2	nr-ahr_SVM	0.918	UNHASHED	3	0.514444541	0.0068	
cas-N6512_RFC	0.8852	2048	2	cas-N6512_RFC	0.892	UNHASHED	1	0.23797788	0.0068	
nr-ahr_RFC	0.908	4096	1	nr-ahr_RFC	0.9149	UNHASHED	3	0.514968213	0.0069	
cas-N6512_RFC	0.8811	2048	3	cas-N6512_RFC	0.8882	UNHASHED	3	0.22594772	0.0071	
nr-ahr_RFC	0.9096	2048	2	nr-ahr_RFC	0.9167	UNHASHED	2	0.499233147	0.0071	
nr-ahr_FEST	0.9068	2048	3	nr-ahr_FEST	0.9139	UNHASHED	3	0.505153327	0.0071	
cas-N6512_FEST	0.8804	512	2	cas-N6512_FEST	0.8876	UNHASHED	3	0.220770119	0.0072	
cas-N6512_RFC	0.8836	2048	1	cas-N6512_RFC	0.8908	UNHASHED	2	0.214347953	0.0072	
cas-N6512_FEST	0.8864	4096	2	cas-N6512_FEST	0.8936	UNHASHED	1	0.208606956	0.0072	
nr-ahr_RFC	0.9076	1024	2	nr-ahr_RFC	0.9149	UNHASHED	3	0.491328184	0.0073	
nr-ahr_FEST	0.9088	2048	1	nr-ahr_FEST	0.9162	UNHASHED	2	0.482703564	0.0074	
cas-N6512_RFC	0.8808	4096	3	cas-N6512_RFC	0.8882	UNHASHED	3	0.207232707	0.0074	
cas-N6512_RFC	0.8834	1024	2	cas-N6512_RFC	0.8908	UNHASHED	2	0.202087623	0.0074	
nr-ahr_FEST	0.9095	2048	2	nr-ahr_FEST	0.9169	UNHASHED	1	0.481164253	0.0074	
nr-ahr_RFC	0.9074	1024	1	nr-ahr_RFC	0.9149	UNHASHED	3	0.479746859	0.0075	
nr-ahr_FEST	0.9086	1024	2	nr-ahr_FEST	0.9162	UNHASHED	2	0.471169542	0.0076	
cas-N6512_FEST	0.886	512	1	cas-N6512_FEST	0.8936	UNHASHED	1	0.18482055	0.0076	
cas-N6512_FEST	0.8799	2048	3	cas-N6512_FEST	0.8876	UNHASHED	3	0.190834003	0.0077	
nr-ahr_SVM	0.905	1024	1	nr-ahr_SVM	0.9128	UNHASHED	1	0.46720655	0.0078	
nr-ahr_FEST	0.9083	4096	1	nr-ahr_FEST	0.9162	UNHASHED	2	0.454177495	0.0079	
nr-ahr_FEST	0.9058	512	1	nr-ahr_FEST	0.9139	UNHASHED	3	0.448182513	0.0081	
nr-ahr_SVM	0.9031	1024	2	nr-ahr_SVM	0.9112	UNHASHED	2	0.453977145	0.0081	
nr-ahr_FEST	0.9088	2048	1	nr-ahr_FEST	0.9169	UNHASHED	1	0.441461537	0.0081	

nr-ahr_SVM	0.9047	4096	3	nr-ahr_SVM	0.9128	UNHASHED	1	0.450571474	0.0081	
nr-ahr_FEST	0.9057	512	2	nr-ahr_FEST	0.9139	UNHASHED	3	0.442707342	0.0082	
nr-ahr_RFC	0.9067	4096	3	nr-ahr_RFC	0.9149	UNHASHED	3	0.440491375	0.0082	
nr-ahr_FEST	0.9086	1024	2	nr-ahr_FEST	0.9169	UNHASHED	1	0.430496094	0.0083	
cas-N6512_RFC	0.8798	512	1	cas-N6512_RFC	0.8882	UNHASHED	3	0.153126667	0.0084	
cas-N6512_FEST	0.8852	1024	2	cas-N6512_FEST	0.8936	UNHASHED	1	0.143468826	0.0084	
cas-N6512_FEST	0.8852	2048	2	cas-N6512_FEST	0.8936	UNHASHED	1	0.143468826	0.0084	
cas-N6512_RFC	0.8836	2048	1	cas-N6512_RFC	0.892	UNHASHED	1	0.14635588	0.0084	
nr-ahr_RFC	0.9083	2048	1	nr-ahr_RFC	0.9167	UNHASHED	2	0.425540232	0.0084	
cas-N6512_RFC	0.8823	4096	1	cas-N6512_RFC	0.8908	UNHASHED	2	0.143822379	0.0085	
nr-ahr_FEST	0.9077	4096	3	nr-ahr_FEST	0.9162	UNHASHED	2	0.421321257	0.0085	
cas-N6512_RFC	0.8834	1024	2	cas-N6512_RFC	0.892	UNHASHED	1	0.137160653	0.0086	
cas-N6512_RFC	0.8822	1024	1	cas-N6512_RFC	0.8908	UNHASHED	2	0.139253692	0.0086	
nr-ahr_FEST	0.9083	4096	1	nr-ahr_FEST	0.9169	UNHASHED	1	0.414367097	0.0086	
nr-ahr_FEST	0.9052	1024	1	nr-ahr_FEST	0.9139	UNHASHED	3	0.415948353	0.0087	
nr-ahr_RFC	0.908	4096	1	nr-ahr_RFC	0.9167	UNHASHED	2	0.409543985	0.0087	
nr-ahr_RFC	0.9061	2048	3	nr-ahr_RFC	0.9149	UNHASHED	3	0.408456849	0.0088	
cas-N6512_FEST	0.8811	4096	3	cas-N6512_FEST	0.8899	UNHASHED	2	0.131976697	0.0088	
nr-ahr_SVM	0.9023	2048	3	nr-ahr_SVM	0.9112	UNHASHED	2	0.411503732	0.0089	
nr-ahr_RFC	0.906	512	2	nr-ahr_RFC	0.9149	UNHASHED	3	0.403264432	0.0089	
nr-ahr_RFC	0.9076	1024	2	nr-ahr_RFC	0.9167	UNHASHED	2	0.38881544	0.0091	
nr-ahr_FEST	0.9077	4096	3	nr-ahr_FEST	0.9169	UNHASHED	1	0.383267522	0.0092	
nr-ahr_RFC	0.9074	1024	1	nr-ahr_RFC	0.9167	UNHASHED	2	0.378709454	0.0093	
nr-ahr_FEST	0.9068	2048	3	nr-ahr_FEST	0.9162	UNHASHED	2	0.374902713	0.0094	
cas-N6512_RFC	0.8787	512	2	cas-N6512_RFC	0.8882	UNHASHED	3	0.106993127	0.0095	
cas-N6512_FEST	0.8804	512	2	cas-N6512_FEST	0.8899	UNHASHED	2	0.104437947	0.0095	
nr-ahr_RFC	0.9054	512	1	nr-ahr_RFC	0.9149	UNHASHED	3	0.372996893	0.0095	
nr-ahr_SVM	0.9031	1024	2	nr-ahr_SVM	0.9128	UNHASHED	1	0.368051359	0.0097	
nr-er_FEST	0.7816	4096	2	nr-er_FEST	0.7913	UNHASHED	3	0.53844625	0.0097	
cas-N6512_RFC	0.8811	2048	3	cas-N6512_RFC	0.8908	UNHASHED	2	0.09617484	0.0097	
cas-N6512_RFC	0.8823	4096	1	cas-N6512_RFC	0.892	UNHASHED	1	0.094425992	0.0097	
cas-N6512_FEST	0.8838	256	1	cas-N6512_FEST	0.8936	UNHASHED	1	0.088855954	0.0098	
cas-N6512_RFC	0.8822	1024	1	cas-N6512_RFC	0.892	UNHASHED	1	0.091148862	0.0098	

nr-ahr_SVM	0.9012	512	1	nr-ahr_SVM	0.9112	UNHASHED	2	0.3573384	0.01	
nr-ahr_SVM	0.908	2048	2	nr-ahr_SVM	0.918	UNHASHED	3	0.34157111	0.01	
nr-ahr_RFC	0.9067	4096	3	nr-ahr_RFC	0.9167	UNHASHED	2	0.344699513	0.01	
cas-N6512_FEST	0.8799	2048	3	cas-N6512_FEST	0.8899	UNHASHED	2	0.087762548	0.01	
cas-N6512_RFC	0.8808	4096	3	cas-N6512_RFC	0.8908	UNHASHED	2	0.086524922	0.01	
nr-ahr_FEST	0.9068	2048	3	nr-ahr_FEST	0.9169	UNHASHED	1	0.339536112	0.0101	
cas-N6512_FEST	0.8775	1024	3	cas-N6512_FEST	0.8876	UNHASHED	3	0.087801333	0.0101	
nr-ahr_SVM	0.9077	2048	1	nr-ahr_SVM	0.918	UNHASHED	3	0.327643264	0.0103	
nr-ahr_FEST	0.9058	512	1	nr-ahr_FEST	0.9162	UNHASHED	2	0.327404867	0.0104	
nr-ahr_SVM	0.9023	2048	3	nr-ahr_SVM	0.9128	UNHASHED	1	0.330767279	0.0105	
nr-ahr_FEST	0.9057	512	2	nr-ahr_FEST	0.9162	UNHASHED	2	0.322891424	0.0105	
nr-ahr_RFC	0.9061	2048	3	nr-ahr_RFC	0.9167	UNHASHED	2	0.317233223	0.0106	
cas-N6512_RFC	0.8776	256	1	cas-N6512_RFC	0.8882	UNHASHED	3	0.072766303	0.0106	
nr-ahr_RFC	0.906	512	2	nr-ahr_RFC	0.9167	UNHASHED	2	0.312806256	0.0107	
nr-ahr_RFC	0.91	4096	2	nr-ahr_RFC	0.9209	UNHASHED	1	0.29382241	0.0109	
cas-N6512_RFC	0.8811	2048	3	cas-N6512_RFC	0.892	UNHASHED	1	0.06086833	0.0109	
nr-ahr_SVM	0.907	4096	2	nr-ahr_SVM	0.918	UNHASHED	3	0.296671011	0.011	
nr-ahr_FEST	0.9052	1024	1	nr-ahr_FEST	0.9162	UNHASHED	2	0.300965637	0.011	
nr-ahr_FEST	0.9029	1024	3	nr-ahr_FEST	0.9139	UNHASHED	3	0.306311748	0.011	
cas-N6512_RFC	0.8798	512	1	cas-N6512_RFC	0.8908	UNHASHED	2	0.059926951	0.011	
nr-ahr_FEST	0.9058	512	1	nr-ahr_FEST	0.9169	UNHASHED	1	0.295052196	0.0111	
cas-N6512_RFC	0.8808	4096	3	cas-N6512_RFC	0.892	UNHASHED	1	0.05425748	0.0112	
nr-ahr_FEST	0.9057	512	2	nr-ahr_FEST	0.9169	UNHASHED	1	0.290839808	0.0112	
nr-ahr_SVM	0.9067	4096	1	nr-ahr_SVM	0.918	UNHASHED	3	0.28404661	0.0113	
nr-ahr_RFC	0.9054	512	1	nr-ahr_RFC	0.9167	UNHASHED	2	0.287143108	0.0113	
nr-ahr_RFC	0.9096	2048	2	nr-ahr_RFC	0.9209	UNHASHED	1	0.276953702	0.0113	
nr-ahr_SVM	0.9012	512	1	nr-ahr_SVM	0.9128	UNHASHED	1	0.283830429	0.0116	
cas-N6512_RFC	0.8766	1024	3	cas-N6512_RFC	0.8882	UNHASHED	3	0.05006353	0.0116	
nr-ahr_FEST	0.9022	256	1	nr-ahr_FEST	0.9139	UNHASHED	3	0.277334052	0.0117	
nr-ahr_FEST	0.9052	1024	1	nr-ahr_FEST	0.9169	UNHASHED	1	0.270414353	0.0117	
nr-er_FEST	0.7795	4096	3	nr-er_FEST	0.7913	UNHASHED	3	0.454917522	0.0118	
nr-ahr_SVM	0.8989	256	1	nr-ahr_SVM	0.9112	UNHASHED	2	0.26002699	0.0123	
nr-er_FEST	0.779	1024	2	nr-er_FEST	0.7913	UNHASHED	3	0.436195309	0.0123	

nr-er_FEST	0.779	2048	2	nr-er_FEST	0.7913	UNHASHED	3	0.436195309	0.0123
nr-ahr_RFC	0.9083	2048	1	nr-ahr_RFC	0.9209	UNHASHED	1	0.226938991	0.0126
nr-ahr_RFC	0.908	4096	1	nr-ahr_RFC	0.9209	UNHASHED	1	0.21641482	0.0129
nr-ahr_SVM	0.905	1024	1	nr-ahr_SVM	0.918	UNHASHED	3	0.219687674	0.013
nr-ahr_SVM	0.9047	4096	3	nr-ahr_SVM	0.918	UNHASHED	3	0.209560296	0.0133
nr-ahr_FEST	0.9029	1024	3	nr-ahr_FEST	0.9162	UNHASHED	2	0.213559878	0.0133
nr-ahr_RFC	0.9076	1024	2	nr-ahr_RFC	0.9209	UNHASHED	1	0.202957734	0.0133
nr-ahr_RFC	0.9014	256	1	nr-ahr_RFC	0.9149	UNHASHED	3	0.209783774	0.0135
nr-ahr_RFC	0.9074	1024	1	nr-ahr_RFC	0.9209	UNHASHED	1	0.196471655	0.0135
nr-ahr_RFC	0.9012	1024	3	nr-ahr_RFC	0.9149	UNHASHED	3	0.203318506	0.0137
nr-ahr_SVM	0.8989	256	1	nr-ahr_SVM	0.9128	UNHASHED	1	0.201390257	0.0139
nr-ahr_FEST	0.9022	256	1	nr-ahr_FEST	0.9162	UNHASHED	2	0.191160021	0.014
nr-ahr_FEST	0.9029	1024	3	nr-ahr_FEST	0.9169	UNHASHED	1	0.189667931	0.014
nr-ahr_RFC	0.9067	4096	3	nr-ahr_RFC	0.9209	UNHASHED	1	0.175010646	0.0142
nr-ahr_RFC	0.9005	256	2	nr-ahr_RFC	0.9149	UNHASHED	3	0.181874361	0.0144
nr-ahr_SVM	0.8967	512	2	nr-ahr_SVM	0.9112	UNHASHED	2	0.186379752	0.0145
nr-ahr_FEST	0.8993	256	2	nr-ahr_FEST	0.9139	UNHASHED	3	0.178096578	0.0146
nr-ahr_FEST	0.9022	256	1	nr-ahr_FEST	0.9169	UNHASHED	1	0.169170171	0.0147
nr-er_FEST	0.7816	4096	2	nr-er_FEST	0.7963	UNHASHED	2	0.349372095	0.0147
nr-ahr_RFC	0.9061	2048	3	nr-ahr_RFC	0.9209	UNHASHED	1	0.158101256	0.0148
nr-ahr_SVM	0.9031	1024	2	nr-ahr_SVM	0.918	UNHASHED	3	0.161405861	0.0149
nr-er_FEST	0.7816	4096	2	nr-er_FEST	0.7965	UNHASHED	1	0.342779922	0.0149
nr-ahr_RFC	0.906	512	2	nr-ahr_RFC	0.9209	UNHASHED	1	0.155411998	0.0149
nr-ahr_RFC	0.8997	512	3	nr-ahr_RFC	0.9149	UNHASHED	3	0.159545595	0.0152
nr-ahr_RFC	0.9014	256	1	nr-ahr_RFC	0.9167	UNHASHED	2	0.153401631	0.0153
nr-ahr_RFC	0.9012	1024	3	nr-ahr_RFC	0.9167	UNHASHED	2	0.148287228	0.0155
nr-ahr_RFC	0.9054	512	1	nr-ahr_RFC	0.9209	UNHASHED	1	0.14002485	0.0155
nr-ahr_SVM	0.9023	2048	3	nr-ahr_SVM	0.918	UNHASHED	3	0.1408174	0.0157
nr-ahr_SVM	0.8967	512	2	nr-ahr_SVM	0.9128	UNHASHED	1	0.140858216	0.0161
nr-ahr_RFC	0.9005	256	2	nr-ahr_RFC	0.9167	UNHASHED	2	0.131440807	0.0162
nr-er_FEST	0.775	2048	3	nr-er_FEST	0.7913	UNHASHED	3	0.303465487	0.0163
nr-ahr_RFC	0.8983	128	1	nr-ahr_RFC	0.9149	UNHASHED	3	0.125695991	0.0166
nr-ahr_FEST	0.8973	512	3	nr-ahr_FEST	0.9139	UNHASHED	3	0.127442299	0.0166

nr-ahr_SVM	0.9012	512	1	nr-ahr_SVM	0.918	UNHASHED	3	0.11598	0.0168	
nr-er_FEST	0.7795	4096	3	nr-er_FEST	0.7963	UNHASHED	2	0.28556183	0.0168	
nr-ahr_FEST	0.8993	256	2	nr-ahr_FEST	0.9162	UNHASHED	2	0.117023878	0.0169	
nr-ahr_RFC	0.8997	512	3	nr-ahr_RFC	0.9167	UNHASHED	2	0.114101985	0.017	
nr-er_FEST	0.7795	4096	3	nr-er_FEST	0.7965	UNHASHED	1	0.279791258	0.017	
nr-er_FEST	0.779	1024	2	nr-er_FEST	0.7963	UNHASHED	2	0.271634412	0.0173	
nr-er_FEST	0.779	2048	2	nr-er_FEST	0.7963	UNHASHED	2	0.271634412	0.0173	
nr-er_FEST	0.779	1024	2	nr-er_FEST	0.7965	UNHASHED	1	0.266057877	0.0175	
nr-er_FEST	0.779	2048	2	nr-er_FEST	0.7965	UNHASHED	1	0.266057877	0.0175	
nr-ahr_FEST	0.8993	256	2	nr-ahr_FEST	0.9169	UNHASHED	1	0.102031976	0.0176	
sr-mmp_RFC	0.8885	4096	2	sr-mmp_RFC	0.9061	UNHASHED	3	0.081568365	0.0176	
nr-ahr_SVM	0.8935	1024	3	nr-ahr_SVM	0.9112	UNHASHED	2	0.109181792	0.0177	
sr-mmp_SVM	0.892	4096	2	sr-mmp_SVM	0.9102	UNHASHED	1	0.067152736	0.0182	
nr-ahr_RFC	0.8983	128	1	nr-ahr_RFC	0.9167	UNHASHED	2	0.088253561	0.0184	
sr-mmp_RFC	0.8885	4096	2	sr-mmp_RFC	0.9069	UNHASHED	2	0.068156165	0.0184	
sr-mmp_FEST	0.8872	4096	3	sr-mmp_FEST	0.9057	UNHASHED	3	0.068099715	0.0185	
nr-ahr_SVM	0.8923	256	2	nr-ahr_SVM	0.9112	UNHASHED	2	0.087982544	0.0189	
nr-ahr_FEST	0.895	128	1	nr-ahr_FEST	0.9139	UNHASHED	3	0.084223179	0.0189	
nr-ahr_FEST	0.8973	512	3	nr-ahr_FEST	0.9162	UNHASHED	2	0.08099253	0.0189	
nr-er_FEST	0.7723	1024	3	nr-er_FEST	0.7913	UNHASHED	3	0.231179989	0.019	
nr-ahr_SVM	0.8989	256	1	nr-ahr_SVM	0.918	UNHASHED	3	0.075474447	0.0191	
sr-mmp_RFC	0.8869	4096	3	sr-mmp_RFC	0.9061	UNHASHED	3	0.05824456	0.0192	
nr-ahr_SVM	0.8935	1024	3	nr-ahr_SVM	0.9128	UNHASHED	1	0.079591499	0.0193	
sr-mmp_SVM	0.892	4096	2	sr-mmp_SVM	0.9114	UNHASHED	2	0.050387282	0.0194	
nr-ahr_RFC	0.9014	256	1	nr-ahr_RFC	0.9209	UNHASHED	1	0.065964676	0.0195	
nr-ahr_FEST	0.8973	512	3	nr-ahr_FEST	0.9169	UNHASHED	1	0.069889149	0.0196	
nr-ahr_RFC	0.9012	1024	3	nr-ahr_RFC	0.9209	UNHASHED	1	0.063362457	0.0197	
nr-er_RFC	0.7771	4096	3	nr-er_RFC	0.7969	UNHASHED	1	0.208721673	0.0198	
nr-er_RFC	0.777	1024	2	nr-er_RFC	0.7969	UNHASHED	1	0.206463403	0.0199	
nr-er_RFC	0.7771	4096	3	nr-er_RFC	0.7971	UNHASHED	3	0.204090954	0.02	
nr-er_RFC	0.777	1024	2	nr-er_RFC	0.7971	UNHASHED	3	0.201869245	0.0201	
nr-er_RFC	0.7771	4096	3	nr-er_RFC	0.7974	UNHASHED	2	0.197282992	0.0203	
nr-er_RFC	0.777	1024	2	nr-er_RFC	0.7974	UNHASHED	2	0.195115716	0.0204	

nr-ahr_RFC	0.9005	256	2	nr-ahr_RFC	0.9209	UNHASHED	1	0.054930026	0.0204
nr-ahr_SVM	0.8923	256	2	nr-ahr_SVM	0.9128	UNHASHED	1	0.063269408	0.0205
nr-ahr_FEST	0.895	128	1	nr-ahr_FEST	0.9162	UNHASHED	2	0.051497828	0.0212
nr-er_FEST	0.775	2048	3	nr-er_FEST	0.7963	UNHASHED	2	0.177093929	0.0213
nr-er_FEST	0.7698	512	2	nr-er_FEST	0.7913	UNHASHED	3	0.176166547	0.0215
nr-er_FEST	0.7698	512	3	nr-er_FEST	0.7913	UNHASHED	3	0.176166547	0.0215
nr-er_FEST	0.775	2048	3	nr-er_FEST	0.7965	UNHASHED	1	0.172996077	0.0215
nr-er_FEST	0.7697	4096	1	nr-er_FEST	0.7913	UNHASHED	3	0.174192152	0.0216
nr-er_SVM	0.773	4096	2	nr-er_SVM	0.7962	UNHASHED	1	0.142086472	0.0232
nr-er_RFC	0.7735	4096	2	nr-er_RFC	0.7969	UNHASHED	1	0.138337203	0.0234
nr-er_RFC	0.7735	4096	2	nr-er_RFC	0.7971	UNHASHED	3	0.134938444	0.0236
nr-er_SVM	0.7725	2048	2	nr-er_SVM	0.7962	UNHASHED	1	0.133820704	0.0237
nr-er_RFC	0.7735	4096	2	nr-er_RFC	0.7974	UNHASHED	2	0.129959752	0.0239
nr-er_FEST	0.7723	1024	3	nr-er_FEST	0.7963	UNHASHED	2	0.128990473	0.024
nr-er_FEST	0.7723	1024	3	nr-er_FEST	0.7965	UNHASHED	1	0.12577445	0.0242
nr-er_FEST	0.767	1024	1	nr-er_FEST	0.7913	UNHASHED	3	0.126992937	0.0243
nr-er_RFC	0.7725	2048	2	nr-er_RFC	0.7969	UNHASHED	1	0.122513056	0.0244
nr-er_FEST	0.7668	2048	1	nr-er_FEST	0.7913	UNHASHED	3	0.123942792	0.0245
nr-er_RFC	0.7725	2048	2	nr-er_RFC	0.7971	UNHASHED	3	0.119421116	0.0246
nr-er_RFC	0.7722	1024	3	nr-er_RFC	0.7969	UNHASHED	1	0.118055969	0.0247
nr-er_SVM	0.773	4096	2	nr-er_SVM	0.7978	UNHASHED	2	0.116126948	0.0248
nr-er_RFC	0.7722	1024	3	nr-er_RFC	0.7971	UNHASHED	3	0.115052757	0.0249
nr-er_RFC	0.7725	2048	2	nr-er_RFC	0.7974	UNHASHED	2	0.114896413	0.0249
nr-er_RFC	0.7722	1024	3	nr-er_RFC	0.7974	UNHASHED	2	0.11065922	0.0252
nr-er_SVM	0.773	4096	2	nr-er_SVM	0.7982	UNHASHED	3	0.110248752	0.0252
nr-er_SVM	0.7725	2048	2	nr-er_SVM	0.7978	UNHASHED	2	0.109071256	0.0253
nr-er_SVM	0.7725	2048	2	nr-er_SVM	0.7982	UNHASHED	3	0.103478543	0.0257
nr-er_RFC	0.7711	512	2	nr-er_RFC	0.7969	UNHASHED	1	0.102805559	0.0258
nr-er_FEST	0.7654	256	3	nr-er_FEST	0.7913	UNHASHED	3	0.104182201	0.0259
nr-er_RFC	0.7711	512	2	nr-er_RFC	0.7971	UNHASHED	3	0.100114228	0.026
nr-er_RFC	0.7711	512	2	nr-er_RFC	0.7974	UNHASHED	2	0.096181293	0.0263
nr-er_FEST	0.7698	512	2	nr-er_FEST	0.7963	UNHASHED	2	0.094235194	0.0265
nr-er_FEST	0.7698	512	3	nr-er_FEST	0.7963	UNHASHED	2	0.094235194	0.0265

nr-er_FEST	0.7647	512	1	nr-er_FEST	0.7913	UNHASHED	3	0.095297198	0.0266
nr-er_FEST	0.7697	4096	1	nr-er_FEST	0.7963	UNHASHED	2	0.093020901	0.0266
nr-er_FEST	0.7698	512	2	nr-er_FEST	0.7965	UNHASHED	1	0.091727508	0.0267
nr-er_FEST	0.7698	512	3	nr-er_FEST	0.7965	UNHASHED	1	0.091727508	0.0267
nr-er_FEST	0.7646	256	2	nr-er_FEST	0.7913	UNHASHED	3	0.094079531	0.0267
nr-er_FEST	0.7697	4096	1	nr-er_FEST	0.7965	UNHASHED	1	0.090539252	0.0268
nr-er_SVM	0.7694	1024	2	nr-er_SVM	0.7962	UNHASHED	1	0.090675969	0.0268
nr-er_RFC	0.77	512	3	nr-er_RFC	0.7969	UNHASHED	1	0.089182207	0.0269
nr-er_RFC	0.7699	2048	3	nr-er_RFC	0.7969	UNHASHED	1	0.088020293	0.027
nr-er_RFC	0.77	512	3	nr-er_RFC	0.7971	UNHASHED	3	0.086781251	0.0271
nr-er_RFC	0.7699	2048	3	nr-er_RFC	0.7971	UNHASHED	3	0.085644659	0.0272
nr-er_RFC	0.77	512	3	nr-er_RFC	0.7974	UNHASHED	2	0.083276512	0.0274
nr-er_FEST	0.7638	256	1	nr-er_FEST	0.7913	UNHASHED	3	0.084785968	0.0275
nr-er_RFC	0.7699	2048	3	nr-er_RFC	0.7974	UNHASHED	2	0.082177231	0.0275
nr-er_SVM	0.7694	1024	2	nr-er_SVM	0.7978	UNHASHED	2	0.072647218	0.0284
nr-er_SVM	0.7694	1024	2	nr-er_SVM	0.7982	UNHASHED	3	0.068624638	0.0288
nr-er_SVM	0.7673	2048	3	nr-er_SVM	0.7962	UNHASHED	1	0.068464429	0.0289
nr-er_FEST	0.767	1024	1	nr-er_FEST	0.7963	UNHASHED	2	0.064755852	0.0293
nr-er_FEST	0.7668	2048	1	nr-er_FEST	0.7963	UNHASHED	2	0.062983323	0.0295
nr-er_FEST	0.767	1024	1	nr-er_FEST	0.7965	UNHASHED	1	0.062909716	0.0295
nr-er_FEST	0.7668	2048	1	nr-er_FEST	0.7965	UNHASHED	1	0.061179139	0.0297
nr-er_SVM	0.7673	2048	3	nr-er_SVM	0.7978	UNHASHED	2	0.054210425	0.0305
nr-er_RFC	0.7664	1024	1	nr-er_RFC	0.7969	UNHASHED	1	0.054515051	0.0305
nr-er_RFC	0.7664	1024	1	nr-er_RFC	0.7971	UNHASHED	3	0.052913776	0.0307
nr-er_FEST	0.7654	256	3	nr-er_FEST	0.7963	UNHASHED	2	0.051676897	0.0309
nr-er_SVM	0.7673	2048	3	nr-er_SVM	0.7982	UNHASHED	3	0.051057309	0.0309
nr-er_RFC	0.7664	1024	1	nr-er_RFC	0.7974	UNHASHED	2	0.05058474	0.031
nr-er_FEST	0.7654	256	3	nr-er_FEST	0.7965	UNHASHED	1	0.050147189	0.0311
nr-er_RFC	0.7658	256	3	nr-er_RFC	0.7969	UNHASHED	1	0.050019485	0.0311
nr-er_RFC	0.777	1024	2	nr-er_RFC	0.7645	4096	1	0.437727751	-0.0125
cas-N6512_SVM	0.7725	2048	2	cas-N6512_SVM	0.7607	4096	1	0.148999758	-0.0118
nr-er_SVM	0.7725	2048	2	nr-er_SVM	0.7607	4096	1	0.466134418	-0.0118
nr-er_FEST	0.779	1024	2	nr-er_FEST	0.7697	4096	1	0.561901596	-0.0093

nr-er_FEST	0.779	2048	2	nr-er_FEST	0.7697	4096	1	0.561901596	-0.0093
cas-N6512_SVM	0.7694	1024	2	cas-N6512_SVM	0.7607	4096	1	0.288725856	-0.0087
nr-er_SVM	0.7694	1024	2	nr-er_SVM	0.7607	4096	1	0.591766225	-0.0087
cas-N6512_SVM	0.773	4096	2	cas-N6512_SVM	0.7649	4096	3	0.319813832	-0.0081
nr-er_SVM	0.773	4096	2	nr-er_SVM	0.7649	4096	3	0.6158869	-0.0081
nr-er_RFC	0.7725	2048	2	nr-er_RFC	0.7645	4096	1	0.620447963	-0.008
nr-er_RFC	0.7722	1024	3	nr-er_RFC	0.7645	4096	1	0.633679831	-0.0077
cas-N6512_SVM	0.7725	2048	2	cas-N6512_SVM	0.7649	4096	3	0.350823504	-0.0076
nr-er_SVM	0.7725	2048	2	nr-er_SVM	0.7649	4096	3	0.637948341	-0.0076
cas-N6512_FEST	0.888	4096	1	cas-N6512_FEST	0.8811	4096	3	0.239497057	-0.0069
cas-N6512_FEST	0.8878	1024	1	cas-N6512_FEST	0.8811	4096	3	0.253596873	-0.0067
cas-N6512_SVM	0.7673	2048	3	cas-N6512_SVM	0.7607	4096	1	0.42180387	-0.0066
nr-er_SVM	0.7673	2048	3	nr-er_SVM	0.7607	4096	1	0.684517732	-0.0066
cas-N6512_FEST	0.8876	2048	1	cas-N6512_FEST	0.8811	4096	3	0.268251491	-0.0065
nr-er_RFC	0.7699	2048	3	nr-er_RFC	0.7645	4096	1	0.738589541	-0.0054
cas-N6512_FEST	0.8864	4096	2	cas-N6512_FEST	0.8811	4096	3	0.367918344	-0.0053
nr-er_FEST	0.775	2048	3	nr-er_FEST	0.7697	4096	1	0.741647514	-0.0053
cas-N6512_RFC	0.8856	4096	2	cas-N6512_RFC	0.8808	4096	3	0.415919714	-0.0048
cas-N6512_SVM	0.7694	1024	2	cas-N6512_SVM	0.7649	4096	3	0.581743115	-0.0045
nr-er_SVM	0.7694	1024	2	nr-er_SVM	0.7649	4096	3	0.780949506	-0.0045
cas-N6512_RFC	0.8852	2048	2	cas-N6512_RFC	0.8808	4096	3	0.456216906	-0.0044
cas-N6512_FEST	0.8852	1024	2	cas-N6512_FEST	0.8811	4096	3	0.487223074	-0.0041
cas-N6512_FEST	0.8852	2048	2	cas-N6512_FEST	0.8811	4096	3	0.487223074	-0.0041
cas-N6512_SVM	0.7648	256	2	cas-N6512_SVM	0.7607	4096	1	0.618560078	-0.0041
nr-er_SVM	0.7648	256	2	nr-er_SVM	0.7607	4096	1	0.801025341	-0.0041
nr-er_RFC	0.777	1024	2	nr-er_RFC	0.7735	4096	2	0.827016696	-0.0035
nr-ahr_RFC	0.91	4096	2	nr-ahr_RFC	0.9067	4096	3	0.759027634	-0.0033
nr-ahr_SVM	0.908	2048	2	nr-ahr_SVM	0.9047	4096	3	0.761199852	-0.0033
sr-mmp_FEST	0.8832	2048	2	sr-mmp_FEST	0.8801	4096	1	0.772777791	-0.0031
nr-ahr_SVM	0.9077	2048	1	nr-ahr_SVM	0.9047	4096	3	0.782482149	-0.003
cas-N6512_RFC	0.8852	2048	2	cas-N6512_RFC	0.8823	4096	1	0.622254862	-0.0029
sr-mmp_RFC	0.8859	2048	2	sr-mmp_RFC	0.883	4096	1	0.784983874	-0.0029
nr-ahr_RFC	0.9096	2048	2	nr-ahr_RFC	0.9067	4096	3	0.787684101	-0.0029

cas-N6512_RFC	0.8836	2048	1	cas-N6512_RFC	0.8808	4096	3	0.636541055	-0.0028	
cas-N6512_FEST	0.8838	256	1	cas-N6512_FEST	0.8811	4096	3	0.648284798	-0.0027	
nr-ahr_FEST	0.9104	4096	2	nr-ahr_FEST	0.9077	4096	3	0.801181639	-0.0027	
sr-mmp_FEST	0.8828	1024	2	sr-mmp_FEST	0.8801	4096	1	0.801575714	-0.0027	
cas-N6512_RFC	0.8834	1024	2	cas-N6512_RFC	0.8808	4096	3	0.660949859	-0.0026	
nr-er_FEST	0.7723	1024	3	nr-er_FEST	0.7697	4096	1	0.87174439	-0.0026	
sr-mmp_SVM	0.892	4096	2	sr-mmp_SVM	0.8896	4096	3	0.8171316	-0.0024	
cas-N6512_SVM	0.7673	2048	3	cas-N6512_SVM	0.7649	4096	3	0.769357749	-0.0024	
sr-mmp_FEST	0.8825	2048	3	sr-mmp_FEST	0.8801	4096	1	0.823327693	-0.0024	
nr-er_SVM	0.7673	2048	3	nr-er_SVM	0.7649	4096	3	0.882250197	-0.0024	
nr-ahr_SVM	0.907	4096	2	nr-ahr_SVM	0.9047	4096	3	0.832634369	-0.0023	
nr-er_FEST	0.7816	4096	2	nr-er_FEST	0.7795	4096	3	0.89493809	-0.0021	
nr-ahr_SVM	0.9067	4096	1	nr-ahr_SVM	0.9047	4096	3	0.854301076	-0.002	
nr-er_RFC	0.7664	1024	1	nr-er_RFC	0.7645	4096	1	0.906728088	-0.0019	
cas-N6512_SVM	0.7626	256	1	cas-N6512_SVM	0.7607	4096	1	0.817876367	-0.0019	
nr-er_SVM	0.7626	256	1	nr-er_SVM	0.7607	4096	1	0.907147859	-0.0019	
nr-ahr_FEST	0.9095	2048	2	nr-ahr_FEST	0.9077	4096	3	0.866962511	-0.0018	
nr-ahr_RFC	0.9096	2048	2	nr-ahr_RFC	0.908	4096	1	0.881517201	-0.0016	
cas-N6512_FEST	0.888	4096	1	cas-N6512_FEST	0.8864	4096	2	0.782603464	-0.0016	
nr-ahr_RFC	0.9083	2048	1	nr-ahr_RFC	0.9067	4096	3	0.882236481	-0.0016	
sr-mmp_RFC	0.8885	4096	2	sr-mmp_RFC	0.8869	4096	3	0.878917671	-0.0016	
cas-N6512_RFC	0.8823	4096	1	cas-N6512_RFC	0.8808	4096	3	0.80069047	-0.0015	
cas-N6512_FEST	0.8878	1024	1	cas-N6512_FEST	0.8864	4096	2	0.809299452	-0.0014	
cas-N6512_RFC	0.8822	1024	1	cas-N6512_RFC	0.8808	4096	3	0.813763399	-0.0014	
cas-N6512_RFC	0.8836	2048	1	cas-N6512_RFC	0.8823	4096	1	0.825799393	-0.0013	
nr-er_RFC	0.7658	256	3	nr-er_RFC	0.7645	4096	1	0.936128146	-0.0013	
nr-ahr_RFC	0.908	4096	1	nr-ahr_RFC	0.9067	4096	3	0.904265514	-0.0013	
nr-ahr_SVM	0.908	2048	2	nr-ahr_SVM	0.9067	4096	1	0.904265514	-0.0013	
cas-N6512_FEST	0.8876	2048	1	cas-N6512_FEST	0.8864	4096	2	0.836195294	-0.0012	
nr-ahr_FEST	0.9095	2048	2	nr-ahr_FEST	0.9083	4096	1	0.910952273	-0.0012	
nr-ahr_FEST	0.9088	2048	1	nr-ahr_FEST	0.9077	4096	3	0.918596388	-0.0011	
cas-N6512_RFC	0.8834	1024	2	cas-N6512_RFC	0.8823	4096	1	0.852325208	-0.0011	
sr-mmp_RFC	0.8841	1024	2	sr-mmp_RFC	0.883	4096	1	0.91784381	-0.0011	

sr-mmp_RFC	0.8841	1024	3	sr-mmp_RFC	0.883	4096	1	0.91784381	-0.0011	
sr-mmp_RFC	0.8841	2048	3	sr-mmp_RFC	0.883	4096	1	0.91784381	-0.0011	
nr-ahr_SVM	0.9077	2048	1	nr-ahr_SVM	0.9067	4096	1	0.926337282	-0.001	
nr-ahr_SVM	0.908	2048	2	nr-ahr_SVM	0.907	4096	2	0.926234289	-0.001	
cas-N6512_SVM	0.7616	1024	1	cas-N6512_SVM	0.7607	4096	1	0.913217084	-0.0009	
cas-N6512_SVM	0.7616	2048	1	cas-N6512_SVM	0.7607	4096	1	0.913217084	-0.0009	
nr-ahr_FEST	0.9086	1024	2	nr-ahr_FEST	0.9077	4096	3	0.933390239	-0.0009	
nr-ahr_RFC	0.9076	1024	2	nr-ahr_RFC	0.9067	4096	3	0.933701091	-0.0009	
nr-er_SVM	0.7616	1024	1	nr-er_SVM	0.7607	4096	1	0.955966056	-0.0009	
nr-er_SVM	0.7616	2048	1	nr-er_SVM	0.7607	4096	1	0.955966056	-0.0009	
nr-ahr_RFC	0.9074	1024	1	nr-ahr_RFC	0.9067	4096	3	0.948434755	-0.0007	
nr-ahr_SVM	0.9077	2048	1	nr-ahr_SVM	0.907	4096	2	0.948362691	-0.0007	
nr-ahr_FEST	0.9083	4096	1	nr-ahr_FEST	0.9077	4096	3	0.955596257	-0.0006	
sr-mmp_FEST	0.8807	2048	1	sr-mmp_FEST	0.8801	4096	1	0.955627874	-0.0006	
nr-ahr_FEST	0.9088	2048	1	nr-ahr_FEST	0.9083	4096	1	0.962894812	-0.0005	
sr-mmp_FEST	0.8806	1024	3	sr-mmp_FEST	0.8801	4096	1	0.963023843	-0.0005	
nr-ahr_RFC	0.9083	2048	1	nr-ahr_RFC	0.908	4096	1	0.977773866	-0.0003	
nr-ahr_SVM	0.905	1024	1	nr-ahr_SVM	0.9047	4096	3	0.97811015	-0.0003	
cas-N6512_RFC	0.8811	2048	3	cas-N6512_RFC	0.8808	4096	3	0.959832645	-0.0003	
sr-mmp_SVM	0.888	2048	2	sr-mmp_SVM	0.8877	4096	1	0.97719968	-0.0003	
nr-ahr_FEST	0.9086	1024	2	nr-ahr_FEST	0.9083	4096	1	0.977742329	-0.0003	
cas-N6512_SVM	0.7609	1024	3	cas-N6512_SVM	0.7607	4096	1	0.980690647	-0.0002	
nr-er_SVM	0.7609	1024	3	nr-er_SVM	0.7607	4096	1	0.990213978	-0.0002	
nr-er_RFC	0.7646	256	2	nr-er_RFC	0.7645	4096	1	0.995085139	-1E-04	
cas-N6512_RFC	0.8822	1024	1	cas-N6512_RFC	0.8823	4096	1	0.98653249	1E-04	
cas-N6512_SVM	0.7648	256	2	cas-N6512_SVM	0.7649	4096	3	0.990274484	1E-04	
nr-er_RFC	0.777	1024	2	nr-er_RFC	0.7771	4096	3	0.995006441	1E-04	
nr-er_SVM	0.7648	256	2	nr-er_SVM	0.7649	4096	3	0.995083363	1E-04	
cas-N6512_FEST	0.8878	1024	1	cas-N6512_FEST	0.888	4096	1	0.972400156	0.0002	
nr-ahr_SVM	0.9067	4096	1	nr-ahr_SVM	0.907	4096	2	0.977908634	0.0003	
nr-ahr_RFC	0.9076	1024	2	nr-ahr_RFC	0.908	4096	1	0.97041688	0.0004	
sr-mmp_RFC	0.8826	1024	1	sr-mmp_RFC	0.883	4096	1	0.970159329	0.0004	
cas-N6512_FEST	0.8876	2048	1	cas-N6512_FEST	0.888	4096	1	0.944857764	0.0004	

cas-N6512_RFC	0.8852	2048	2	cas-N6512_RFC	0.8856	4096	2	0.945436687	0.0004
nr-ahr_RFC	0.9096	2048	2	nr-ahr_RFC	0.91	4096	2	0.970134137	0.0004
nr-er_FEST	0.779	1024	2	nr-er_FEST	0.7795	4096	3	0.97496164	0.0005
nr-er_FEST	0.779	2048	2	nr-er_FEST	0.7795	4096	3	0.97496164	0.0005
cas-N6512_SVM	0.7725	2048	2	cas-N6512_SVM	0.773	4096	2	0.950684358	0.0005
nr-er_SVM	0.7725	2048	2	nr-er_SVM	0.773	4096	2	0.975176924	0.0005
nr-ahr_RFC	0.9074	1024	1	nr-ahr_RFC	0.908	4096	1	0.955658764	0.0006
nr-ahr_RFC	0.9061	2048	3	nr-ahr_RFC	0.9067	4096	3	0.955925892	0.0006
nr-er_RFC	0.7639	2048	1	nr-er_RFC	0.7645	4096	1	0.970529677	0.0006
nr-ahr_FEST	0.9068	2048	3	nr-ahr_FEST	0.9077	4096	3	0.933670248	0.0009
nr-ahr_FEST	0.9095	2048	2	nr-ahr_FEST	0.9104	4096	2	0.93281678	0.0009
sr-mmp_RFC	0.8859	2048	2	sr-mmp_RFC	0.8869	4096	3	0.924511423	0.001
sr-mmp_RFC	0.882	2048	1	sr-mmp_RFC	0.883	4096	1	0.925568495	0.001
nr-er_RFC	0.7725	2048	2	nr-er_RFC	0.7735	4096	2	0.950361717	0.001
cas-N6512_FEST	0.8852	1024	2	cas-N6512_FEST	0.8864	4096	2	0.837045016	0.0012
cas-N6512_FEST	0.8852	2048	2	cas-N6512_FEST	0.8864	4096	2	0.837045016	0.0012
cas-N6512_RFC	0.8811	2048	3	cas-N6512_RFC	0.8823	4096	1	0.839846298	0.0012
cas-N6512_FEST	0.8799	2048	3	cas-N6512_FEST	0.8811	4096	3	0.840637673	0.0012
nr-er_RFC	0.7722	1024	3	nr-er_RFC	0.7735	4096	2	0.935511353	0.0013
sr-mmp_FEST	0.8832	2048	2	sr-mmp_FEST	0.8846	4096	2	0.895421136	0.0014
nr-ahr_FEST	0.9068	2048	3	nr-ahr_FEST	0.9083	4096	1	0.889522338	0.0015
sr-mmp_SVM	0.888	2048	2	sr-mmp_SVM	0.8896	4096	3	0.878417324	0.0016
nr-ahr_FEST	0.9088	2048	1	nr-ahr_FEST	0.9104	4096	2	0.881066363	0.0016
nr-ahr_SVM	0.9031	1024	2	nr-ahr_SVM	0.9047	4096	3	0.884146446	0.0016
nr-ahr_SVM	0.905	1024	1	nr-ahr_SVM	0.9067	4096	1	0.875879187	0.0017
nr-ahr_RFC	0.9083	2048	1	nr-ahr_RFC	0.91	4096	2	0.87396335	0.0017
sr-mmp_FEST	0.8828	1024	2	sr-mmp_FEST	0.8846	4096	2	0.865888628	0.0018
nr-ahr_FEST	0.9086	1024	2	nr-ahr_FEST	0.9104	4096	2	0.866395099	0.0018
nr-ahr_RFC	0.9061	2048	3	nr-ahr_RFC	0.908	4096	1	0.860654215	0.0019
sr-mmp_SVM	0.8877	4096	1	sr-mmp_SVM	0.8896	4096	3	0.855931344	0.0019
cas-N6512_RFC	0.8836	2048	1	cas-N6512_RFC	0.8856	4096	2	0.733097085	0.002
sr-mmp_SVM	0.8857	1024	1	sr-mmp_SVM	0.8877	4096	1	0.849530579	0.002
sr-mmp_SVM	0.8857	2048	3	sr-mmp_SVM	0.8877	4096	1	0.849530579	0.002

nr-ahr_SVM	0.905	1024	1	nr-ahr_SVM	0.907	4096	2	0.854102867	0.002
nr-ahr_RFC	0.908	4096	1	nr-ahr_RFC	0.91	4096	2	0.852063631	0.002
nr-ahr_FEST	0.9083	4096	1	nr-ahr_FEST	0.9104	4096	2	0.844501777	0.0021
sr-mmp_FEST	0.8825	2048	3	sr-mmp_FEST	0.8846	4096	2	0.843886619	0.0021
cas-N6512_RFC	0.8834	1024	2	cas-N6512_RFC	0.8856	4096	2	0.707699435	0.0022
cas-N6512_SVM	0.7626	256	1	cas-N6512_SVM	0.7649	4096	3	0.779627562	0.0023
nr-er_SVM	0.7626	256	1	nr-er_SVM	0.7649	4096	3	0.887440489	0.0023
nr-ahr_SVM	0.9023	2048	3	nr-ahr_SVM	0.9047	4096	3	0.827285509	0.0024
nr-ahr_RFC	0.9076	1024	2	nr-ahr_RFC	0.91	4096	2	0.823091098	0.0024
sr-mmp_FEST	0.8776	1024	1	sr-mmp_FEST	0.8801	4096	1	0.817621566	0.0025
nr-ahr_FEST	0.9052	1024	1	nr-ahr_FEST	0.9077	4096	3	0.81783107	0.0025
cas-N6512_FEST	0.8838	256	1	cas-N6512_FEST	0.8864	4096	2	0.656845288	0.0026
sr-mmp_RFC	0.8859	2048	2	sr-mmp_RFC	0.8885	4096	2	0.804835697	0.0026
sr-mmp_FEST	0.8846	4096	2	sr-mmp_FEST	0.8872	4096	3	0.805755646	0.0026
nr-er_FEST	0.779	1024	2	nr-er_FEST	0.7816	4096	2	0.870168064	0.0026
nr-er_FEST	0.779	2048	2	nr-er_FEST	0.7816	4096	2	0.870168064	0.0026
nr-ahr_RFC	0.9074	1024	1	nr-ahr_RFC	0.91	4096	2	0.80871247	0.0026
nr-er_FEST	0.767	1024	1	nr-er_FEST	0.7697	4096	1	0.867295595	0.0027
cas-N6512_FEST	0.8852	1024	2	cas-N6512_FEST	0.888	4096	1	0.630092468	0.0028
cas-N6512_FEST	0.8852	2048	2	cas-N6512_FEST	0.888	4096	1	0.630092468	0.0028
sr-mmp_RFC	0.8841	1024	2	sr-mmp_RFC	0.8869	4096	3	0.791446727	0.0028
sr-mmp_RFC	0.8841	1024	3	sr-mmp_RFC	0.8869	4096	3	0.791446727	0.0028
sr-mmp_RFC	0.8841	2048	3	sr-mmp_RFC	0.8869	4096	3	0.791446727	0.0028
nr-er_FEST	0.7668	2048	1	nr-er_FEST	0.7697	4096	1	0.857584418	0.0029
nr-ahr_FEST	0.9052	1024	1	nr-ahr_FEST	0.9083	4096	1	0.774866811	0.0031
cas-N6512_RFC	0.8776	256	1	cas-N6512_RFC	0.8808	4096	3	0.593802444	0.0032
nr-er_RFC	0.7613	256	1	nr-er_RFC	0.7645	4096	1	0.844036975	0.0032
cas-N6512_SVM	0.7616	1024	1	cas-N6512_SVM	0.7649	4096	3	0.688346625	0.0033
cas-N6512_SVM	0.7616	2048	1	cas-N6512_SVM	0.7649	4096	3	0.688346625	0.0033
nr-er_SVM	0.7616	1024	1	nr-er_SVM	0.7649	4096	3	0.839162588	0.0033
nr-er_SVM	0.7616	2048	1	nr-er_SVM	0.7649	4096	3	0.839162588	0.0033
cas-N6512_RFC	0.8823	4096	1	cas-N6512_RFC	0.8856	4096	2	0.574735077	0.0033
cas-N6512_RFC	0.8822	1024	1	cas-N6512_RFC	0.8856	4096	2	0.563284118	0.0034

nr-ahr_FEST	0.9068	2048	3	nr-ahr_FEST	0.9104	4096	2	0.737592588	0.0036	
nr-ahr_SVM	0.9031	1024	2	nr-ahr_SVM	0.9067	4096	1	0.741894998	0.0036	
nr-er_RFC	0.7699	2048	3	nr-er_RFC	0.7735	4096	2	0.822961087	0.0036	
cas-N6512_FEST	0.8775	1024	3	cas-N6512_FEST	0.8811	4096	3	0.54834092	0.0036	
cas-N6512_SVM	0.7694	1024	2	cas-N6512_SVM	0.773	4096	2	0.657040755	0.0036	
nr-er_RFC	0.7735	4096	2	nr-er_RFC	0.7771	4096	3	0.822144153	0.0036	
nr-er_SVM	0.7694	1024	2	nr-er_SVM	0.773	4096	2	0.823072267	0.0036	
sr-mmp_SVM	0.8857	1024	1	sr-mmp_SVM	0.8896	4096	3	0.710435856	0.0039	
sr-mmp_SVM	0.8857	2048	3	sr-mmp_SVM	0.8896	4096	3	0.710435856	0.0039	
sr-mmp_SVM	0.8838	2048	1	sr-mmp_SVM	0.8877	4096	1	0.712381712	0.0039	
sr-mmp_RFC	0.883	4096	1	sr-mmp_RFC	0.8869	4096	3	0.713185692	0.0039	
sr-mmp_FEST	0.8807	2048	1	sr-mmp_FEST	0.8846	4096	2	0.715448257	0.0039	
nr-ahr_RFC	0.9061	2048	3	nr-ahr_RFC	0.91	4096	2	0.71733554	0.0039	
nr-ahr_SVM	0.9031	1024	2	nr-ahr_SVM	0.907	4096	2	0.72106592	0.0039	
cas-N6512_SVM	0.7609	1024	3	cas-N6512_SVM	0.7649	4096	3	0.627053352	0.004	
sr-mmp_SVM	0.888	2048	2	sr-mmp_SVM	0.892	4096	2	0.700823725	0.004	
sr-mmp_FEST	0.8832	2048	2	sr-mmp_FEST	0.8872	4096	3	0.705912895	0.004	
sr-mmp_FEST	0.8806	1024	3	sr-mmp_FEST	0.8846	4096	2	0.708530691	0.004	
nr-er_SVM	0.7609	1024	3	nr-er_SVM	0.7649	4096	3	0.805746484	0.004	
cas-N6512_FEST	0.8838	256	1	cas-N6512_FEST	0.888	4096	1	0.471413776	0.0042	
cas-N6512_RFC	0.8766	1024	3	cas-N6512_RFC	0.8808	4096	3	0.484822212	0.0042	
cas-N6512_SVM	0.7607	4096	1	cas-N6512_SVM	0.7649	4096	3	0.609994279	0.0042	
nr-er_SVM	0.7607	4096	1	nr-er_SVM	0.7649	4096	3	0.796265919	0.0042	
cas-N6512_SVM	0.7565	128	1	cas-N6512_SVM	0.7607	4096	1	0.612633983	0.0042	
nr-er_SVM	0.7565	128	1	nr-er_SVM	0.7607	4096	1	0.797236862	0.0042	
sr-mmp_SVM	0.8877	4096	1	sr-mmp_SVM	0.892	4096	2	0.67976217	0.0043	
sr-mmp_RFC	0.8826	1024	1	sr-mmp_RFC	0.8869	4096	3	0.685485924	0.0043	
nr-er_FEST	0.7654	256	3	nr-er_FEST	0.7697	4096	1	0.790352214	0.0043	
sr-mmp_RFC	0.8841	1024	2	sr-mmp_RFC	0.8885	4096	2	0.676846287	0.0044	
sr-mmp_RFC	0.8841	1024	3	sr-mmp_RFC	0.8885	4096	2	0.676846287	0.0044	
sr-mmp_RFC	0.8841	2048	3	sr-mmp_RFC	0.8885	4096	2	0.676846287	0.0044	
sr-mmp_FEST	0.8828	1024	2	sr-mmp_FEST	0.8872	4096	3	0.678299285	0.0044	
nr-ahr_SVM	0.9023	2048	3	nr-ahr_SVM	0.9067	4096	1	0.687823228	0.0044	

sr-mmp_SVM	0.8833	1024	2	sr-mmp_SVM	0.8877	4096	1	0.677743519	0.0044	
nr-er_FEST	0.775	2048	3	nr-er_FEST	0.7795	4096	3	0.778159699	0.0045	
cas-N6512_RFC	0.8811	2048	3	cas-N6512_RFC	0.8856	4096	2	0.445366101	0.0045	
sr-mmp_FEST	0.8801	4096	1	sr-mmp_FEST	0.8846	4096	2	0.674347828	0.0045	
sr-mmp_RFC	0.8784	256	1	sr-mmp_RFC	0.883	4096	1	0.669348799	0.0046	
nr-er_RFC	0.7725	2048	2	nr-er_RFC	0.7771	4096	3	0.774079796	0.0046	
cas-N6512_RFC	0.8776	256	1	cas-N6512_RFC	0.8823	4096	1	0.432004868	0.0047	
sr-mmp_FEST	0.8825	2048	3	sr-mmp_FEST	0.8872	4096	3	0.657891967	0.0047	
nr-ahr_SVM	0.9023	2048	3	nr-ahr_SVM	0.907	4096	2	0.667559553	0.0047	
nr-ahr_FEST	0.9029	1024	3	nr-ahr_FEST	0.9077	4096	3	0.659985421	0.0048	
sr-mmp_FEST	0.8753	256	1	sr-mmp_FEST	0.8801	4096	1	0.65917982	0.0048	
nr-er_RFC	0.7596	128	1	nr-er_RFC	0.7645	4096	1	0.763453812	0.0049	
sr-mmp_RFC	0.882	2048	1	sr-mmp_RFC	0.8869	4096	3	0.644798721	0.0049	
nr-er_RFC	0.7722	1024	3	nr-er_RFC	0.7771	4096	3	0.759830596	0.0049	
nr-er_FEST	0.7646	256	2	nr-er_FEST	0.7697	4096	1	0.752639206	0.0051	
nr-ahr_FEST	0.9052	1024	1	nr-ahr_FEST	0.9104	4096	2	0.629706676	0.0052	
nr-ahr_RFC	0.9014	256	1	nr-ahr_RFC	0.9067	4096	3	0.629074739	0.0053	
nr-ahr_FEST	0.9029	1024	3	nr-ahr_FEST	0.9083	4096	1	0.620170803	0.0054	
sr-mmp_RFC	0.883	4096	1	sr-mmp_RFC	0.8885	4096	2	0.603122091	0.0055	
nr-ahr_FEST	0.9022	256	1	nr-ahr_FEST	0.9077	4096	3	0.614755893	0.0055	
nr-ahr_RFC	0.9012	1024	3	nr-ahr_RFC	0.9067	4096	3	0.61634672	0.0055	
cas-N6512_RFC	0.8766	1024	3	cas-N6512_RFC	0.8823	4096	1	0.341610719	0.0057	
cas-N6512_SVM	0.7673	2048	3	cas-N6512_SVM	0.773	4096	2	0.482911885	0.0057	
nr-er_SVM	0.7673	2048	3	nr-er_SVM	0.773	4096	2	0.723667595	0.0057	
sr-mmp_SVM	0.8838	2048	1	sr-mmp_SVM	0.8896	4096	3	0.582177694	0.0058	
nr-ahr_SVM	0.8989	256	1	nr-ahr_SVM	0.9047	4096	3	0.600714882	0.0058	
sr-mmp_RFC	0.8826	1024	1	sr-mmp_RFC	0.8885	4096	2	0.577307183	0.0059	
nr-er_FEST	0.7638	256	1	nr-er_FEST	0.7697	4096	1	0.715542662	0.0059	
cas-N6512_FEST	0.875	256	2	cas-N6512_FEST	0.8811	4096	3	0.311582814	0.0061	
cas-N6512_SVM	0.7546	128	2	cas-N6512_SVM	0.7607	4096	1	0.462849986	0.0061	
nr-ahr_FEST	0.9022	256	1	nr-ahr_FEST	0.9083	4096	1	0.576182386	0.0061	
nr-er_SVM	0.7546	128	2	nr-er_SVM	0.7607	4096	1	0.709329364	0.0061	
nr-ahr_RFC	0.9005	256	2	nr-ahr_RFC	0.9067	4096	3	0.572802374	0.0062	

sr-mmp_SVM	0.8857	1024	1	sr-mmp_SVM	0.892	4096	2	0.546845685	0.0063	
sr-mmp_SVM	0.8857	2048	3	sr-mmp_SVM	0.892	4096	2	0.546845685	0.0063	
sr-mmp_SVM	0.8833	1024	2	sr-mmp_SVM	0.8896	4096	3	0.550447542	0.0063	
cas-N6512_FEST	0.8799	2048	3	cas-N6512_FEST	0.8864	4096	2	0.27071545	0.0065	
sr-mmp_RFC	0.882	2048	1	sr-mmp_RFC	0.8885	4096	2	0.539657019	0.0065	
sr-mmp_FEST	0.8807	2048	1	sr-mmp_FEST	0.8872	4096	3	0.541568868	0.0065	
nr-ahr_RFC	0.9014	256	1	nr-ahr_RFC	0.908	4096	1	0.546317104	0.0066	
sr-mmp_FEST	0.8806	1024	3	sr-mmp_FEST	0.8872	4096	3	0.535440058	0.0066	
sr-mmp_FEST	0.8735	256	2	sr-mmp_FEST	0.8801	4096	1	0.545429636	0.0066	
nr-er_FEST	0.775	2048	3	nr-er_FEST	0.7816	4096	2	0.679033762	0.0066	
cas-N6512_RFC	0.8742	256	2	cas-N6512_RFC	0.8808	4096	3	0.274652688	0.0066	
nr-ahr_RFC	0.9012	1024	3	nr-ahr_RFC	0.908	4096	1	0.534407498	0.0068	
cas-N6512_FEST	0.8741	128	1	cas-N6512_FEST	0.8811	4096	3	0.246419619	0.007	
sr-mmp_FEST	0.8776	1024	1	sr-mmp_FEST	0.8846	4096	2	0.515187368	0.007	
sr-mmp_SVM	0.8806	1024	3	sr-mmp_SVM	0.8877	4096	1	0.50459277	0.0071	
sr-mmp_FEST	0.8801	4096	1	sr-mmp_FEST	0.8872	4096	3	0.505360625	0.0071	
nr-er_RFC	0.7664	1024	1	nr-er_RFC	0.7735	4096	2	0.659721652	0.0071	
nr-er_FEST	0.7723	1024	3	nr-er_FEST	0.7795	4096	3	0.65274571	0.0072	
nr-er_RFC	0.7699	2048	3	nr-er_RFC	0.7771	4096	3	0.653767707	0.0072	
nr-ahr_RFC	0.9005	256	2	nr-ahr_RFC	0.908	4096	1	0.493859964	0.0075	
nr-ahr_FEST	0.9029	1024	3	nr-ahr_FEST	0.9104	4096	2	0.489117445	0.0075	
nr-er_RFC	0.7569	128	2	nr-er_RFC	0.7645	4096	1	0.64117584	0.0076	
sr-mmp_RFC	0.8754	256	2	sr-mmp_RFC	0.883	4096	1	0.482720107	0.0076	
nr-er_RFC	0.7658	256	3	nr-er_RFC	0.7735	4096	2	0.633127584	0.0077	
nr-ahr_SVM	0.8989	256	1	nr-ahr_SVM	0.9067	4096	1	0.479581346	0.0078	
nr-er_RFC	0.7566	128	3	nr-er_RFC	0.7645	4096	1	0.628123321	0.0079	
cas-N6512_RFC	0.8776	256	1	cas-N6512_RFC	0.8856	4096	2	0.178065288	0.008	
cas-N6512_SVM	0.7527	256	3	cas-N6512_SVM	0.7607	4096	1	0.336414261	0.008	
nr-er_SVM	0.7527	256	3	nr-er_SVM	0.7607	4096	1	0.625301634	0.008	
cas-N6512_FEST	0.8799	2048	3	cas-N6512_FEST	0.888	4096	1	0.168433398	0.0081	
cas-N6512_RFC	0.8742	256	2	cas-N6512_RFC	0.8823	4096	1	0.178692635	0.0081	
nr-ahr_SVM	0.8989	256	1	nr-ahr_SVM	0.907	4096	2	0.462546145	0.0081	
cas-N6512_SVM	0.7648	256	2	cas-N6512_SVM	0.773	4096	2	0.313920347	0.0082	

sr-mmp_SVM	0.8838	2048	1	sr-mmp_SVM	0.892	4096	2	0.434556723	0.0082
nr-ahr_FEST	0.9022	256	1	nr-ahr_FEST	0.9104	4096	2	0.450208602	0.0082
nr-er_SVM	0.7648	256	2	nr-er_SVM	0.773	4096	2	0.611557925	0.0082
nr-ahr_FEST	0.8993	256	2	nr-ahr_FEST	0.9077	4096	3	0.445022802	0.0084
nr-ahr_RFC	0.8983	128	1	nr-ahr_RFC	0.9067	4096	3	0.447035163	0.0084
cas-N6512_SVM	0.7565	128	1	cas-N6512_SVM	0.7649	4096	3	0.309439526	0.0084
sr-mmp_SVM	0.8793	256	1	sr-mmp_SVM	0.8877	4096	1	0.43090922	0.0084
nr-er_SVM	0.7565	128	1	nr-er_SVM	0.7649	4096	3	0.606469171	0.0084
sr-mmp_RFC	0.8784	256	1	sr-mmp_RFC	0.8869	4096	3	0.42684759	0.0085
sr-mmp_FEST	0.8715	128	1	sr-mmp_FEST	0.8801	4096	1	0.432303285	0.0086
nr-ahr_RFC	0.9014	256	1	nr-ahr_RFC	0.91	4096	2	0.429684905	0.0086
sr-mmp_SVM	0.8833	1024	2	sr-mmp_SVM	0.892	4096	2	0.407501951	0.0087
nr-ahr_RFC	0.9012	1024	3	nr-ahr_RFC	0.91	4096	2	0.419244407	0.0088
cas-N6512_FEST	0.8775	1024	3	cas-N6512_FEST	0.8864	4096	2	0.133494743	0.0089
nr-er_RFC	0.7646	256	2	nr-er_RFC	0.7735	4096	2	0.581418656	0.0089
sr-mmp_SVM	0.8806	1024	3	sr-mmp_SVM	0.8896	4096	3	0.395984832	0.009
cas-N6512_RFC	0.8766	1024	3	cas-N6512_RFC	0.8856	4096	2	0.130555554	0.009
nr-ahr_FEST	0.8993	256	2	nr-ahr_FEST	0.9083	4096	1	0.412556096	0.009
nr-er_RFC	0.7645	4096	1	nr-er_RFC	0.7735	4096	2	0.577203412	0.009
nr-er_FEST	0.7723	1024	3	nr-er_FEST	0.7816	4096	2	0.560552515	0.0093
sr-mmp_FEST	0.8753	256	1	sr-mmp_FEST	0.8846	4096	2	0.389124849	0.0093
nr-ahr_RFC	0.9005	256	2	nr-ahr_RFC	0.91	4096	2	0.383964201	0.0095
sr-mmp_FEST	0.8776	1024	1	sr-mmp_FEST	0.8872	4096	3	0.369901247	0.0096
nr-er_RFC	0.7639	2048	1	nr-er_RFC	0.7735	4096	2	0.55222938	0.0096
nr-ahr_RFC	0.8983	128	1	nr-ahr_RFC	0.908	4096	1	0.378531187	0.0097
nr-er_FEST	0.7697	4096	1	nr-er_FEST	0.7795	4096	3	0.540928664	0.0098
sr-mmp_FEST	0.8702	256	3	sr-mmp_FEST	0.8801	4096	1	0.367030017	0.0099
sr-mmp_RFC	0.8784	256	1	sr-mmp_RFC	0.8885	4096	2	0.343681858	0.0101
cas-N6512_SVM	0.7546	128	2	cas-N6512_SVM	0.7649	4096	3	0.213409132	0.0103
sr-mmp_SVM	0.8793	256	1	sr-mmp_SVM	0.8896	4096	3	0.332463939	0.0103
nr-er_SVM	0.7546	128	2	nr-er_SVM	0.7649	4096	3	0.528065691	0.0103
cas-N6512_SVM	0.7626	256	1	cas-N6512_SVM	0.773	4096	2	0.202435211	0.0104
nr-er_SVM	0.7626	256	1	nr-er_SVM	0.773	4096	2	0.5200632	0.0104

cas-N6512_FEST	0.8775	1024	3	cas-N6512_FEST	0.888	4096	1	0.075694661	0.0105
nr-er_RFC	0.7664	1024	1	nr-er_RFC	0.7771	4096	3	0.505981551	0.0107
nr-er_FEST	0.7589	128	2	nr-er_FEST	0.7697	4096	1	0.505954957	0.0108
sr-mmp_FEST	0.8735	256	2	sr-mmp_FEST	0.8846	4096	2	0.305473189	0.0111
nr-ahr_FEST	0.8993	256	2	nr-ahr_FEST	0.9104	4096	2	0.309884191	0.0111
nr-ahr_SVM	0.8935	1024	3	nr-ahr_SVM	0.9047	4096	3	0.317706744	0.0112
nr-er_RFC	0.7658	256	3	nr-er_RFC	0.7771	4096	3	0.48259001	0.0113
cas-N6512_SVM	0.7616	1024	1	cas-N6512_SVM	0.773	4096	2	0.162732862	0.0114
cas-N6512_SVM	0.7616	2048	1	cas-N6512_SVM	0.773	4096	2	0.162732862	0.0114
sr-mmp_SVM	0.8806	1024	3	sr-mmp_SVM	0.892	4096	2	0.280141663	0.0114
nr-er_SVM	0.7616	1024	1	nr-er_SVM	0.773	4096	2	0.481010578	0.0114
nr-er_SVM	0.7616	2048	1	nr-er_SVM	0.773	4096	2	0.481010578	0.0114
cas-N6512_FEST	0.875	256	2	cas-N6512_FEST	0.8864	4096	2	0.055885064	0.0114
cas-N6512_RFC	0.8742	256	2	cas-N6512_RFC	0.8856	4096	2	0.056703586	0.0114
sr-mmp_RFC	0.8754	256	2	sr-mmp_RFC	0.8869	4096	3	0.284860688	0.0115
nr-ahr_RFC	0.8983	128	1	nr-ahr_RFC	0.91	4096	2	0.285967015	0.0117
nr-er_FEST	0.7697	4096	1	nr-er_FEST	0.7816	4096	2	0.457186411	0.0119
sr-mmp_FEST	0.8753	256	1	sr-mmp_FEST	0.8872	4096	3	0.268265196	0.0119
cas-N6512_SVM	0.7609	1024	3	cas-N6512_SVM	0.773	4096	2	0.138681937	0.0121
sr-mmp_RFC	0.8709	128	1	sr-mmp_RFC	0.883	4096	1	0.267343502	0.0121
nr-er_SVM	0.7609	1024	3	nr-er_SVM	0.773	4096	2	0.454678836	0.0121
cas-N6512_SVM	0.7527	256	3	cas-N6512_SVM	0.7649	4096	3	0.141193903	0.0122
nr-er_RFC	0.7613	256	1	nr-er_RFC	0.7735	4096	2	0.450707599	0.0122
nr-er_SVM	0.7527	256	3	nr-er_SVM	0.7649	4096	3	0.455324789	0.0122
cas-N6512_SVM	0.7607	4096	1	cas-N6512_SVM	0.773	4096	2	0.132344567	0.0123
nr-er_SVM	0.7607	4096	1	nr-er_SVM	0.773	4096	2	0.447311154	0.0123
sr-mmp_RFC	0.8706	256	3	sr-mmp_RFC	0.883	4096	1	0.255896778	0.0124
nr-ahr_SVM	0.8923	256	2	nr-ahr_SVM	0.9047	4096	3	0.269807658	0.0124
nr-er_FEST	0.7573	128	3	nr-er_FEST	0.7697	4096	1	0.445471563	0.0124
nr-er_FEST	0.767	1024	1	nr-er_FEST	0.7795	4096	3	0.436255889	0.0125
nr-er_RFC	0.7646	256	2	nr-er_RFC	0.7771	4096	3	0.437669523	0.0125
nr-er_RFC	0.7645	4096	1	nr-er_RFC	0.7771	4096	3	0.43404072	0.0126
nr-ahr_FEST	0.895	128	1	nr-ahr_FEST	0.9077	4096	3	0.252607532	0.0127

nr-er_FEST	0.7668	2048	1	nr-er_FEST	0.7795	4096	3	0.429008745	0.0127
sr-mmp_SVM	0.8793	256	1	sr-mmp_SVM	0.892	4096	2	0.230008032	0.0127
sr-mmp_RFC	0.8754	256	2	sr-mmp_RFC	0.8885	4096	2	0.221796056	0.0131
sr-mmp_FEST	0.8715	128	1	sr-mmp_FEST	0.8846	4096	2	0.228056548	0.0131
nr-ahr_SVM	0.8935	1024	3	nr-ahr_SVM	0.9067	4096	1	0.236917681	0.0132
nr-er_RFC	0.7639	2048	1	nr-er_RFC	0.7771	4096	3	0.412644698	0.0132
nr-er_FEST	0.7565	128	1	nr-er_FEST	0.7697	4096	1	0.416883173	0.0132
nr-ahr_FEST	0.895	128	1	nr-ahr_FEST	0.9083	4096	1	0.230261219	0.0133
nr-ahr_SVM	0.8935	1024	3	nr-ahr_SVM	0.907	4096	2	0.226125184	0.0135
sr-mmp_FEST	0.8735	256	2	sr-mmp_FEST	0.8872	4096	3	0.203866277	0.0137
nr-er_RFC	0.7596	128	1	nr-er_RFC	0.7735	4096	2	0.390630974	0.0139
nr-er_FEST	0.7654	256	3	nr-er_FEST	0.7795	4096	3	0.380317442	0.0141
sr-mmp_FEST	0.8657	128	2	sr-mmp_FEST	0.8801	4096	1	0.19267613	0.0144
nr-ahr_SVM	0.8923	256	2	nr-ahr_SVM	0.9067	4096	1	0.198082584	0.0144
sr-mmp_FEST	0.8702	256	3	sr-mmp_FEST	0.8846	4096	2	0.186115067	0.0144
nr-ahr_RFC	0.8922	256	3	nr-ahr_RFC	0.9067	4096	3	0.195077692	0.0145
nr-er_FEST	0.767	1024	1	nr-er_FEST	0.7816	4096	2	0.362494815	0.0146
nr-ahr_SVM	0.8923	256	2	nr-ahr_SVM	0.907	4096	2	0.188608986	0.0147
nr-er_FEST	0.7668	2048	1	nr-er_FEST	0.7816	4096	2	0.356017466	0.0148
nr-er_FEST	0.7646	256	2	nr-er_FEST	0.7795	4096	3	0.354117596	0.0149
nr-ahr_RFC	0.8916	128	2	nr-ahr_RFC	0.9067	4096	3	0.177770872	0.0151
nr-ahr_FEST	0.895	128	1	nr-ahr_FEST	0.9104	4096	2	0.162812027	0.0154
sr-mmp_FEST	0.8715	128	1	sr-mmp_FEST	0.8872	4096	3	0.146721989	0.0157
nr-er_FEST	0.7638	256	1	nr-er_FEST	0.7795	4096	3	0.329110647	0.0157
nr-ahr_RFC	0.8922	256	3	nr-ahr_RFC	0.908	4096	1	0.156809022	0.0158
nr-er_RFC	0.7613	256	1	nr-er_RFC	0.7771	4096	3	0.327539468	0.0158
sr-mmp_RFC	0.8709	128	1	sr-mmp_RFC	0.8869	4096	3	0.139762212	0.016
nr-ahr_FEST	0.8915	256	3	nr-ahr_FEST	0.9077	4096	3	0.147428404	0.0162
nr-ahr_SVM	0.8885	128	1	nr-ahr_SVM	0.9047	4096	3	0.152531432	0.0162
nr-er_FEST	0.7654	256	3	nr-er_FEST	0.7816	4096	2	0.312778004	0.0162
sr-mmp_RFC	0.8706	256	3	sr-mmp_RFC	0.8869	4096	3	0.132680577	0.0163
nr-ahr_RFC	0.8916	128	2	nr-ahr_RFC	0.908	4096	1	0.142151575	0.0164
nr-er_SVM	0.7565	128	1	nr-er_SVM	0.773	4096	2	0.309199865	0.0165

nr-er_RFC	0.7569	128	2	nr-er_RFC	0.7735	4096	2	0.3060124	0.0166
nr-ahr_FEST	0.8915	256	3	nr-ahr_FEST	0.9083	4096	1	0.132503074	0.0168
nr-er_RFC	0.7566	128	3	nr-er_RFC	0.7735	4096	2	0.297437025	0.0169
nr-ahr_FEST	0.8907	128	2	nr-ahr_FEST	0.9077	4096	3	0.129086794	0.017
sr-mmp_FEST	0.8702	256	3	sr-mmp_FEST	0.8872	4096	3	0.116887281	0.017
nr-er_FEST	0.7646	256	2	nr-er_FEST	0.7816	4096	2	0.289720456	0.017
nr-er_RFC	0.7596	128	1	nr-er_RFC	0.7771	4096	3	0.278659559	0.0175
sr-mmp_RFC	0.8709	128	1	sr-mmp_RFC	0.8885	4096	2	0.103323814	0.0176
nr-ahr_FEST	0.8907	128	2	nr-ahr_FEST	0.9083	4096	1	0.115640667	0.0176
nr-er_FEST	0.7638	256	1	nr-er_FEST	0.7816	4096	2	0.267855573	0.0178
nr-ahr_RFC	0.8922	256	3	nr-ahr_RFC	0.91	4096	2	0.109114445	0.0178
sr-mmp_RFC	0.8706	256	3	sr-mmp_RFC	0.8885	4096	2	0.09774522	0.0179
sr-mmp_SVM	0.8696	256	2	sr-mmp_SVM	0.8877	4096	1	0.09506956	0.0181
nr-ahr_SVM	0.8885	128	1	nr-ahr_SVM	0.9067	4096	1	0.106502622	0.0182
nr-ahr_SVM	0.8864	128	2	nr-ahr_SVM	0.9047	4096	3	0.107527322	0.0183
nr-er_SVM	0.7546	128	2	nr-er_SVM	0.773	4096	2	0.257306814	0.0184
nr-ahr_RFC	0.8916	128	2	nr-ahr_RFC	0.91	4096	2	0.098104284	0.0184
sr-mmp_RFC	0.8645	128	2	sr-mmp_RFC	0.883	4096	1	0.093240703	0.0185
nr-ahr_SVM	0.8885	128	1	nr-ahr_SVM	0.907	4096	2	0.100636444	0.0185
nr-ahr_FEST	0.8915	256	3	nr-ahr_FEST	0.9104	4096	2	0.089089128	0.0189
sr-mmp_FEST	0.8657	128	2	sr-mmp_FEST	0.8846	4096	2	0.08493396	0.0189
sr-mmp_SVM	0.8682	128	1	sr-mmp_SVM	0.8877	4096	1	0.072778897	0.0195
nr-ahr_FEST	0.8907	128	2	nr-ahr_FEST	0.9104	4096	2	0.076854161	0.0197
sr-mmp_SVM	0.8696	256	2	sr-mmp_SVM	0.8896	4096	3	0.064214301	0.02
nr-er_SVM	0.7407	128	3	nr-er_SVM	0.7607	4096	1	0.22502541	0.02
nr-er_RFC	0.7569	128	2	nr-er_RFC	0.7771	4096	3	0.211842468	0.0202
nr-er_SVM	0.7527	256	3	nr-er_SVM	0.773	4096	2	0.211875037	0.0203
nr-ahr_SVM	0.8864	128	2	nr-ahr_SVM	0.9067	4096	1	0.072998937	0.0203
nr-er_RFC	0.7566	128	3	nr-er_RFC	0.7771	4096	3	0.205212939	0.0205
nr-er_FEST	0.7589	128	2	nr-er_FEST	0.7795	4096	3	0.20169323	0.0206
nr-ahr_SVM	0.8864	128	2	nr-ahr_SVM	0.907	4096	2	0.068683982	0.0206
nr-ahr_SVM	0.884	256	3	nr-ahr_SVM	0.9047	4096	3	0.069985241	0.0207
nr-er_FEST	0.7573	128	3	nr-er_FEST	0.7795	4096	3	0.169248252	0.0222

nr-er_FEST	0.7589	128	2	nr-er_FEST	0.7816	4096	2	0.158869803	0.0227
nr-er_FEST	0.7565	128	1	nr-er_FEST	0.7795	4096	3	0.154590965	0.023
nr-er_SVM	0.7407	128	3	nr-er_SVM	0.7649	4096	3	0.141120732	0.0242
nr-er_FEST	0.7573	128	3	nr-er_FEST	0.7816	4096	2	0.131868017	0.0243
nr-er_FEST	0.7565	128	1	nr-er_FEST	0.7816	4096	2	0.11978885	0.0251
nr-er_RFC	0.777	1024	2	nr-er_RFC	0.7639	2048	1	0.416224716	-0.0131
nr-er_FEST	0.779	1024	2	nr-er_FEST	0.7668	2048	1	0.447555679	-0.0122
nr-er_RFC	0.7722	1024	3	nr-er_RFC	0.7639	2048	1	0.607600738	-0.0083
cas-N6512_FEST	0.8878	1024	1	cas-N6512_FEST	0.8799	2048	3	0.179378705	-0.0079
cas-N6512_SVM	0.7694	1024	2	cas-N6512_SVM	0.7616	2048	1	0.341128016	-0.0078
nr-er_SVM	0.7694	1024	2	nr-er_SVM	0.7616	2048	1	0.630476312	-0.0078
cas-N6512_FEST	0.8876	2048	1	cas-N6512_FEST	0.8799	2048	3	0.190834003	-0.0077
nr-er_RFC	0.777	1024	2	nr-er_RFC	0.7699	2048	3	0.658290077	-0.0071
nr-ahr_SVM	0.908	2048	2	nr-ahr_SVM	0.9023	2048	3	0.601618884	-0.0057
nr-er_FEST	0.7723	1024	3	nr-er_FEST	0.7668	2048	1	0.733180978	-0.0055
nr-ahr_SVM	0.9077	2048	1	nr-ahr_SVM	0.9023	2048	3	0.621124996	-0.0054
cas-N6512_FEST	0.8852	1024	2	cas-N6512_FEST	0.8799	2048	3	0.370368026	-0.0053
cas-N6512_FEST	0.8852	2048	2	cas-N6512_FEST	0.8799	2048	3	0.370368026	-0.0053
cas-N6512_SVM	0.7725	2048	2	cas-N6512_SVM	0.7673	2048	3	0.522315914	-0.0052
nr-er_SVM	0.7725	2048	2	nr-er_SVM	0.7673	2048	3	0.747116513	-0.0052
nr-er_RFC	0.777	1024	2	nr-er_RFC	0.7725	2048	2	0.778876307	-0.0045
cas-N6512_RFC	0.8852	2048	2	cas-N6512_RFC	0.8811	2048	3	0.487223074	-0.0041
nr-er_FEST	0.779	1024	2	nr-er_FEST	0.775	2048	3	0.802331426	-0.004
nr-er_FEST	0.779	2048	2	nr-er_FEST	0.775	2048	3	0.802331426	-0.004
nr-ahr_RFC	0.9096	2048	2	nr-ahr_RFC	0.9061	2048	3	0.745495946	-0.0035
nr-ahr_SVM	0.905	1024	1	nr-ahr_SVM	0.9023	2048	3	0.805974673	-0.0027
nr-ahr_FEST	0.9095	2048	2	nr-ahr_FEST	0.9068	2048	3	0.802011415	-0.0027
cas-N6512_FEST	0.8878	1024	1	cas-N6512_FEST	0.8852	2048	2	0.654875058	-0.0026
nr-er_RFC	0.7725	2048	2	nr-er_RFC	0.7699	2048	3	0.871711998	-0.0026
cas-N6512_RFC	0.8836	2048	1	cas-N6512_RFC	0.8811	2048	3	0.672894568	-0.0025
nr-er_RFC	0.7664	1024	1	nr-er_RFC	0.7639	2048	1	0.877522161	-0.0025
cas-N6512_FEST	0.8876	2048	1	cas-N6512_FEST	0.8852	2048	2	0.680021457	-0.0024
cas-N6512_RFC	0.8834	1024	2	cas-N6512_RFC	0.8811	2048	3	0.697838414	-0.0023

sr-mmp_SVM	0.888	2048	2	sr-mmp_SVM	0.8857	2048	3	0.82719749	-0.0023	
nr-er_RFC	0.7722	1024	3	nr-er_RFC	0.7699	2048	3	0.886429264	-0.0023	
nr-ahr_RFC	0.9083	2048	1	nr-ahr_RFC	0.9061	2048	3	0.838821494	-0.0022	
cas-N6512_SVM	0.7694	1024	2	cas-N6512_SVM	0.7673	2048	3	0.796695802	-0.0021	
sr-mmp_RFC	0.8841	1024	2	sr-mmp_RFC	0.882	2048	1	0.844162396	-0.0021	
sr-mmp_RFC	0.8841	1024	3	sr-mmp_RFC	0.882	2048	1	0.844162396	-0.0021	
sr-mmp_FEST	0.8828	1024	2	sr-mmp_FEST	0.8807	2048	1	0.844870451	-0.0021	
nr-er_SVM	0.7694	1024	2	nr-er_SVM	0.7673	2048	3	0.896596504	-0.0021	
nr-ahr_FEST	0.9088	2048	1	nr-ahr_FEST	0.9068	2048	3	0.852892027	-0.002	
sr-mmp_SVM	0.8857	1024	1	sr-mmp_SVM	0.8838	2048	1	0.857981669	-0.0019	
sr-mmp_RFC	0.8859	2048	2	sr-mmp_RFC	0.8841	2048	3	0.865261631	-0.0018	
nr-ahr_FEST	0.9086	1024	2	nr-ahr_FEST	0.9068	2048	3	0.867521072	-0.0018	
nr-ahr_RFC	0.9076	1024	2	nr-ahr_RFC	0.9061	2048	3	0.889879967	-0.0015	
nr-ahr_RFC	0.9074	1024	1	nr-ahr_RFC	0.9061	2048	3	0.904531127	-0.0013	
cas-N6512_RFC	0.8822	1024	1	cas-N6512_RFC	0.8811	2048	3	0.853064162	-0.0011	
nr-ahr_SVM	0.9031	1024	2	nr-ahr_SVM	0.9023	2048	3	0.942228004	-0.0008	
sr-mmp_FEST	0.8832	2048	2	sr-mmp_FEST	0.8825	2048	3	0.947794535	-0.0007	
sr-mmp_RFC	0.8826	1024	1	sr-mmp_RFC	0.882	2048	1	0.955331335	-0.0006	
sr-mmp_FEST	0.8828	1024	2	sr-mmp_FEST	0.8825	2048	3	0.977629176	-0.0003	
cas-N6512_FEST	0.8878	1024	1	cas-N6512_FEST	0.8876	2048	1	0.972424727	-0.0002	
nr-er_FEST	0.767	1024	1	nr-er_FEST	0.7668	2048	1	0.990142347	-0.0002	
cas-N6512_FEST	0.8852	1024	2	cas-N6512_FEST	0.8852	2048	2	1	0	
cas-N6512_SVM	0.7616	1024	1	cas-N6512_SVM	0.7616	2048	1	1	0	
nr-er_FEST	0.779	1024	2	nr-er_FEST	0.779	2048	2	1	0	
nr-er_SVM	0.7616	1024	1	nr-er_SVM	0.7616	2048	1	1	0	
sr-mmp_RFC	0.8841	1024	2	sr-mmp_RFC	0.8841	2048	3	1	0	
sr-mmp_RFC	0.8841	1024	3	sr-mmp_RFC	0.8841	2048	3	1	0	
sr-mmp_SVM	0.8857	1024	1	sr-mmp_SVM	0.8857	2048	3	1	0	
sr-mmp_FEST	0.8806	1024	3	sr-mmp_FEST	0.8807	2048	1	0.992594523	1E-04	
cas-N6512_RFC	0.8834	1024	2	cas-N6512_RFC	0.8836	2048	1	0.97292528	0.0002	
nr-ahr_FEST	0.9086	1024	2	nr-ahr_FEST	0.9088	2048	1	0.985142884	0.0002	
nr-ahr_SVM	0.9077	2048	1	nr-ahr_SVM	0.908	2048	2	0.977805238	0.0003	
nr-er_RFC	0.7722	1024	3	nr-er_RFC	0.7725	2048	2	0.985112312	0.0003	

sr-mmp_FEST	0.8828	1024	2	sr-mmp_FEST	0.8832	2048	2	0.970138043	0.0004
sr-mmp_SVM	0.8833	1024	2	sr-mmp_SVM	0.8838	2048	1	0.962603901	0.0005
nr-ahr_FEST	0.9088	2048	1	nr-ahr_FEST	0.9095	2048	2	0.947922289	0.0007
cas-N6512_SVM	0.7609	1024	3	cas-N6512_SVM	0.7616	2048	1	0.932437596	0.0007
nr-ahr_RFC	0.9076	1024	2	nr-ahr_RFC	0.9083	2048	1	0.948217429	0.0007
nr-er_SVM	0.7609	1024	3	nr-er_SVM	0.7616	2048	1	0.965740538	0.0007
nr-ahr_FEST	0.9086	1024	2	nr-ahr_FEST	0.9095	2048	2	0.933105786	0.0009
nr-ahr_RFC	0.9074	1024	1	nr-ahr_RFC	0.9083	2048	1	0.933484064	0.0009
nr-ahr_RFC	0.9083	2048	1	nr-ahr_RFC	0.9096	2048	2	0.903543523	0.0013
cas-N6512_RFC	0.8822	1024	1	cas-N6512_RFC	0.8836	2048	1	0.812678538	0.0014
sr-mmp_RFC	0.8826	1024	1	sr-mmp_RFC	0.8841	2048	3	0.888218499	0.0015
cas-N6512_RFC	0.8836	2048	1	cas-N6512_RFC	0.8852	2048	2	0.785182871	0.0016
nr-ahr_FEST	0.9052	1024	1	nr-ahr_FEST	0.9068	2048	3	0.883046561	0.0016
sr-mmp_FEST	0.8807	2048	1	sr-mmp_FEST	0.8825	2048	3	0.86687751	0.0018
cas-N6512_RFC	0.8834	1024	2	cas-N6512_RFC	0.8852	2048	2	0.759215969	0.0018
sr-mmp_RFC	0.8841	1024	2	sr-mmp_RFC	0.8859	2048	2	0.865261631	0.0018
sr-mmp_RFC	0.8841	1024	3	sr-mmp_RFC	0.8859	2048	2	0.865261631	0.0018
sr-mmp_FEST	0.8806	1024	3	sr-mmp_FEST	0.8825	2048	3	0.859580981	0.0019
sr-mmp_SVM	0.8838	2048	1	sr-mmp_SVM	0.8857	2048	3	0.857981669	0.0019
nr-ahr_RFC	0.9076	1024	2	nr-ahr_RFC	0.9096	2048	2	0.852341689	0.002
sr-mmp_RFC	0.882	2048	1	sr-mmp_RFC	0.8841	2048	3	0.844162396	0.0021
nr-ahr_RFC	0.9074	1024	1	nr-ahr_RFC	0.9096	2048	2	0.837847218	0.0022
sr-mmp_SVM	0.8857	1024	1	sr-mmp_SVM	0.888	2048	2	0.82719749	0.0023
cas-N6512_FEST	0.8852	1024	2	cas-N6512_FEST	0.8876	2048	1	0.680021457	0.0024
cas-N6512_FEST	0.8775	1024	3	cas-N6512_FEST	0.8799	2048	3	0.68976051	0.0024
sr-mmp_SVM	0.8833	1024	2	sr-mmp_SVM	0.8857	2048	3	0.821333427	0.0024
sr-mmp_FEST	0.8807	2048	1	sr-mmp_FEST	0.8832	2048	2	0.81568169	0.0025
sr-mmp_FEST	0.8806	1024	3	sr-mmp_FEST	0.8832	2048	2	0.808482482	0.0026
nr-ahr_SVM	0.905	1024	1	nr-ahr_SVM	0.9077	2048	1	0.803632968	0.0027
nr-er_FEST	0.7723	1024	3	nr-er_FEST	0.775	2048	3	0.866405879	0.0027
nr-ahr_SVM	0.905	1024	1	nr-ahr_SVM	0.908	2048	2	0.782188575	0.003
cas-N6512_RFC	0.8822	1024	1	cas-N6512_RFC	0.8852	2048	2	0.610376451	0.003
cas-N6512_SVM	0.7694	1024	2	cas-N6512_SVM	0.7725	2048	2	0.702346437	0.0031

sr-mmp_FEST	0.8776	1024	1	sr-mmp_FEST	0.8807	2048	1	0.77468979	0.0031	
nr-er_SVM	0.7694	1024	2	nr-er_SVM	0.7725	2048	2	0.847367543	0.0031	
sr-mmp_SVM	0.8806	1024	3	sr-mmp_SVM	0.8838	2048	1	0.765216817	0.0032	
sr-mmp_RFC	0.8826	1024	1	sr-mmp_RFC	0.8859	2048	2	0.756379841	0.0033	
nr-er_RFC	0.7664	1024	1	nr-er_RFC	0.7699	2048	3	0.828557823	0.0035	
nr-ahr_FEST	0.9052	1024	1	nr-ahr_FEST	0.9088	2048	1	0.739485943	0.0036	
sr-mmp_RFC	0.882	2048	1	sr-mmp_RFC	0.8859	2048	2	0.714178217	0.0039	
nr-ahr_FEST	0.9029	1024	3	nr-ahr_FEST	0.9068	2048	3	0.721308077	0.0039	
sr-mmp_SVM	0.8838	2048	1	sr-mmp_SVM	0.888	2048	2	0.691191673	0.0042	
nr-ahr_FEST	0.9052	1024	1	nr-ahr_FEST	0.9095	2048	2	0.690743461	0.0043	
nr-er_RFC	0.7596	128	1	nr-er_RFC	0.7639	2048	1	0.791777559	0.0043	
cas-N6512_RFC	0.8766	1024	3	cas-N6512_RFC	0.8811	2048	3	0.453901651	0.0045	
nr-ahr_SVM	0.9031	1024	2	nr-ahr_SVM	0.9077	2048	1	0.673175898	0.0046	
sr-mmp_SVM	0.8833	1024	2	sr-mmp_SVM	0.888	2048	2	0.656953571	0.0047	
nr-ahr_SVM	0.9031	1024	2	nr-ahr_SVM	0.908	2048	2	0.652990566	0.0049	
sr-mmp_FEST	0.8776	1024	1	sr-mmp_FEST	0.8825	2048	3	0.64992356	0.0049	
nr-ahr_RFC	0.9012	1024	3	nr-ahr_RFC	0.9061	2048	3	0.655761042	0.0049	
sr-mmp_SVM	0.8806	1024	3	sr-mmp_SVM	0.8857	2048	3	0.632951728	0.0051	
cas-N6512_SVM	0.7565	128	1	cas-N6512_SVM	0.7616	2048	1	0.538357304	0.0051	
nr-er_SVM	0.7565	128	1	nr-er_SVM	0.7616	2048	1	0.754931668	0.0051	
sr-mmp_FEST	0.8776	1024	1	sr-mmp_FEST	0.8832	2048	2	0.603521996	0.0056	
cas-N6512_SVM	0.7616	1024	1	cas-N6512_SVM	0.7673	2048	3	0.48748535	0.0057	
cas-N6512_SVM	0.7616	2048	1	cas-N6512_SVM	0.7673	2048	3	0.48748535	0.0057	
nr-er_SVM	0.7616	1024	1	nr-er_SVM	0.7673	2048	3	0.72552348	0.0057	
nr-er_SVM	0.7616	2048	1	nr-er_SVM	0.7673	2048	3	0.72552348	0.0057	
cas-N6512_FEST	0.8741	128	1	cas-N6512_FEST	0.8799	2048	3	0.338035047	0.0058	
nr-ahr_FEST	0.9029	1024	3	nr-ahr_FEST	0.9088	2048	1	0.58773334	0.0059	
nr-er_RFC	0.7639	2048	1	nr-er_RFC	0.7699	2048	3	0.710885102	0.006	
nr-er_RFC	0.7664	1024	1	nr-er_RFC	0.7725	2048	2	0.705400105	0.0061	
cas-N6512_SVM	0.7609	1024	3	cas-N6512_SVM	0.7673	2048	3	0.435925253	0.0064	
nr-er_SVM	0.7609	1024	3	nr-er_SVM	0.7673	2048	3	0.69355152	0.0064	
nr-ahr_FEST	0.9029	1024	3	nr-ahr_FEST	0.9095	2048	2	0.543566008	0.0066	
nr-er_FEST	0.7723	1024	3	nr-er_FEST	0.779	2048	2	0.675535656	0.0067	

cas-N6512_RFC	0.8766	1024	3	cas-N6512_RFC	0.8836	2048	1	0.241586509	0.007
cas-N6512_SVM	0.7546	128	2	cas-N6512_SVM	0.7616	2048	1	0.399147849	0.007
nr-er_RFC	0.7569	128	2	nr-er_RFC	0.7639	2048	1	0.667844245	0.007
nr-er_SVM	0.7546	128	2	nr-er_SVM	0.7616	2048	1	0.668667937	0.007
nr-ahr_RFC	0.9012	1024	3	nr-ahr_RFC	0.9083	2048	1	0.516243512	0.0071
nr-er_RFC	0.7566	128	3	nr-er_RFC	0.7639	2048	1	0.654569756	0.0073
sr-mmp_SVM	0.8806	1024	3	sr-mmp_SVM	0.888	2048	2	0.486517789	0.0074
cas-N6512_FEST	0.8775	1024	3	cas-N6512_FEST	0.8852	2048	2	0.195364738	0.0077
nr-ahr_RFC	0.8983	128	1	nr-ahr_RFC	0.9061	2048	3	0.480739801	0.0078
nr-er_FEST	0.7589	128	2	nr-er_FEST	0.7668	2048	1	0.627194879	0.0079
nr-er_FEST	0.767	1024	1	nr-er_FEST	0.775	2048	3	0.619346522	0.008
nr-er_FEST	0.7668	2048	1	nr-er_FEST	0.775	2048	3	0.610659051	0.0082
nr-ahr_RFC	0.9012	1024	3	nr-ahr_RFC	0.9096	2048	2	0.441119612	0.0084
cas-N6512_RFC	0.8766	1024	3	cas-N6512_RFC	0.8852	2048	2	0.148877642	0.0086
nr-er_RFC	0.7639	2048	1	nr-er_RFC	0.7725	2048	2	0.594612987	0.0086
nr-ahr_SVM	0.8935	1024	3	nr-ahr_SVM	0.9023	2048	3	0.434764897	0.0088
sr-mmp_FEST	0.8715	128	1	sr-mmp_FEST	0.8807	2048	1	0.400408524	0.0092
nr-er_FEST	0.7573	128	3	nr-er_FEST	0.7668	2048	1	0.559551525	0.0095
nr-ahr_RFC	0.8983	128	1	nr-ahr_RFC	0.9083	2048	1	0.363637457	0.01
cas-N6512_FEST	0.8775	1024	3	cas-N6512_FEST	0.8876	2048	1	0.087801333	0.0101
nr-er_RFC	0.7596	128	1	nr-er_RFC	0.7699	2048	3	0.525628941	0.0103
nr-er_FEST	0.7565	128	1	nr-er_FEST	0.7668	2048	1	0.527153367	0.0103
cas-N6512_SVM	0.7565	128	1	cas-N6512_SVM	0.7673	2048	3	0.190326241	0.0108
nr-er_SVM	0.7565	128	1	nr-er_SVM	0.7673	2048	3	0.507166872	0.0108
cas-N6512_SVM	0.7616	1024	1	cas-N6512_SVM	0.7725	2048	2	0.18217371	0.0109
cas-N6512_SVM	0.7616	2048	1	cas-N6512_SVM	0.7725	2048	2	0.18217371	0.0109
nr-er_SVM	0.7616	1024	1	nr-er_SVM	0.7725	2048	2	0.500593234	0.0109
nr-er_SVM	0.7616	2048	1	nr-er_SVM	0.7725	2048	2	0.500593234	0.0109
sr-mmp_FEST	0.8715	128	1	sr-mmp_FEST	0.8825	2048	3	0.313216634	0.011
sr-mmp_RFC	0.8709	128	1	sr-mmp_RFC	0.882	2048	1	0.30972669	0.0111
cas-N6512_FEST	0.8741	128	1	cas-N6512_FEST	0.8852	2048	2	0.063807797	0.0111
nr-ahr_RFC	0.8983	128	1	nr-ahr_RFC	0.9096	2048	2	0.303202616	0.0113
cas-N6512_SVM	0.7609	1024	3	cas-N6512_SVM	0.7725	2048	2	0.1559368	0.0116

nr-er_SVM	0.7609	1024	3	nr-er_SVM	0.7725	2048	2	0.473673201	0.0116
sr-mmp_FEST	0.8715	128	1	sr-mmp_FEST	0.8832	2048	2	0.282840217	0.0117
nr-ahr_FEST	0.895	128	1	nr-ahr_FEST	0.9068	2048	3	0.288756249	0.0118
nr-er_FEST	0.767	1024	1	nr-er_FEST	0.779	2048	2	0.454979778	0.012
nr-er_FEST	0.7668	2048	1	nr-er_FEST	0.779	2048	2	0.447555679	0.0122
cas-N6512_SVM	0.7546	128	2	cas-N6512_SVM	0.7673	2048	3	0.124180421	0.0127
nr-er_SVM	0.7546	128	2	nr-er_SVM	0.7673	2048	3	0.435917652	0.0127
nr-er_RFC	0.7596	128	1	nr-er_RFC	0.7725	2048	2	0.425912445	0.0129
nr-er_RFC	0.7569	128	2	nr-er_RFC	0.7699	2048	3	0.423814349	0.013
sr-mmp_RFC	0.8709	128	1	sr-mmp_RFC	0.8841	2048	3	0.225386659	0.0132
nr-er_RFC	0.7566	128	3	nr-er_RFC	0.7699	2048	3	0.413277968	0.0133
nr-ahr_FEST	0.895	128	1	nr-ahr_FEST	0.9088	2048	1	0.212699468	0.0138
nr-ahr_SVM	0.8885	128	1	nr-ahr_SVM	0.9023	2048	3	0.225281774	0.0138
nr-ahr_SVM	0.8935	1024	3	nr-ahr_SVM	0.9077	2048	1	0.202266257	0.0142
nr-ahr_SVM	0.8935	1024	3	nr-ahr_SVM	0.908	2048	2	0.192601279	0.0145
nr-ahr_FEST	0.895	128	1	nr-ahr_FEST	0.9095	2048	2	0.189707374	0.0145
nr-ahr_RFC	0.8916	128	2	nr-ahr_RFC	0.9061	2048	3	0.196210835	0.0145
sr-mmp_RFC	0.8709	128	1	sr-mmp_RFC	0.8859	2048	2	0.16698413	0.015
sr-mmp_FEST	0.8657	128	2	sr-mmp_FEST	0.8807	2048	1	0.174358808	0.015
nr-er_RFC	0.7569	128	2	nr-er_RFC	0.7725	2048	2	0.336376075	0.0156
sr-mmp_SVM	0.8682	128	1	sr-mmp_SVM	0.8838	2048	1	0.15394917	0.0156
nr-er_RFC	0.7566	128	3	nr-er_RFC	0.7725	2048	2	0.327249186	0.0159
nr-ahr_SVM	0.8864	128	2	nr-ahr_SVM	0.9023	2048	3	0.164104025	0.0159
nr-er_SVM	0.7565	128	1	nr-er_SVM	0.7725	2048	2	0.324243604	0.016
cas-N6512_SVM	0.7565	128	1	cas-N6512_SVM	0.7725	2048	2	0.051249213	0.016
nr-ahr_FEST	0.8907	128	2	nr-ahr_FEST	0.9068	2048	3	0.1514126	0.0161
nr-er_FEST	0.7589	128	2	nr-er_FEST	0.775	2048	3	0.319785458	0.0161
nr-ahr_RFC	0.8916	128	2	nr-ahr_RFC	0.9083	2048	1	0.134742361	0.0167
sr-mmp_FEST	0.8657	128	2	sr-mmp_FEST	0.8825	2048	3	0.127033321	0.0168
sr-mmp_FEST	0.8657	128	2	sr-mmp_FEST	0.8832	2048	2	0.111528966	0.0175
sr-mmp_RFC	0.8645	128	2	sr-mmp_RFC	0.882	2048	1	0.112929888	0.0175
sr-mmp_SVM	0.8682	128	1	sr-mmp_SVM	0.8857	2048	3	0.10858291	0.0175
nr-er_FEST	0.7573	128	3	nr-er_FEST	0.775	2048	3	0.274500629	0.0177

nr-er_SVM	0.7546	128	2	nr-er_SVM	0.7725	2048	2	0.270614174	0.0179
nr-ahr_RFC	0.8916	128	2	nr-ahr_RFC	0.9096	2048	2	0.105939048	0.018
nr-ahr_FEST	0.8907	128	2	nr-ahr_FEST	0.9088	2048	1	0.105273453	0.0181
nr-er_FEST	0.7565	128	1	nr-er_FEST	0.775	2048	3	0.253611089	0.0185
nr-ahr_FEST	0.8907	128	2	nr-ahr_FEST	0.9095	2048	2	0.091979428	0.0188
nr-ahr_SVM	0.8885	128	1	nr-ahr_SVM	0.9077	2048	1	0.087921479	0.0192
nr-ahr_SVM	0.8885	128	1	nr-ahr_SVM	0.908	2048	2	0.082873354	0.0195
sr-mmp_RFC	0.8645	128	2	sr-mmp_RFC	0.8841	2048	3	0.074806683	0.0196
sr-mmp_SVM	0.8682	128	1	sr-mmp_SVM	0.888	2048	2	0.068337099	0.0198
nr-er_FEST	0.7589	128	2	nr-er_FEST	0.779	2048	2	0.213007275	0.0201
nr-er_SVM	0.7407	128	3	nr-er_SVM	0.7616	2048	1	0.204611494	0.0209
nr-ahr_SVM	0.8864	128	2	nr-ahr_SVM	0.9077	2048	1	0.059407214	0.0213
sr-mmp_RFC	0.8645	128	2	sr-mmp_RFC	0.8859	2048	2	0.051029382	0.0214
nr-ahr_SVM	0.8864	128	2	nr-ahr_SVM	0.908	2048	2	0.055754714	0.0216
nr-er_FEST	0.7573	128	3	nr-er_FEST	0.779	2048	2	0.179201751	0.0217
nr-er_FEST	0.7565	128	1	nr-er_FEST	0.779	2048	2	0.163894199	0.0225
nr-er_SVM	0.7407	128	3	nr-er_SVM	0.7673	2048	3	0.105258167	0.0266
nr-er_SVM	0.7407	128	3	nr-er_SVM	0.7725	2048	2	0.052052933	0.0318
nr-ahr_SVM	0.905	1024	1	nr-ahr_SVM	0.8935	1024	3	0.304604735	-0.0115
cas-N6512_FEST	0.8878	1024	1	cas-N6512_FEST	0.8775	1024	3	0.0815685	-0.0103
nr-ahr_SVM	0.9031	1024	2	nr-ahr_SVM	0.8935	1024	3	0.393373534	-0.0096
cas-N6512_SVM	0.7694	1024	2	cas-N6512_SVM	0.7609	1024	3	0.299867482	-0.0085
nr-er_SVM	0.7694	1024	2	nr-er_SVM	0.7609	1024	3	0.600270393	-0.0085
cas-N6512_FEST	0.8852	1024	2	cas-N6512_FEST	0.8775	1024	3	0.195364738	-0.0077
cas-N6512_RFC	0.8834	1024	2	cas-N6512_RFC	0.8766	1024	3	0.255496584	-0.0068
nr-er_FEST	0.779	1024	2	nr-er_FEST	0.7723	1024	3	0.675535656	-0.0067
nr-ahr_RFC	0.9076	1024	2	nr-ahr_RFC	0.9012	1024	3	0.559077178	-0.0064
nr-ahr_RFC	0.9074	1024	1	nr-ahr_RFC	0.9012	1024	3	0.571598492	-0.0062
nr-ahr_FEST	0.9086	1024	2	nr-ahr_FEST	0.9029	1024	3	0.600623293	-0.0057
cas-N6512_RFC	0.8822	1024	1	cas-N6512_RFC	0.8766	1024	3	0.350246869	-0.0056
sr-mmp_SVM	0.8857	1024	1	sr-mmp_SVM	0.8806	1024	3	0.632951728	-0.0051
nr-er_RFC	0.777	1024	2	nr-er_RFC	0.7722	1024	3	0.764600947	-0.0048
sr-mmp_SVM	0.8833	1024	2	sr-mmp_SVM	0.8806	1024	3	0.801233281	-0.0027

cas-N6512_FEST	0.8878	1024	1	cas-N6512_FEST	0.8852	1024	2	0.654875058	-0.0026
sr-mmp_SVM	0.8857	1024	1	sr-mmp_SVM	0.8833	1024	2	0.821333427	-0.0024
nr-ahr_FEST	0.9052	1024	1	nr-ahr_FEST	0.9029	1024	3	0.833974175	-0.0023
sr-mmp_FEST	0.8828	1024	2	sr-mmp_FEST	0.8806	1024	3	0.83761195	-0.0022
nr-ahr_SVM	0.905	1024	1	nr-ahr_SVM	0.9031	1024	2	0.862531025	-0.0019
cas-N6512_SVM	0.7616	1024	1	cas-N6512_SVM	0.7609	1024	3	0.932437596	-0.0007
nr-er_SVM	0.7616	1024	1	nr-er_SVM	0.7609	1024	3	0.965740538	-0.0007
sr-mmp_RFC	0.8841	1024	2	sr-mmp_RFC	0.8841	1024	3	1	0
nr-ahr_RFC	0.9074	1024	1	nr-ahr_RFC	0.9076	1024	2	0.985226709	0.0002
cas-N6512_RFC	0.8822	1024	1	cas-N6512_RFC	0.8834	1024	2	0.839109764	0.0012
sr-mmp_RFC	0.8826	1024	1	sr-mmp_RFC	0.8841	1024	2	0.888218499	0.0015
sr-mmp_RFC	0.8826	1024	1	sr-mmp_RFC	0.8841	1024	3	0.888218499	0.0015
sr-mmp_FEST	0.8776	1024	1	sr-mmp_FEST	0.8806	1024	3	0.781807439	0.003
nr-ahr_FEST	0.9052	1024	1	nr-ahr_FEST	0.9086	1024	2	0.753586922	0.0034
sr-mmp_FEST	0.8776	1024	1	sr-mmp_FEST	0.8828	1024	2	0.62987075	0.0052
nr-er_FEST	0.767	1024	1	nr-er_FEST	0.7723	1024	3	0.742502243	0.0053
nr-er_RFC	0.7664	1024	1	nr-er_RFC	0.7722	1024	3	0.719310857	0.0058
cas-N6512_SVM	0.7616	1024	1	cas-N6512_SVM	0.7694	1024	2	0.341128016	0.0078
nr-er_SVM	0.7616	1024	1	nr-er_SVM	0.7694	1024	2	0.630476312	0.0078
nr-er_RFC	0.7664	1024	1	nr-er_RFC	0.777	1024	2	0.509993203	0.0106
nr-er_FEST	0.767	1024	1	nr-er_FEST	0.779	1024	2	0.454979778	0.012
nr-ahr_SVM	0.908	2048	2	nr-ahr_SVM	0.8863	512	3	0.054678497	-0.0217
nr-ahr_SVM	0.9077	2048	1	nr-ahr_SVM	0.8863	512	3	0.058272521	-0.0214
nr-ahr_SVM	0.907	4096	2	nr-ahr_SVM	0.8863	512	3	0.067404379	-0.0207
nr-ahr_SVM	0.9067	4096	1	nr-ahr_SVM	0.8863	512	3	0.071653572	-0.0204
sr-mmp_SVM	0.8896	4096	3	sr-mmp_SVM	0.8707	512	3	0.079753228	-0.0189
nr-ahr_SVM	0.905	1024	1	nr-ahr_SVM	0.8863	512	3	0.099908845	-0.0187
nr-ahr_SVM	0.9047	4096	3	nr-ahr_SVM	0.8863	512	3	0.105687458	-0.0184
sr-mmp_SVM	0.888	2048	2	sr-mmp_SVM	0.8707	512	3	0.109769997	-0.0173
sr-mmp_SVM	0.8877	4096	1	sr-mmp_SVM	0.8707	512	3	0.11626131	-0.017
nr-er_FEST	0.7816	4096	2	nr-er_FEST	0.7647	512	1	0.292537223	-0.0169
nr-ahr_SVM	0.9031	1024	2	nr-ahr_SVM	0.8863	512	3	0.140940742	-0.0168
sr-mmp_SVM	0.892	4096	2	sr-mmp_SVM	0.876	512	2	0.132736949	-0.016

nr-ahr_SVM	0.9023	2048	3	nr-ahr_SVM	0.8863	512	3	0.161549335	-0.016
sr-mmp_SVM	0.8857	1024	1	sr-mmp_SVM	0.8707	512	3	0.167272939	-0.015
sr-mmp_SVM	0.8857	2048	3	sr-mmp_SVM	0.8707	512	3	0.167272939	-0.015
nr-ahr_SVM	0.9012	512	1	nr-ahr_SVM	0.8863	512	3	0.193357849	-0.0149
nr-er_FEST	0.7795	4096	3	nr-er_FEST	0.7647	512	1	0.357327453	-0.0148
nr-er_FEST	0.779	1024	2	nr-er_FEST	0.7647	512	1	0.373963081	-0.0143
nr-er_FEST	0.779	2048	2	nr-er_FEST	0.7647	512	1	0.373963081	-0.0143
sr-mmp_SVM	0.8896	4096	3	sr-mmp_SVM	0.876	512	2	0.203281899	-0.0136
sr-mmp_SVM	0.8838	2048	1	sr-mmp_SVM	0.8707	512	3	0.229314524	-0.0131
nr-ahr_FEST	0.9104	4096	2	nr-ahr_FEST	0.8973	512	3	0.232805928	-0.0131
cas-N6512_SVM	0.773	4096	2	cas-N6512_SVM	0.7601	512	3	0.114685518	-0.0129
nr-er_SVM	0.773	4096	2	nr-er_SVM	0.7601	512	3	0.425629711	-0.0129
cas-N6512_SVM	0.773	4096	2	cas-N6512_SVM	0.7602	512	1	0.11749135	-0.0128
nr-er_SVM	0.773	4096	2	nr-er_SVM	0.7602	512	1	0.429199085	-0.0128
sr-mmp_SVM	0.8834	512	1	sr-mmp_SVM	0.8707	512	3	0.244173158	-0.0127
nr-ahr_SVM	0.8989	256	1	nr-ahr_SVM	0.8863	512	3	0.273589315	-0.0126
sr-mmp_SVM	0.8833	1024	2	sr-mmp_SVM	0.8707	512	3	0.24798777	-0.0126
cas-N6512_SVM	0.7725	2048	2	cas-N6512_SVM	0.7601	512	3	0.129612514	-0.0124
nr-er_SVM	0.7725	2048	2	nr-er_SVM	0.7601	512	3	0.443932581	-0.0124
cas-N6512_SVM	0.7725	2048	2	cas-N6512_SVM	0.7602	512	1	0.132698826	-0.0123
nr-er_SVM	0.7725	2048	2	nr-er_SVM	0.7602	512	1	0.44758938	-0.0123
nr-ahr_FEST	0.9095	2048	2	nr-ahr_FEST	0.8973	512	3	0.267467937	-0.0122
nr-er_RFC	0.7771	4096	3	nr-er_RFC	0.765	512	1	0.452362445	-0.0121
sr-mmp_SVM	0.888	2048	2	sr-mmp_SVM	0.876	512	2	0.263001327	-0.012
nr-er_RFC	0.777	1024	2	nr-er_RFC	0.765	512	1	0.456137211	-0.012
nr-er_FEST	0.7816	4096	2	nr-er_FEST	0.7698	512	2	0.460947754	-0.0118
nr-er_FEST	0.7816	4096	2	nr-er_FEST	0.7698	512	3	0.460947754	-0.0118
sr-mmp_SVM	0.8877	4096	1	sr-mmp_SVM	0.876	512	2	0.275381865	-0.0117
nr-ahr_FEST	0.9088	2048	1	nr-ahr_FEST	0.8973	512	3	0.296656235	-0.0115
cas-N6512_RFC	0.8811	2048	3	cas-N6512_RFC	0.8697	512	3	0.061327609	-0.0114
nr-ahr_FEST	0.9086	1024	2	nr-ahr_FEST	0.8973	512	3	0.305354679	-0.0113
nr-ahr_SVM	0.908	2048	2	nr-ahr_SVM	0.8967	512	2	0.306633514	-0.0113
cas-N6512_RFC	0.8808	4096	3	cas-N6512_RFC	0.8697	512	3	0.068637916	-0.0111

nr-ahr_FEST	0.9083	4096	1	nr-ahr_FEST	0.8973	512	3	0.318700783	-0.011
nr-ahr_SVM	0.9077	2048	1	nr-ahr_SVM	0.8967	512	2	0.319977927	-0.011
sr-mmp_FEST	0.8872	4096	3	sr-mmp_FEST	0.8766	512	3	0.322986085	-0.0106
sr-mmp_RFC	0.8885	4096	2	sr-mmp_RFC	0.878	512	3	0.325251216	-0.0105
nr-ahr_SVM	0.8967	512	2	nr-ahr_SVM	0.8863	512	3	0.368261244	-0.0104
nr-ahr_FEST	0.9077	4096	3	nr-ahr_FEST	0.8973	512	3	0.346462059	-0.0104
cas-N6512_FEST	0.8811	4096	3	cas-N6512_FEST	0.8707	512	3	0.087195452	-0.0104
nr-ahr_RFC	0.91	4096	2	nr-ahr_RFC	0.8997	512	3	0.346066146	-0.0103
nr-ahr_SVM	0.907	4096	2	nr-ahr_SVM	0.8967	512	2	0.352492103	-0.0103
nr-er_FEST	0.775	2048	3	nr-er_FEST	0.7647	512	1	0.523021415	-0.0103
cas-N6512_RFC	0.8798	512	1	cas-N6512_RFC	0.8697	512	3	0.098235658	-0.0101
nr-ahr_SVM	0.9067	4096	1	nr-ahr_SVM	0.8967	512	2	0.367011572	-0.01
sr-mmp_SVM	0.8806	1024	3	sr-mmp_SVM	0.8707	512	3	0.366238961	-0.0099
nr-ahr_RFC	0.9096	2048	2	nr-ahr_RFC	0.8997	512	3	0.365559631	-0.0099
sr-mmp_SVM	0.8857	1024	1	sr-mmp_SVM	0.876	512	2	0.367550545	-0.0097
sr-mmp_SVM	0.8857	2048	3	sr-mmp_SVM	0.876	512	2	0.367550545	-0.0097
cas-N6512_FEST	0.8804	512	2	cas-N6512_FEST	0.8707	512	3	0.111154381	-0.0097
nr-er_FEST	0.7795	4096	3	nr-er_FEST	0.7698	512	2	0.545040136	-0.0097
nr-er_FEST	0.7795	4096	3	nr-er_FEST	0.7698	512	3	0.545040136	-0.0097
nr-ahr_FEST	0.9068	2048	3	nr-ahr_FEST	0.8973	512	3	0.390740421	-0.0095
cas-N6512_SVM	0.7694	1024	2	cas-N6512_SVM	0.7601	512	3	0.257012904	-0.0093
nr-er_SVM	0.7694	1024	2	nr-er_SVM	0.7601	512	3	0.56660317	-0.0093
cas-N6512_FEST	0.8799	2048	3	cas-N6512_FEST	0.8707	512	3	0.131195519	-0.0092
cas-N6512_SVM	0.7694	1024	2	cas-N6512_SVM	0.7602	512	1	0.262121127	-0.0092
nr-er_FEST	0.779	1024	2	nr-er_FEST	0.7698	512	2	0.566090213	-0.0092
nr-er_FEST	0.779	1024	2	nr-er_FEST	0.7698	512	3	0.566090213	-0.0092
nr-er_FEST	0.779	2048	2	nr-er_FEST	0.7698	512	2	0.566090213	-0.0092
nr-er_FEST	0.779	2048	2	nr-er_FEST	0.7698	512	3	0.566090213	-0.0092
nr-er_SVM	0.7694	1024	2	nr-er_SVM	0.7602	512	1	0.570760001	-0.0092
sr-mmp_FEST	0.8872	4096	3	sr-mmp_FEST	0.8781	512	1	0.394935661	-0.0091
cas-N6512_RFC	0.8787	512	2	cas-N6512_RFC	0.8697	512	3	0.141512255	-0.009
sr-mmp_RFC	0.8869	4096	3	sr-mmp_RFC	0.878	512	3	0.405743144	-0.0089
sr-mmp_SVM	0.892	4096	2	sr-mmp_SVM	0.8834	512	1	0.412827211	-0.0086

sr-mmp_RFC	0.8885	4096	2	sr-mmp_RFC	0.8799	512	1	0.418842477	-0.0086
nr-ahr_RFC	0.9083	2048	1	nr-ahr_RFC	0.8997	512	3	0.433230122	-0.0086
sr-mmp_SVM	0.8793	256	1	sr-mmp_SVM	0.8707	512	3	0.433518309	-0.0086
nr-ahr_FEST	0.9058	512	1	nr-ahr_FEST	0.8973	512	3	0.443538099	-0.0085
sr-mmp_FEST	0.8872	4096	3	sr-mmp_FEST	0.8787	512	2	0.426353145	-0.0085
nr-er_RFC	0.7735	4096	2	nr-er_RFC	0.765	512	1	0.598426965	-0.0085
nr-ahr_FEST	0.9057	512	2	nr-ahr_FEST	0.8973	512	3	0.449019374	-0.0084
nr-ahr_SVM	0.905	1024	1	nr-ahr_SVM	0.8967	512	2	0.455706692	-0.0083
nr-ahr_RFC	0.908	4096	1	nr-ahr_RFC	0.8997	512	3	0.449754846	-0.0083
cas-N6512_SVM	0.773	4096	2	cas-N6512_SVM	0.765	512	2	0.325780304	-0.008
sr-mmp_FEST	0.8846	4096	2	sr-mmp_FEST	0.8766	512	3	0.457802617	-0.008
nr-er_SVM	0.773	4096	2	nr-er_SVM	0.765	512	2	0.620229795	-0.008
nr-ahr_SVM	0.9047	4096	3	nr-ahr_SVM	0.8967	512	2	0.472447381	-0.008
cas-N6512_RFC	0.8776	256	1	cas-N6512_RFC	0.8697	512	3	0.197850056	-0.0079
sr-mmp_RFC	0.8859	2048	2	sr-mmp_RFC	0.878	512	3	0.461324897	-0.0079
nr-ahr_FEST	0.9052	1024	1	nr-ahr_FEST	0.8973	512	3	0.47695667	-0.0079
nr-ahr_RFC	0.9076	1024	2	nr-ahr_RFC	0.8997	512	3	0.472296522	-0.0079
sr-mmp_SVM	0.8838	2048	1	sr-mmp_SVM	0.876	512	2	0.470203036	-0.0078
nr-ahr_RFC	0.9074	1024	1	nr-ahr_RFC	0.8997	512	3	0.483780544	-0.0077
cas-N6512_FEST	0.888	4096	1	cas-N6512_FEST	0.8804	512	2	0.195794862	-0.0076
sr-mmp_RFC	0.8885	4096	2	sr-mmp_RFC	0.8809	512	2	0.474172721	-0.0076
nr-er_FEST	0.7723	1024	3	nr-er_FEST	0.7647	512	1	0.63803212	-0.0076
cas-N6512_SVM	0.7725	2048	2	cas-N6512_SVM	0.765	512	2	0.357154096	-0.0075
nr-er_RFC	0.7725	2048	2	nr-er_RFC	0.765	512	1	0.642356967	-0.0075
nr-er_SVM	0.7725	2048	2	nr-er_SVM	0.765	512	2	0.642356967	-0.0075
sr-mmp_SVM	0.8834	512	1	sr-mmp_SVM	0.876	512	2	0.493573495	-0.0074
cas-N6512_FEST	0.8878	1024	1	cas-N6512_FEST	0.8804	512	2	0.208016224	-0.0074
sr-mmp_SVM	0.8833	1024	2	sr-mmp_SVM	0.876	512	2	0.499506485	-0.0073
cas-N6512_FEST	0.8876	2048	1	cas-N6512_FEST	0.8804	512	2	0.220770119	-0.0072
cas-N6512_SVM	0.7673	2048	3	cas-N6512_SVM	0.7601	512	3	0.381109326	-0.0072
nr-ahr_SVM	0.8935	1024	3	nr-ahr_SVM	0.8863	512	3	0.535897586	-0.0072
nr-er_RFC	0.7722	1024	3	nr-er_RFC	0.765	512	1	0.655781895	-0.0072
nr-er_SVM	0.7673	2048	3	nr-er_SVM	0.7601	512	3	0.65770247	-0.0072

cas-N6512_SVM	0.7673	2048	3	cas-N6512_SVM	0.7602	512	1	0.387716239	-0.0071
nr-er_RFC	0.7771	4096	3	nr-er_RFC	0.77	512	3	0.658248457	-0.0071
nr-er_SVM	0.7673	2048	3	nr-er_SVM	0.7602	512	1	0.662141096	-0.0071
sr-mmp_RFC	0.8869	4096	3	sr-mmp_RFC	0.8799	512	1	0.511755369	-0.007
nr-er_RFC	0.777	1024	2	nr-er_RFC	0.77	512	3	0.662783241	-0.007
nr-ahr_RFC	0.9067	4096	3	nr-ahr_RFC	0.8997	512	3	0.525053187	-0.007
cas-N6512_RFC	0.8856	4096	2	cas-N6512_RFC	0.8787	512	2	0.244323981	-0.0069
cas-N6512_RFC	0.8766	1024	3	cas-N6512_RFC	0.8697	512	3	0.261650508	-0.0069
cas-N6512_FEST	0.8775	1024	3	cas-N6512_FEST	0.8707	512	3	0.26684792	-0.0068
nr-ahr_SVM	0.908	2048	2	nr-ahr_SVM	0.9012	512	1	0.534407498	-0.0068
sr-mmp_FEST	0.8832	2048	2	sr-mmp_FEST	0.8766	512	3	0.541177548	-0.0066
sr-mmp_FEST	0.8846	4096	2	sr-mmp_FEST	0.8781	512	1	0.545297912	-0.0065
cas-N6512_RFC	0.8852	2048	2	cas-N6512_RFC	0.8787	512	2	0.273154807	-0.0065
nr-ahr_SVM	0.9077	2048	1	nr-ahr_SVM	0.9012	512	1	0.552862556	-0.0065
nr-ahr_RFC	0.9061	2048	3	nr-ahr_RFC	0.8997	512	3	0.561700469	-0.0064
nr-ahr_SVM	0.9031	1024	2	nr-ahr_SVM	0.8967	512	2	0.566771633	-0.0064
nr-ahr_RFC	0.906	512	2	nr-ahr_RFC	0.8997	512	3	0.567916527	-0.0063
sr-mmp_SVM	0.8896	4096	3	sr-mmp_SVM	0.8834	512	1	0.55672039	-0.0062
sr-mmp_FEST	0.8828	1024	2	sr-mmp_FEST	0.8766	512	3	0.56623828	-0.0062
sr-mmp_RFC	0.8841	1024	2	sr-mmp_RFC	0.878	512	3	0.570713717	-0.0061
sr-mmp_RFC	0.8841	1024	3	sr-mmp_RFC	0.878	512	3	0.570713717	-0.0061
sr-mmp_RFC	0.8841	2048	3	sr-mmp_RFC	0.878	512	3	0.570713717	-0.0061
cas-N6512_FEST	0.8864	4096	2	cas-N6512_FEST	0.8804	512	2	0.308784931	-0.006
sr-mmp_RFC	0.8869	4096	3	sr-mmp_RFC	0.8809	512	2	0.573177331	-0.006
sr-mmp_RFC	0.8859	2048	2	sr-mmp_RFC	0.8799	512	1	0.57455115	-0.006
nr-ahr_SVM	0.8923	256	2	nr-ahr_SVM	0.8863	512	3	0.606780819	-0.006
nr-er_RFC	0.7771	4096	3	nr-er_RFC	0.7711	512	2	0.708355743	-0.006
sr-mmp_FEST	0.8846	4096	2	sr-mmp_FEST	0.8787	512	2	0.582618063	-0.0059
nr-er_RFC	0.777	1024	2	nr-er_RFC	0.7711	512	2	0.713017596	-0.0059
sr-mmp_FEST	0.8825	2048	3	sr-mmp_FEST	0.8766	512	3	0.585368128	-0.0059
cas-N6512_RFC	0.8856	4096	2	cas-N6512_RFC	0.8798	512	1	0.326632866	-0.0058
nr-ahr_SVM	0.907	4096	2	nr-ahr_SVM	0.9012	512	1	0.596998037	-0.0058
nr-ahr_RFC	0.9054	512	1	nr-ahr_RFC	0.8997	512	3	0.605826295	-0.0057

nr-ahr_FEST	0.9029	1024	3	nr-ahr_FEST	0.8973	512	3	0.615927779	-0.0056
nr-ahr_SVM	0.9023	2048	3	nr-ahr_SVM	0.8967	512	2	0.616828065	-0.0056
cas-N6512_FEST	0.886	512	1	cas-N6512_FEST	0.8804	512	2	0.342564418	-0.0056
nr-ahr_SVM	0.9067	4096	1	nr-ahr_SVM	0.9012	512	1	0.61634672	-0.0055
cas-N6512_RFC	0.8852	2048	2	cas-N6512_RFC	0.8798	512	1	0.361509373	-0.0054
sr-mmp_SVM	0.876	512	2	sr-mmp_SVM	0.8707	512	3	0.63119911	-0.0053
nr-er_FEST	0.775	2048	3	nr-er_FEST	0.7698	512	2	0.746337315	-0.0052
nr-er_FEST	0.775	2048	3	nr-er_FEST	0.7698	512	3	0.746337315	-0.0052
sr-mmp_FEST	0.8832	2048	2	sr-mmp_FEST	0.8781	512	1	0.635941806	-0.0051
sr-mmp_RFC	0.8859	2048	2	sr-mmp_RFC	0.8809	512	2	0.639333519	-0.005
sr-mmp_RFC	0.883	4096	1	sr-mmp_RFC	0.878	512	3	0.642751295	-0.005
nr-er_FEST	0.7697	4096	1	nr-er_FEST	0.7647	512	1	0.757321259	-0.005
cas-N6512_RFC	0.8836	2048	1	cas-N6512_RFC	0.8787	512	2	0.410334042	-0.0049
cas-N6512_SVM	0.765	512	2	cas-N6512_SVM	0.7601	512	3	0.551955997	-0.0049
nr-ahr_FEST	0.9022	256	1	nr-ahr_FEST	0.8973	512	3	0.661193313	-0.0049
nr-er_RFC	0.7699	2048	3	nr-er_RFC	0.765	512	1	0.761955881	-0.0049
nr-er_SVM	0.765	512	2	nr-er_SVM	0.7601	512	3	0.763318445	-0.0049
cas-N6512_FEST	0.8852	1024	2	cas-N6512_FEST	0.8804	512	2	0.416713567	-0.0048
cas-N6512_FEST	0.8852	2048	2	cas-N6512_FEST	0.8804	512	2	0.416713567	-0.0048
cas-N6512_SVM	0.7649	4096	3	cas-N6512_SVM	0.7601	512	3	0.560135692	-0.0048
nr-er_SVM	0.7649	4096	3	nr-er_SVM	0.7601	512	3	0.76802228	-0.0048
cas-N6512_SVM	0.7649	4096	3	cas-N6512_SVM	0.7602	512	1	0.568303205	-0.0047
cas-N6512_SVM	0.7648	256	2	cas-N6512_SVM	0.7601	512	3	0.5683722	-0.0047
nr-ahr_FEST	0.9104	4096	2	nr-ahr_FEST	0.9057	512	2	0.662627141	-0.0047
sr-mmp_FEST	0.8828	1024	2	sr-mmp_FEST	0.8781	512	1	0.662875565	-0.0047
nr-er_SVM	0.7649	4096	3	nr-er_SVM	0.7602	512	1	0.772707985	-0.0047
nr-er_SVM	0.7648	256	2	nr-er_SVM	0.7601	512	3	0.772734099	-0.0047
cas-N6512_RFC	0.8834	1024	2	cas-N6512_RFC	0.8787	512	2	0.42989541	-0.0047
sr-mmp_SVM	0.8806	1024	3	sr-mmp_SVM	0.876	512	2	0.671909918	-0.0046
cas-N6512_SVM	0.7648	256	2	cas-N6512_SVM	0.7602	512	1	0.576596709	-0.0046
sr-mmp_SVM	0.888	2048	2	sr-mmp_SVM	0.8834	512	1	0.663741767	-0.0046
sr-mmp_RFC	0.8826	1024	1	sr-mmp_RFC	0.878	512	3	0.669781312	-0.0046
nr-ahr_RFC	0.91	4096	2	nr-ahr_RFC	0.9054	512	1	0.669886941	-0.0046

nr-er_SVM	0.7648	256	2	nr-er_SVM	0.7602	512	1	0.777428145	-0.0046
nr-ahr_FEST	0.9104	4096	2	nr-ahr_FEST	0.9058	512	1	0.669302094	-0.0046
cas-N6512_RFC	0.8742	256	2	cas-N6512_RFC	0.8697	512	3	0.466211767	-0.0045
sr-mmp_FEST	0.8832	2048	2	sr-mmp_FEST	0.8787	512	2	0.675857552	-0.0045
nr-ahr_SVM	0.9012	512	1	nr-ahr_SVM	0.8967	512	2	0.688327297	-0.0045
cas-N6512_SVM	0.7694	1024	2	cas-N6512_SVM	0.765	512	2	0.590129298	-0.0044
sr-mmp_FEST	0.8825	2048	3	sr-mmp_FEST	0.8781	512	1	0.683342794	-0.0044
nr-er_SVM	0.7694	1024	2	nr-er_SVM	0.765	512	2	0.785684478	-0.0044
sr-mmp_SVM	0.8877	4096	1	sr-mmp_SVM	0.8834	512	1	0.684614736	-0.0043
cas-N6512_FEST	0.875	256	2	cas-N6512_FEST	0.8707	512	3	0.484738066	-0.0043
sr-mmp_RFC	0.8841	1024	2	sr-mmp_RFC	0.8799	512	1	0.695288232	-0.0042
sr-mmp_RFC	0.8841	1024	3	sr-mmp_RFC	0.8799	512	1	0.695288232	-0.0042
sr-mmp_RFC	0.8841	2048	3	sr-mmp_RFC	0.8799	512	1	0.695288232	-0.0042
nr-ahr_RFC	0.9096	2048	2	nr-ahr_RFC	0.9054	512	1	0.697375225	-0.0042
sr-mmp_FEST	0.8828	1024	2	sr-mmp_FEST	0.8787	512	2	0.703434616	-0.0041
sr-mmp_FEST	0.8807	2048	1	sr-mmp_FEST	0.8766	512	3	0.705474858	-0.0041
nr-ahr_RFC	0.91	4096	2	nr-ahr_RFC	0.906	512	2	0.71047531	-0.004
sr-mmp_RFC	0.882	2048	1	sr-mmp_RFC	0.878	512	3	0.711057424	-0.004
sr-mmp_FEST	0.8806	1024	3	sr-mmp_FEST	0.8766	512	3	0.712381891	-0.004
cas-N6512_RFC	0.8836	2048	1	cas-N6512_RFC	0.8798	512	1	0.522201629	-0.0038
nr-ahr_FEST	0.9095	2048	2	nr-ahr_FEST	0.9057	512	2	0.724842927	-0.0038
nr-ahr_SVM	0.905	1024	1	nr-ahr_SVM	0.9012	512	1	0.730206005	-0.0038
sr-mmp_FEST	0.8825	2048	3	sr-mmp_FEST	0.8787	512	2	0.724352841	-0.0038
nr-ahr_FEST	0.9095	2048	2	nr-ahr_FEST	0.9058	512	1	0.731740945	-0.0037
nr-ahr_RFC	0.9096	2048	2	nr-ahr_RFC	0.906	512	2	0.738545681	-0.0036
cas-N6512_RFC	0.8834	1024	2	cas-N6512_RFC	0.8798	512	1	0.544502598	-0.0036
cas-N6512_RFC	0.8823	4096	1	cas-N6512_RFC	0.8787	512	2	0.546351265	-0.0036
cas-N6512_RFC	0.8822	1024	1	cas-N6512_RFC	0.8787	512	2	0.557634983	-0.0035
sr-mmp_FEST	0.8801	4096	1	sr-mmp_FEST	0.8766	512	3	0.747225223	-0.0035
nr-ahr_SVM	0.9047	4096	3	nr-ahr_SVM	0.9012	512	1	0.750929259	-0.0035
nr-er_RFC	0.7735	4096	2	nr-er_RFC	0.77	512	3	0.827789935	-0.0035
cas-N6512_FEST	0.8838	256	1	cas-N6512_FEST	0.8804	512	2	0.566267376	-0.0034
cas-N6512_FEST	0.8741	128	1	cas-N6512_FEST	0.8707	512	3	0.581292914	-0.0034

sr-mmp_SVM	0.8793	256	1	sr-mmp_SVM	0.876	512	2	0.761766135	-0.0033	
sr-mmp_RFC	0.8841	1024	2	sr-mmp_RFC	0.8809	512	2	0.764974247	-0.0032	
sr-mmp_RFC	0.8841	1024	3	sr-mmp_RFC	0.8809	512	2	0.764974247	-0.0032	
sr-mmp_RFC	0.8841	2048	3	sr-mmp_RFC	0.8809	512	2	0.764974247	-0.0032	
nr-ahr_FEST	0.9088	2048	1	nr-ahr_FEST	0.9057	512	2	0.774356944	-0.0031	
sr-mmp_RFC	0.883	4096	1	sr-mmp_RFC	0.8799	512	1	0.772933163	-0.0031	
nr-ahr_FEST	0.9088	2048	1	nr-ahr_FEST	0.9058	512	1	0.781398386	-0.003	
nr-ahr_FEST	0.9086	1024	2	nr-ahr_FEST	0.9057	512	2	0.788653635	-0.0029	
sr-mmp_RFC	0.8809	512	2	sr-mmp_RFC	0.878	512	3	0.78865775	-0.0029	
nr-ahr_RFC	0.9083	2048	1	nr-ahr_RFC	0.9054	512	1	0.788941284	-0.0029	
nr-ahr_FEST	0.9086	1024	2	nr-ahr_FEST	0.9058	512	1	0.795730972	-0.0028	
sr-mmp_RFC	0.8826	1024	1	sr-mmp_RFC	0.8799	512	1	0.801712028	-0.0027	
sr-mmp_FEST	0.8807	2048	1	sr-mmp_FEST	0.8781	512	1	0.810111423	-0.0026	
nr-ahr_FEST	0.9083	4096	1	nr-ahr_FEST	0.9057	512	2	0.810205839	-0.0026	
nr-ahr_RFC	0.908	4096	1	nr-ahr_RFC	0.9054	512	1	0.810464887	-0.0026	
sr-mmp_FEST	0.8806	1024	3	sr-mmp_FEST	0.8781	512	1	0.81731422	-0.0025	
cas-N6512_RFC	0.8823	4096	1	cas-N6512_RFC	0.8798	512	1	0.674576134	-0.0025	
cas-N6512_SVM	0.7626	256	1	cas-N6512_SVM	0.7601	512	3	0.762014898	-0.0025	
nr-ahr_FEST	0.9083	4096	1	nr-ahr_FEST	0.9058	512	1	0.817332666	-0.0025	
nr-er_RFC	0.7725	2048	2	nr-er_RFC	0.77	512	3	0.876598111	-0.0025	
nr-er_FEST	0.7723	1024	3	nr-er_FEST	0.7698	512	2	0.876629303	-0.0025	
nr-er_FEST	0.7723	1024	3	nr-er_FEST	0.7698	512	3	0.876629303	-0.0025	
nr-er_SVM	0.7626	256	1	nr-er_SVM	0.7601	512	3	0.878070104	-0.0025	
cas-N6512_RFC	0.8822	1024	1	cas-N6512_RFC	0.8798	512	1	0.68695079	-0.0024	
cas-N6512_RFC	0.8811	2048	3	cas-N6512_RFC	0.8787	512	2	0.688304684	-0.0024	
cas-N6512_SVM	0.7626	256	1	cas-N6512_SVM	0.7602	512	1	0.771243953	-0.0024	
nr-er_RFC	0.7735	4096	2	nr-er_RFC	0.7711	512	2	0.881338709	-0.0024	
nr-er_SVM	0.7626	256	1	nr-er_SVM	0.7602	512	1	0.882904679	-0.0024	
nr-ahr_RFC	0.9083	2048	1	nr-ahr_RFC	0.906	512	2	0.831641043	-0.0023	
sr-mmp_SVM	0.8857	1024	1	sr-mmp_SVM	0.8834	512	1	0.828629381	-0.0023	
sr-mmp_SVM	0.8857	2048	3	sr-mmp_SVM	0.8834	512	1	0.828629381	-0.0023	
cas-N6512_SVM	0.7673	2048	3	cas-N6512_SVM	0.765	512	2	0.778692504	-0.0023	
nr-er_SVM	0.7673	2048	3	nr-er_SVM	0.765	512	2	0.887116094	-0.0023	

nr-er_FEST	0.767	1024	1	nr-er_FEST	0.7647	512	1	0.887157068	-0.0023
nr-ahr_RFC	0.9076	1024	2	nr-ahr_RFC	0.9054	512	1	0.839337147	-0.0022
nr-ahr_SVM	0.8989	256	1	nr-ahr_SVM	0.8967	512	2	0.845268398	-0.0022
nr-ahr_SVM	0.8885	128	1	nr-ahr_SVM	0.8863	512	3	0.851369721	-0.0022
nr-er_RFC	0.7722	1024	3	nr-er_RFC	0.77	512	3	0.891328974	-0.0022
cas-N6512_RFC	0.8808	4096	3	cas-N6512_RFC	0.8787	512	2	0.725742656	-0.0021
sr-mmp_RFC	0.883	4096	1	sr-mmp_RFC	0.8809	512	2	0.844762353	-0.0021
sr-mmp_RFC	0.882	2048	1	sr-mmp_RFC	0.8799	512	1	0.845299856	-0.0021
sr-mmp_FEST	0.8787	512	2	sr-mmp_FEST	0.8766	512	3	0.847022089	-0.0021
nr-er_FEST	0.7668	2048	1	nr-er_FEST	0.7647	512	1	0.896924696	-0.0021
cas-N6512_FEST	0.888	4096	1	cas-N6512_FEST	0.886	512	1	0.730391354	-0.002
sr-mmp_FEST	0.8807	2048	1	sr-mmp_FEST	0.8787	512	2	0.85321126	-0.002
nr-ahr_RFC	0.908	4096	1	nr-ahr_RFC	0.906	512	2	0.853434799	-0.002
sr-mmp_FEST	0.8801	4096	1	sr-mmp_FEST	0.8781	512	1	0.853510376	-0.002
nr-ahr_FEST	0.9077	4096	3	nr-ahr_FEST	0.9057	512	2	0.853636421	-0.002
nr-ahr_RFC	0.9074	1024	1	nr-ahr_RFC	0.9054	512	1	0.853837009	-0.002
nr-ahr_FEST	0.8993	256	2	nr-ahr_FEST	0.8973	512	3	0.858890177	-0.002
sr-mmp_FEST	0.8806	1024	3	sr-mmp_FEST	0.8787	512	2	0.860497355	-0.0019
sr-mmp_RFC	0.8799	512	1	sr-mmp_RFC	0.878	512	3	0.860828974	-0.0019
nr-ahr_SVM	0.9031	1024	2	nr-ahr_SVM	0.9012	512	1	0.863671707	-0.0019
nr-ahr_FEST	0.9077	4096	3	nr-ahr_FEST	0.9058	512	1	0.860846239	-0.0019
cas-N6512_FEST	0.8878	1024	1	cas-N6512_FEST	0.886	512	1	0.756555364	-0.0018
sr-mmp_RFC	0.8826	1024	1	sr-mmp_RFC	0.8809	512	2	0.874144002	-0.0017
nr-ahr_RFC	0.9014	256	1	nr-ahr_RFC	0.8997	512	3	0.878740584	-0.0017
cas-N6512_FEST	0.8876	2048	1	cas-N6512_FEST	0.886	512	1	0.782977956	-0.0016
nr-ahr_RFC	0.9076	1024	2	nr-ahr_RFC	0.906	512	2	0.882617119	-0.0016
cas-N6512_SVM	0.7616	1024	1	cas-N6512_SVM	0.7601	512	3	0.855943508	-0.0015
cas-N6512_SVM	0.7616	2048	1	cas-N6512_SVM	0.7601	512	3	0.855943508	-0.0015
nr-er_SVM	0.7616	1024	1	nr-er_SVM	0.7601	512	3	0.926701651	-0.0015
nr-er_SVM	0.7616	2048	1	nr-er_SVM	0.7601	512	3	0.926701651	-0.0015
sr-mmp_FEST	0.8781	512	1	sr-mmp_FEST	0.8766	512	3	0.890509438	-0.0015
nr-ahr_RFC	0.9012	1024	3	nr-ahr_RFC	0.8997	512	3	0.892960542	-0.0015
nr-ahr_RFC	0.9074	1024	1	nr-ahr_RFC	0.906	512	2	0.89725025	-0.0014

cas-N6512_SVM	0.7616	1024	1	cas-N6512_SVM	0.7602	512	1	0.86544074	-0.0014
cas-N6512_SVM	0.7616	2048	1	cas-N6512_SVM	0.7602	512	1	0.86544074	-0.0014
nr-er_SVM	0.7616	1024	1	nr-er_SVM	0.7602	512	1	0.931571844	-0.0014
nr-er_SVM	0.7616	2048	1	nr-er_SVM	0.7602	512	1	0.931571844	-0.0014
sr-mmp_FEST	0.8801	4096	1	sr-mmp_FEST	0.8787	512	2	0.897055764	-0.0014
nr-er_RFC	0.7725	2048	2	nr-er_RFC	0.7711	512	2	0.930656297	-0.0014
nr-er_RFC	0.7664	1024	1	nr-er_RFC	0.765	512	1	0.931180887	-0.0014
cas-N6512_RFC	0.8811	2048	3	cas-N6512_RFC	0.8798	512	1	0.827593398	-0.0013
nr-ahr_RFC	0.9067	4096	3	nr-ahr_RFC	0.9054	512	1	0.90483756	-0.0013
nr-ahr_FEST	0.9068	2048	3	nr-ahr_FEST	0.9057	512	2	0.919349742	-0.0011
cas-N6512_RFC	0.8798	512	1	cas-N6512_RFC	0.8787	512	2	0.854507408	-0.0011
sr-mmp_RFC	0.882	2048	1	sr-mmp_RFC	0.8809	512	2	0.918452218	-0.0011
nr-ahr_SVM	0.9023	2048	3	nr-ahr_SVM	0.9012	512	1	0.92095381	-0.0011
nr-er_RFC	0.7722	1024	3	nr-er_RFC	0.7711	512	2	0.945499906	-0.0011
nr-er_RFC	0.7711	512	2	nr-er_RFC	0.77	512	3	0.94557609	-0.0011
cas-N6512_RFC	0.8808	4096	3	cas-N6512_RFC	0.8798	512	1	0.867035282	-0.001
nr-ahr_FEST	0.9068	2048	3	nr-ahr_FEST	0.9058	512	1	0.926643074	-0.001
sr-mmp_FEST	0.8776	1024	1	sr-mmp_FEST	0.8766	512	3	0.926939863	-0.001
cas-N6512_SVM	0.7609	1024	3	cas-N6512_SVM	0.7601	512	3	0.922915876	-0.0008
nr-er_RFC	0.7658	256	3	nr-er_RFC	0.765	512	1	0.960656318	-0.0008
nr-er_SVM	0.7609	1024	3	nr-er_SVM	0.7601	512	3	0.960884148	-0.0008
nr-ahr_RFC	0.9005	256	2	nr-ahr_RFC	0.8997	512	3	0.942874871	-0.0008
nr-ahr_RFC	0.9067	4096	3	nr-ahr_RFC	0.906	512	2	0.948601458	-0.0007
cas-N6512_FEST	0.8811	4096	3	cas-N6512_FEST	0.8804	512	2	0.90652933	-0.0007
cas-N6512_SVM	0.7609	1024	3	cas-N6512_SVM	0.7602	512	1	0.932520858	-0.0007
nr-ahr_RFC	0.9061	2048	3	nr-ahr_RFC	0.9054	512	1	0.948742756	-0.0007
nr-er_SVM	0.7609	1024	3	nr-er_SVM	0.7602	512	1	0.965768445	-0.0007
nr-er_FEST	0.7654	256	3	nr-er_FEST	0.7647	512	1	0.965585592	-0.0007
cas-N6512_SVM	0.7607	4096	1	cas-N6512_SVM	0.7601	512	3	0.942157704	-0.0006
nr-er_SVM	0.7607	4096	1	nr-er_SVM	0.7601	512	3	0.970661397	-0.0006
cas-N6512_SVM	0.7607	4096	1	cas-N6512_SVM	0.7602	512	1	0.951780962	-0.0005
nr-er_SVM	0.7607	4096	1	nr-er_SVM	0.7602	512	1	0.975548075	-0.0005
cas-N6512_FEST	0.8864	4096	2	cas-N6512_FEST	0.886	512	1	0.945245849	-0.0004

sr-mmp_SVM	0.8838	2048	1	sr-mmp_SVM	0.8834	512	1	0.970073823	-0.0004
sr-mmp_RFC	0.8784	256	1	sr-mmp_RFC	0.878	512	3	0.970632822	-0.0004
nr-ahr_RFC	0.9061	2048	3	nr-ahr_RFC	0.906	512	2	0.99266254	-0.0001
nr-ahr_FEST	0.9058	512	1	nr-ahr_FEST	0.9057	512	2	0.992672627	-0.0001
cas-N6512_SVM	0.7602	512	1	cas-N6512_SVM	0.7601	512	3	0.99035567	-1E-04
nr-ahr_SVM	0.8864	128	2	nr-ahr_SVM	0.8863	512	3	0.993230845	-1E-04
nr-er_SVM	0.7602	512	1	nr-er_SVM	0.7601	512	3	0.995110589	-1E-04
nr-er_FEST	0.7698	512	2	nr-er_FEST	0.7698	512	3	1	0
cas-N6512_SVM	0.7649	4096	3	cas-N6512_SVM	0.765	512	2	0.990272723	1E-04
sr-mmp_SVM	0.8833	1024	2	sr-mmp_SVM	0.8834	512	1	0.992523536	1E-04
nr-er_RFC	0.7699	2048	3	nr-er_RFC	0.77	512	3	0.995052339	1E-04
nr-er_FEST	0.7697	4096	1	nr-er_FEST	0.7698	512	2	0.995053586	1E-04
nr-er_FEST	0.7697	4096	1	nr-er_FEST	0.7698	512	3	0.995053586	1E-04
nr-er_SVM	0.7649	4096	3	nr-er_SVM	0.765	512	2	0.99508277	1E-04
nr-er_FEST	0.7646	256	2	nr-er_FEST	0.7647	512	1	0.995084548	0.0001
cas-N6512_SVM	0.7648	256	2	cas-N6512_SVM	0.765	512	2	0.980548637	0.0002
nr-er_SVM	0.7648	256	2	nr-er_SVM	0.765	512	2	0.990166315	0.0002
nr-er_RFC	0.7646	256	2	nr-er_RFC	0.765	512	1	0.980336476	0.0004
cas-N6512_FEST	0.8799	2048	3	cas-N6512_FEST	0.8804	512	2	0.933326474	0.0005
sr-mmp_FEST	0.8776	1024	1	sr-mmp_FEST	0.8781	512	1	0.963339161	0.0005
nr-ahr_FEST	0.9052	1024	1	nr-ahr_FEST	0.9057	512	2	0.963425514	0.0005
nr-er_RFC	0.7645	4096	1	nr-er_RFC	0.765	512	1	0.975423464	0.0005
nr-ahr_RFC	0.9054	512	1	nr-ahr_RFC	0.906	512	2	0.956067268	0.0006
sr-mmp_FEST	0.8781	512	1	sr-mmp_FEST	0.8787	512	2	0.955931683	0.0006
nr-ahr_FEST	0.9052	1024	1	nr-ahr_FEST	0.9058	512	1	0.956107351	0.0006
cas-N6512_FEST	0.8852	1024	2	cas-N6512_FEST	0.886	512	1	0.891033564	0.0008
cas-N6512_FEST	0.8852	2048	2	cas-N6512_FEST	0.886	512	1	0.891033564	0.0008
nr-er_FEST	0.7638	256	1	nr-er_FEST	0.7647	512	1	0.955804367	0.0009
sr-mmp_RFC	0.8799	512	1	sr-mmp_RFC	0.8809	512	2	0.926114009	0.001
cas-N6512_RFC	0.8776	256	1	cas-N6512_RFC	0.8787	512	2	0.855153937	0.0011
sr-mmp_FEST	0.8776	1024	1	sr-mmp_FEST	0.8787	512	2	0.919373056	0.0011
sr-mmp_SVM	0.8696	256	2	sr-mmp_SVM	0.8707	512	3	0.921442036	0.0011
nr-er_RFC	0.7639	2048	1	nr-er_RFC	0.765	512	1	0.945983754	0.0011

nr-er_RFC	0.7699	2048	3	nr-er_RFC	0.7711	512	2	0.940640943	0.0012
sr-mmp_FEST	0.8753	256	1	sr-mmp_FEST	0.8766	512	3	0.905475166	0.0013
nr-ahr_RFC	0.8983	128	1	nr-ahr_RFC	0.8997	512	3	0.900668825	0.0014
sr-mmp_RFC	0.8784	256	1	sr-mmp_RFC	0.8799	512	1	0.889841949	0.0015
cas-N6512_RFC	0.8679	128	1	cas-N6512_RFC	0.8697	512	3	0.773356405	0.0018
cas-N6512_RFC	0.8766	1024	3	cas-N6512_RFC	0.8787	512	2	0.727997051	0.0021
cas-N6512_FEST	0.8838	256	1	cas-N6512_FEST	0.886	512	1	0.70721653	0.0022
cas-N6512_RFC	0.8776	256	1	cas-N6512_RFC	0.8798	512	1	0.71442853	0.0022
nr-ahr_SVM	0.8989	256	1	nr-ahr_SVM	0.9012	512	1	0.836812332	0.0023
nr-ahr_FEST	0.895	128	1	nr-ahr_FEST	0.8973	512	3	0.839409003	0.0023
nr-ahr_SVM	0.884	256	3	nr-ahr_SVM	0.8863	512	3	0.845950552	0.0023
cas-N6512_SVM	0.7626	256	1	cas-N6512_SVM	0.765	512	2	0.770289545	0.0024
nr-er_SVM	0.7626	256	1	nr-er_SVM	0.765	512	2	0.882574302	0.0024
sr-mmp_RFC	0.8784	256	1	sr-mmp_RFC	0.8809	512	2	0.817128806	0.0025
sr-mmp_SVM	0.8682	128	1	sr-mmp_SVM	0.8707	512	3	0.823034509	0.0025
sr-mmp_RFC	0.8754	256	2	sr-mmp_RFC	0.878	512	3	0.811809569	0.0026
sr-mmp_SVM	0.8806	1024	3	sr-mmp_SVM	0.8834	512	1	0.793998574	0.0028
nr-ahr_FEST	0.9029	1024	3	nr-ahr_FEST	0.9057	512	2	0.798366291	0.0028
sr-mmp_FEST	0.8753	256	1	sr-mmp_FEST	0.8781	512	1	0.797636223	0.0028
nr-er_FEST	0.767	1024	1	nr-er_FEST	0.7698	512	2	0.862420174	0.0028
nr-er_FEST	0.767	1024	1	nr-er_FEST	0.7698	512	3	0.862420174	0.0028
cas-N6512_FEST	0.8775	1024	3	cas-N6512_FEST	0.8804	512	2	0.629211123	0.0029
nr-ahr_FEST	0.9029	1024	3	nr-ahr_FEST	0.9058	512	1	0.791282606	0.0029
nr-er_FEST	0.7668	2048	1	nr-er_FEST	0.7698	512	2	0.852719602	0.003
nr-er_FEST	0.7668	2048	1	nr-er_FEST	0.7698	512	3	0.852719602	0.003
sr-mmp_FEST	0.8735	256	2	sr-mmp_FEST	0.8766	512	3	0.77768952	0.0031
cas-N6512_RFC	0.8766	1024	3	cas-N6512_RFC	0.8798	512	1	0.595311484	0.0032
nr-ahr_SVM	0.8935	1024	3	nr-ahr_SVM	0.8967	512	2	0.778899693	0.0032
cas-N6512_SVM	0.7616	1024	1	cas-N6512_SVM	0.765	512	2	0.679393013	0.0034
cas-N6512_SVM	0.7616	2048	1	cas-N6512_SVM	0.765	512	2	0.679393013	0.0034
nr-er_SVM	0.7616	1024	1	nr-er_SVM	0.765	512	2	0.834348484	0.0034
nr-er_SVM	0.7616	2048	1	nr-er_SVM	0.765	512	2	0.834348484	0.0034
sr-mmp_FEST	0.8753	256	1	sr-mmp_FEST	0.8787	512	2	0.755293778	0.0034

nr-ahr_FEST	0.9022	256	1	nr-ahr_FEST	0.9057	512	2	0.749855885	0.0035
cas-N6512_SVM	0.7565	128	1	cas-N6512_SVM	0.7601	512	3	0.664463639	0.0036
nr-ahr_FEST	0.9022	256	1	nr-ahr_FEST	0.9058	512	1	0.742902074	0.0036
nr-er_RFC	0.7664	1024	1	nr-er_RFC	0.77	512	3	0.823727927	0.0036
nr-er_SVM	0.7565	128	1	nr-er_SVM	0.7601	512	3	0.825758345	0.0036
cas-N6512_SVM	0.7565	128	1	cas-N6512_SVM	0.7602	512	1	0.65570812	0.0037
nr-er_RFC	0.7613	256	1	nr-er_RFC	0.765	512	1	0.82000373	0.0037
nr-er_SVM	0.7565	128	1	nr-er_SVM	0.7602	512	1	0.820989063	0.0037
nr-ahr_RFC	0.9014	256	1	nr-ahr_RFC	0.9054	512	1	0.716241702	0.004
cas-N6512_SVM	0.7609	1024	3	cas-N6512_SVM	0.765	512	2	0.618434597	0.0041
sr-mmp_SVM	0.8793	256	1	sr-mmp_SVM	0.8834	512	1	0.70284108	0.0041
nr-er_SVM	0.7609	1024	3	nr-er_SVM	0.765	512	2	0.800979165	0.0041
nr-ahr_RFC	0.9012	1024	3	nr-ahr_RFC	0.9054	512	1	0.702838065	0.0042
nr-er_RFC	0.7658	256	3	nr-er_RFC	0.77	512	3	0.795031286	0.0042
cas-N6512_SVM	0.7607	4096	1	cas-N6512_SVM	0.765	512	2	0.601480038	0.0043
nr-er_SVM	0.7607	4096	1	nr-er_SVM	0.765	512	2	0.791513485	0.0043
nr-ahr_SVM	0.8923	256	2	nr-ahr_SVM	0.8967	512	2	0.700153133	0.0044
nr-er_FEST	0.7654	256	3	nr-er_FEST	0.7698	512	2	0.785581306	0.0044
nr-er_FEST	0.7654	256	3	nr-er_FEST	0.7698	512	3	0.785581306	0.0044
cas-N6512_RFC	0.8742	256	2	cas-N6512_RFC	0.8787	512	2	0.458286351	0.0045
sr-mmp_RFC	0.8754	256	2	sr-mmp_RFC	0.8799	512	1	0.679307011	0.0045
nr-ahr_RFC	0.9014	256	1	nr-ahr_RFC	0.906	512	2	0.675528862	0.0046
sr-mmp_FEST	0.8735	256	2	sr-mmp_FEST	0.8781	512	1	0.67449355	0.0046
nr-er_RFC	0.7664	1024	1	nr-er_RFC	0.7711	512	2	0.771037486	0.0047
nr-ahr_RFC	0.9012	1024	3	nr-ahr_RFC	0.906	512	2	0.66241762	0.0048
cas-N6512_SVM	0.7602	512	1	cas-N6512_SVM	0.765	512	2	0.560065695	0.0048
nr-er_SVM	0.7602	512	1	nr-er_SVM	0.765	512	2	0.767995645	0.0048
cas-N6512_FEST	0.8811	4096	3	cas-N6512_FEST	0.886	512	1	0.405571508	0.0049
nr-ahr_RFC	0.9005	256	2	nr-ahr_RFC	0.9054	512	1	0.656760739	0.0049
nr-er_RFC	0.765	512	1	nr-er_RFC	0.77	512	3	0.757234312	0.005
sr-mmp_FEST	0.8715	128	1	sr-mmp_FEST	0.8766	512	3	0.643382996	0.0051
nr-er_FEST	0.7647	512	1	nr-er_FEST	0.7698	512	2	0.752609744	0.0051
nr-er_FEST	0.7647	512	1	nr-er_FEST	0.7698	512	3	0.752609744	0.0051

sr-mmp_FEST	0.8735	256	2	sr-mmp_FEST	0.8787	512	2	0.634610889	0.0052
sr-mmp_SVM	0.8655	256	3	sr-mmp_SVM	0.8707	512	3	0.643180429	0.0052
nr-er_FEST	0.7646	256	2	nr-er_FEST	0.7698	512	2	0.747936825	0.0052
nr-er_FEST	0.7646	256	2	nr-er_FEST	0.7698	512	3	0.747936825	0.0052
nr-er_RFC	0.7658	256	3	nr-er_RFC	0.7711	512	2	0.742874909	0.0053
cas-N6512_FEST	0.875	256	2	cas-N6512_FEST	0.8804	512	2	0.371049343	0.0054
nr-er_RFC	0.7596	128	1	nr-er_RFC	0.765	512	1	0.740072893	0.0054
nr-er_RFC	0.7646	256	2	nr-er_RFC	0.77	512	3	0.738558518	0.0054
nr-ahr_RFC	0.9005	256	2	nr-ahr_RFC	0.906	512	2	0.617445496	0.0055
cas-N6512_SVM	0.7546	128	2	cas-N6512_SVM	0.7601	512	3	0.50822534	0.0055
nr-er_SVM	0.7546	128	2	nr-er_SVM	0.7601	512	3	0.736888842	0.0055
sr-mmp_RFC	0.8754	256	2	sr-mmp_RFC	0.8809	512	2	0.612757601	0.0055
nr-er_RFC	0.7645	4096	1	nr-er_RFC	0.77	512	3	0.733914466	0.0055
cas-N6512_SVM	0.7546	128	2	cas-N6512_SVM	0.7602	512	1	0.500507596	0.0056
nr-er_SVM	0.7546	128	2	nr-er_SVM	0.7602	512	1	0.732272257	0.0056
cas-N6512_RFC	0.8742	256	2	cas-N6512_RFC	0.8798	512	1	0.354956743	0.0056
nr-ahr_FEST	0.8915	256	3	nr-ahr_FEST	0.8973	512	3	0.61183231	0.0058
nr-er_FEST	0.7589	128	2	nr-er_FEST	0.7647	512	1	0.721748218	0.0058
nr-er_FEST	0.7638	256	1	nr-er_FEST	0.7698	512	2	0.710918993	0.006
nr-er_FEST	0.7638	256	1	nr-er_FEST	0.7698	512	3	0.710918993	0.006
cas-N6512_FEST	0.8799	2048	3	cas-N6512_FEST	0.886	512	1	0.301718838	0.0061
nr-er_RFC	0.765	512	1	nr-er_RFC	0.7711	512	2	0.705890404	0.0061
nr-er_RFC	0.7639	2048	1	nr-er_RFC	0.77	512	3	0.706270956	0.0061
cas-N6512_FEST	0.8741	128	1	cas-N6512_FEST	0.8804	512	2	0.297551008	0.0063
sr-mmp_SVM	0.8696	256	2	sr-mmp_SVM	0.876	512	2	0.562822617	0.0064
nr-ahr_FEST	0.8993	256	2	nr-ahr_FEST	0.9057	512	2	0.562389941	0.0064
sr-mmp_FEST	0.8702	256	3	sr-mmp_FEST	0.8766	512	3	0.56206643	0.0064
nr-ahr_FEST	0.8993	256	2	nr-ahr_FEST	0.9058	512	1	0.556211767	0.0065
nr-er_RFC	0.7646	256	2	nr-er_RFC	0.7711	512	2	0.687662175	0.0065
sr-mmp_FEST	0.8715	128	1	sr-mmp_FEST	0.8781	512	1	0.548087643	0.0066
nr-ahr_FEST	0.8907	128	2	nr-ahr_FEST	0.8973	512	3	0.564247331	0.0066
nr-er_RFC	0.7645	4096	1	nr-er_RFC	0.7711	512	2	0.683134185	0.0066
sr-mmp_RFC	0.8709	128	1	sr-mmp_RFC	0.878	512	3	0.518670312	0.0071

nr-ahr_RFC	0.8983	128	1	nr-ahr_RFC	0.9054	512	1	0.521626652	0.0071	
sr-mmp_FEST	0.8715	128	1	sr-mmp_FEST	0.8787	512	2	0.511898275	0.0072	
nr-er_RFC	0.7639	2048	1	nr-er_RFC	0.7711	512	2	0.656221046	0.0072	
cas-N6512_SVM	0.7527	256	3	cas-N6512_SVM	0.7601	512	3	0.374156063	0.0074	
sr-mmp_RFC	0.8706	256	3	sr-mmp_RFC	0.878	512	3	0.501366415	0.0074	
nr-er_SVM	0.7527	256	3	nr-er_SVM	0.7601	512	3	0.651579895	0.0074	
nr-er_FEST	0.7573	128	3	nr-er_FEST	0.7647	512	1	0.649866193	0.0074	
cas-N6512_SVM	0.7527	256	3	cas-N6512_SVM	0.7602	512	1	0.367692962	0.0075	
nr-er_SVM	0.7527	256	3	nr-er_SVM	0.7602	512	1	0.647170154	0.0075	
nr-ahr_RFC	0.8922	256	3	nr-ahr_RFC	0.8997	512	3	0.509038271	0.0075	
nr-ahr_SVM	0.8787	128	3	nr-ahr_SVM	0.8863	512	3	0.524752324	0.0076	
nr-ahr_RFC	0.8983	128	1	nr-ahr_RFC	0.906	512	2	0.486479662	0.0077	
nr-ahr_SVM	0.8935	1024	3	nr-ahr_SVM	0.9012	512	1	0.495332922	0.0077	
sr-mmp_SVM	0.8682	128	1	sr-mmp_SVM	0.876	512	2	0.481635639	0.0078	
sr-mmp_FEST	0.8702	256	3	sr-mmp_FEST	0.8781	512	1	0.473110933	0.0079	
cas-N6512_RFC	0.8617	128	2	cas-N6512_RFC	0.8697	512	3	0.205743321	0.008	
nr-er_RFC	0.7569	128	2	nr-er_RFC	0.765	512	1	0.619284135	0.0081	
nr-ahr_RFC	0.8916	128	2	nr-ahr_RFC	0.8997	512	3	0.476274959	0.0081	
nr-ahr_SVM	0.8885	128	1	nr-ahr_SVM	0.8967	512	2	0.476218837	0.0082	
nr-er_FEST	0.7565	128	1	nr-er_FEST	0.7647	512	1	0.615108246	0.0082	
nr-er_RFC	0.7566	128	3	nr-er_RFC	0.765	512	1	0.60642729	0.0084	
cas-N6512_FEST	0.8775	1024	3	cas-N6512_FEST	0.886	512	1	0.152196832	0.0085	
cas-N6512_SVM	0.7565	128	1	cas-N6512_SVM	0.765	512	2	0.303672117	0.0085	
sr-mmp_FEST	0.8702	256	3	sr-mmp_FEST	0.8787	512	2	0.439710616	0.0085	
nr-er_SVM	0.7565	128	1	nr-er_SVM	0.765	512	2	0.602169284	0.0085	
nr-er_RFC	0.7613	256	1	nr-er_RFC	0.77	512	3	0.591496765	0.0087	
cas-N6512_FEST	0.8618	128	2	cas-N6512_FEST	0.8707	512	3	0.158373882	0.0089	
nr-ahr_SVM	0.8923	256	2	nr-ahr_SVM	0.9012	512	1	0.431752427	0.0089	
sr-mmp_RFC	0.8709	128	1	sr-mmp_RFC	0.8799	512	1	0.411816187	0.009	
sr-mmp_RFC	0.8706	256	3	sr-mmp_RFC	0.8799	512	1	0.396635295	0.0093	
cas-N6512_RFC	0.8602	256	3	cas-N6512_RFC	0.8697	512	3	0.133996674	0.0095	
nr-er_RFC	0.7613	256	1	nr-er_RFC	0.7711	512	2	0.545219165	0.0098	
cas-N6512_FEST	0.8607	256	3	cas-N6512_FEST	0.8707	512	3	0.113712877	0.01	

sr-mmp_RFC	0.8709	128	1	sr-mmp_RFC	0.8809	512	2	0.361018238	0.01
sr-mmp_RFC	0.8706	256	3	sr-mmp_RFC	0.8809	512	2	0.347027804	0.0103
nr-ahr_SVM	0.8864	128	2	nr-ahr_SVM	0.8967	512	2	0.372793531	0.0103
cas-N6512_SVM	0.7546	128	2	cas-N6512_SVM	0.765	512	2	0.208957377	0.0104
nr-er_RFC	0.7596	128	1	nr-er_RFC	0.77	512	3	0.521591217	0.0104
nr-er_SVM	0.7546	128	2	nr-er_SVM	0.765	512	2	0.524043212	0.0104
sr-mmp_SVM	0.8655	256	3	sr-mmp_SVM	0.876	512	2	0.345541517	0.0105
nr-ahr_FEST	0.895	128	1	nr-ahr_FEST	0.9057	512	2	0.337234262	0.0107
nr-ahr_FEST	0.895	128	1	nr-ahr_FEST	0.9058	512	1	0.332632787	0.0108
cas-N6512_RFC	0.8679	128	1	cas-N6512_RFC	0.8787	512	2	0.078741552	0.0108
sr-mmp_FEST	0.8657	128	2	sr-mmp_FEST	0.8766	512	3	0.326860965	0.0109
nr-er_FEST	0.7589	128	2	nr-er_FEST	0.7698	512	2	0.501997682	0.0109
nr-er_FEST	0.7589	128	2	nr-er_FEST	0.7698	512	3	0.501997682	0.0109
cas-N6512_FEST	0.875	256	2	cas-N6512_FEST	0.886	512	1	0.065284741	0.011
nr-er_RFC	0.7596	128	1	nr-er_RFC	0.7711	512	2	0.478219622	0.0115
cas-N6512_RFC	0.8679	128	1	cas-N6512_RFC	0.8798	512	1	0.052223565	0.0119
cas-N6512_SVM	0.7527	256	3	cas-N6512_SVM	0.765	512	2	0.137927988	0.0123
nr-er_SVM	0.7527	256	3	nr-er_SVM	0.765	512	2	0.451611278	0.0123
sr-mmp_FEST	0.8657	128	2	sr-mmp_FEST	0.8781	512	1	0.263525415	0.0124
nr-er_FEST	0.7573	128	3	nr-er_FEST	0.7698	512	2	0.441782243	0.0125
nr-er_FEST	0.7573	128	3	nr-er_FEST	0.7698	512	3	0.441782243	0.0125
nr-ahr_SVM	0.8885	128	1	nr-ahr_SVM	0.9012	512	1	0.265529219	0.0127
nr-ahr_SVM	0.884	256	3	nr-ahr_SVM	0.8967	512	2	0.273966503	0.0127
sr-mmp_FEST	0.8657	128	2	sr-mmp_FEST	0.8787	512	2	0.240656336	0.013
nr-er_RFC	0.7569	128	2	nr-er_RFC	0.77	512	3	0.420229137	0.0131
nr-ahr_RFC	0.8922	256	3	nr-ahr_RFC	0.9054	512	1	0.239505073	0.0132
nr-er_FEST	0.7565	128	1	nr-er_FEST	0.7698	512	2	0.413333515	0.0133
nr-er_FEST	0.7565	128	1	nr-er_FEST	0.7698	512	3	0.413333515	0.0133
nr-er_RFC	0.7566	128	3	nr-er_RFC	0.77	512	3	0.409745652	0.0134
sr-mmp_RFC	0.8645	128	2	sr-mmp_RFC	0.878	512	3	0.224434135	0.0135
nr-ahr_RFC	0.8922	256	3	nr-ahr_RFC	0.906	512	2	0.218217346	0.0138
sr-mmp_SVM	0.8696	256	2	sr-mmp_SVM	0.8834	512	1	0.206510914	0.0138
nr-ahr_RFC	0.8916	128	2	nr-ahr_RFC	0.9054	512	1	0.219380278	0.0138

nr-ahr_FEST	0.8915	256	3	nr-ahr_FEST	0.9057	512	2	0.206110426	0.0142
nr-er_RFC	0.7569	128	2	nr-er_RFC	0.7711	512	2	0.381952405	0.0142
nr-ahr_FEST	0.8915	256	3	nr-ahr_FEST	0.9058	512	1	0.202836755	0.0143
nr-ahr_RFC	0.8916	128	2	nr-ahr_RFC	0.906	512	2	0.199410656	0.0144
nr-er_RFC	0.7566	128	3	nr-er_RFC	0.7711	512	2	0.372059148	0.0145
nr-ahr_SVM	0.8864	128	2	nr-ahr_SVM	0.9012	512	1	0.196277619	0.0148
nr-ahr_FEST	0.8907	128	2	nr-ahr_FEST	0.9057	512	2	0.182405528	0.015
nr-ahr_FEST	0.8907	128	2	nr-ahr_FEST	0.9058	512	1	0.179413832	0.0151
sr-mmp_SVM	0.8682	128	1	sr-mmp_SVM	0.8834	512	1	0.165071253	0.0152
sr-mmp_RFC	0.8645	128	2	sr-mmp_RFC	0.8799	512	1	0.164497831	0.0154
sr-mmp_SVM	0.855	128	2	sr-mmp_SVM	0.8707	512	3	0.168423405	0.0157
sr-mmp_RFC	0.8645	128	2	sr-mmp_RFC	0.8809	512	2	0.138124569	0.0164
nr-ahr_SVM	0.884	256	3	nr-ahr_SVM	0.9012	512	1	0.13494231	0.0172
nr-ahr_RFC	0.8825	128	3	nr-ahr_RFC	0.8997	512	3	0.137223132	0.0172
sr-mmp_SVM	0.8655	256	3	sr-mmp_SVM	0.8834	512	1	0.103574617	0.0179
nr-ahr_SVM	0.8787	128	3	nr-ahr_SVM	0.8967	512	2	0.124646084	0.018
nr-ahr_FEST	0.8791	128	3	nr-ahr_FEST	0.8973	512	3	0.119819843	0.0182
nr-er_SVM	0.7407	128	3	nr-er_SVM	0.7601	512	3	0.239405511	0.0194
nr-er_SVM	0.7407	128	3	nr-er_SVM	0.7602	512	1	0.236965595	0.0195
sr-mmp_SVM	0.8506	128	3	sr-mmp_SVM	0.8707	512	3	0.07969175	0.0201
sr-mmp_FEST	0.8558	128	3	sr-mmp_FEST	0.8766	512	3	0.065273162	0.0208
sr-mmp_SVM	0.855	128	2	sr-mmp_SVM	0.876	512	2	0.063280318	0.021
nr-ahr_SVM	0.8787	128	3	nr-ahr_SVM	0.9012	512	1	0.052827134	0.0225
nr-er_SVM	0.7407	128	3	nr-er_SVM	0.765	512	2	0.13946178	0.0243
nr-ahr_SVM	0.905	1024	1	nr-ahr_SVM	0.884	256	3	0.065854735	-0.021
sr-mmp_SVM	0.8857	1024	1	sr-mmp_SVM	0.8655	256	3	0.065165688	-0.0202
sr-mmp_SVM	0.8857	2048	3	sr-mmp_SVM	0.8655	256	3	0.065165688	-0.0202
nr-er_SVM	0.7725	2048	2	nr-er_SVM	0.7527	256	3	0.223492853	-0.0198
nr-ahr_SVM	0.9031	1024	2	nr-ahr_SVM	0.884	256	3	0.095624063	-0.0191
sr-mmp_SVM	0.888	2048	2	sr-mmp_SVM	0.8696	256	2	0.089544362	-0.0184
nr-ahr_SVM	0.9023	2048	3	nr-ahr_SVM	0.884	256	3	0.110925821	-0.0183
sr-mmp_SVM	0.8838	2048	1	sr-mmp_SVM	0.8655	256	3	0.095859814	-0.0183
nr-ahr_FEST	0.9095	2048	2	nr-ahr_FEST	0.8915	256	3	0.106089186	-0.018

sr-mmp_SVM	0.8833	1024	2	sr-mmp_SVM	0.8655	256	3	0.105576682	-0.0178
nr-ahr_RFC	0.9096	2048	2	nr-ahr_RFC	0.8922	256	3	0.117632664	-0.0174
nr-ahr_FEST	0.9088	2048	1	nr-ahr_FEST	0.8915	256	3	0.120954691	-0.0173
nr-ahr_FEST	0.9086	1024	2	nr-ahr_FEST	0.8915	256	3	0.125478973	-0.0171
nr-er_SVM	0.7694	1024	2	nr-er_SVM	0.7527	256	3	0.305492024	-0.0167
sr-mmp_SVM	0.8857	1024	1	sr-mmp_SVM	0.8696	256	2	0.138977581	-0.0161
sr-mmp_SVM	0.8857	2048	3	sr-mmp_SVM	0.8696	256	2	0.138977581	-0.0161
nr-ahr_RFC	0.9083	2048	1	nr-ahr_RFC	0.8922	256	3	0.148817416	-0.0161
nr-er_RFC	0.777	1024	2	nr-er_RFC	0.7613	256	1	0.330642115	-0.0157
nr-ahr_SVM	0.908	2048	2	nr-ahr_SVM	0.8923	256	2	0.159363103	-0.0157
nr-ahr_SVM	0.9077	2048	1	nr-ahr_SVM	0.8923	256	2	0.167765581	-0.0154
nr-ahr_RFC	0.9076	1024	2	nr-ahr_RFC	0.8922	256	3	0.167946967	-0.0154
nr-ahr_FEST	0.9068	2048	3	nr-ahr_FEST	0.8915	256	3	0.172091154	-0.0153
sr-mmp_RFC	0.8859	2048	2	sr-mmp_RFC	0.8706	256	3	0.158865188	-0.0153
nr-ahr_RFC	0.9074	1024	1	nr-ahr_RFC	0.8922	256	3	0.173725968	-0.0152
nr-er_FEST	0.779	1024	2	nr-er_FEST	0.7638	256	1	0.344908808	-0.0152
nr-er_FEST	0.779	2048	2	nr-er_FEST	0.7638	256	1	0.344908808	-0.0152
sr-mmp_SVM	0.8806	1024	3	sr-mmp_SVM	0.8655	256	3	0.171709882	-0.0151
nr-ahr_SVM	0.8989	256	1	nr-ahr_SVM	0.884	256	3	0.197380982	-0.0149
nr-er_SVM	0.7673	2048	3	nr-er_SVM	0.7527	256	3	0.370936208	-0.0146
cas-N6512_SVM	0.7673	2048	3	cas-N6512_SVM	0.7527	256	3	0.077635388	-0.0146
nr-er_FEST	0.779	1024	2	nr-er_FEST	0.7646	256	2	0.370660487	-0.0144
nr-er_FEST	0.779	2048	2	nr-er_FEST	0.7646	256	2	0.370660487	-0.0144
sr-mmp_SVM	0.8838	2048	1	sr-mmp_SVM	0.8696	256	2	0.193353055	-0.0142
nr-ahr_RFC	0.9061	2048	3	nr-ahr_RFC	0.8922	256	3	0.214800569	-0.0139
sr-mmp_SVM	0.8793	256	1	sr-mmp_SVM	0.8655	256	3	0.212621245	-0.0138
nr-ahr_FEST	0.9052	1024	1	nr-ahr_FEST	0.8915	256	3	0.22303181	-0.0137
sr-mmp_SVM	0.8833	1024	2	sr-mmp_SVM	0.8696	256	2	0.209896662	-0.0137
nr-er_FEST	0.779	1024	2	nr-er_FEST	0.7654	256	3	0.397598876	-0.0136
nr-er_FEST	0.779	2048	2	nr-er_FEST	0.7654	256	3	0.397598876	-0.0136
sr-mmp_RFC	0.8841	1024	2	sr-mmp_RFC	0.8706	256	3	0.215245742	-0.0135
sr-mmp_RFC	0.8841	1024	3	sr-mmp_RFC	0.8706	256	3	0.215245742	-0.0135
sr-mmp_RFC	0.8841	2048	3	sr-mmp_RFC	0.8706	256	3	0.215245742	-0.0135

sr-mmp_FEST	0.8832	2048	2	sr-mmp_FEST	0.8702	256	3	0.233753396	-0.013
nr-ahr_SVM	0.905	1024	1	nr-ahr_SVM	0.8923	256	2	0.258084334	-0.0127
sr-mmp_FEST	0.8828	1024	2	sr-mmp_FEST	0.8702	256	3	0.248779609	-0.0126
nr-er_RFC	0.777	1024	2	nr-er_RFC	0.7646	256	2	0.441374212	-0.0124
sr-mmp_FEST	0.8825	2048	3	sr-mmp_FEST	0.8702	256	3	0.260469165	-0.0123
cas-N6512_SVM	0.7648	256	2	cas-N6512_SVM	0.7527	256	3	0.144518326	-0.0121
nr-er_SVM	0.7648	256	2	nr-er_SVM	0.7527	256	3	0.459054965	-0.0121
sr-mmp_RFC	0.8826	1024	1	sr-mmp_RFC	0.8706	256	3	0.271880761	-0.012
nr-ahr_FEST	0.9029	1024	3	nr-ahr_FEST	0.8915	256	3	0.312975622	-0.0114
sr-mmp_RFC	0.882	2048	1	sr-mmp_RFC	0.8706	256	3	0.297069476	-0.0114
nr-er_RFC	0.777	1024	2	nr-er_RFC	0.7658	256	3	0.486501893	-0.0112
nr-er_FEST	0.775	2048	3	nr-er_FEST	0.7638	256	1	0.487604969	-0.0112
nr-er_RFC	0.7725	2048	2	nr-er_RFC	0.7613	256	1	0.488955108	-0.0112
sr-mmp_SVM	0.8806	1024	3	sr-mmp_SVM	0.8696	256	2	0.316272477	-0.011
cas-N6512_RFC	0.8852	2048	2	cas-N6512_RFC	0.8742	256	2	0.066180398	-0.011
nr-er_RFC	0.7722	1024	3	nr-er_RFC	0.7613	256	1	0.500751053	-0.0109
nr-ahr_SVM	0.9031	1024	2	nr-ahr_SVM	0.8923	256	2	0.338138286	-0.0108
nr-ahr_FEST	0.9022	256	1	nr-ahr_FEST	0.8915	256	3	0.344326623	-0.0107
sr-mmp_RFC	0.8859	2048	2	sr-mmp_RFC	0.8754	256	2	0.329694246	-0.0105
sr-mmp_FEST	0.8807	2048	1	sr-mmp_FEST	0.8702	256	3	0.338221841	-0.0105
sr-mmp_FEST	0.8806	1024	3	sr-mmp_FEST	0.8702	256	3	0.342923704	-0.0104
nr-er_FEST	0.775	2048	3	nr-er_FEST	0.7646	256	2	0.519020181	-0.0104
nr-ahr_FEST	0.9095	2048	2	nr-ahr_FEST	0.8993	256	2	0.351749363	-0.0102
cas-N6512_FEST	0.8852	1024	2	cas-N6512_FEST	0.875	256	2	0.087934247	-0.0102
cas-N6512_FEST	0.8852	2048	2	cas-N6512_FEST	0.875	256	2	0.087934247	-0.0102
nr-ahr_SVM	0.9023	2048	3	nr-ahr_SVM	0.8923	256	2	0.375939131	-0.01
cas-N6512_SVM	0.7725	2048	2	cas-N6512_SVM	0.7626	256	1	0.225207462	-0.0099
nr-er_SVM	0.7725	2048	2	nr-er_SVM	0.7626	256	1	0.540453459	-0.0099
cas-N6512_SVM	0.7626	256	1	cas-N6512_SVM	0.7527	256	3	0.233436412	-0.0099
nr-er_SVM	0.7626	256	1	nr-er_SVM	0.7527	256	3	0.54517892	-0.0099
sr-mmp_FEST	0.8832	2048	2	sr-mmp_FEST	0.8735	256	2	0.371675217	-0.0097
sr-mmp_SVM	0.8793	256	1	sr-mmp_SVM	0.8696	256	2	0.377887955	-0.0097
nr-er_FEST	0.775	2048	3	nr-er_FEST	0.7654	256	3	0.551480213	-0.0096

nr-ahr_FEST	0.9088	2048	1	nr-ahr_FEST	0.8993	256	2	0.386539606	-0.0095
nr-ahr_SVM	0.8935	1024	3	nr-ahr_SVM	0.884	256	3	0.416050853	-0.0095
cas-N6512_RFC	0.8836	2048	1	cas-N6512_RFC	0.8742	256	2	0.117632105	-0.0094
sr-mmp_FEST	0.8828	1024	2	sr-mmp_FEST	0.8735	256	2	0.392047936	-0.0093
nr-ahr_FEST	0.9086	1024	2	nr-ahr_FEST	0.8993	256	2	0.396831055	-0.0093
cas-N6512_RFC	0.8834	1024	2	cas-N6512_RFC	0.8742	256	2	0.125802294	-0.0092
nr-ahr_RFC	0.9014	256	1	nr-ahr_RFC	0.8922	256	3	0.416291313	-0.0092
nr-ahr_RFC	0.9096	2048	2	nr-ahr_RFC	0.9005	256	2	0.404738564	-0.0091
nr-ahr_SVM	0.908	2048	2	nr-ahr_SVM	0.8989	256	1	0.408112899	-0.0091
nr-ahr_RFC	0.9012	1024	3	nr-ahr_RFC	0.8922	256	3	0.426689412	-0.009
sr-mmp_FEST	0.8825	2048	3	sr-mmp_FEST	0.8735	256	2	0.407743467	-0.009
cas-N6512_SVM	0.7616	1024	1	cas-N6512_SVM	0.7527	256	3	0.284503619	-0.0089
cas-N6512_SVM	0.7616	2048	1	cas-N6512_SVM	0.7527	256	3	0.284503619	-0.0089
nr-er_SVM	0.7616	1024	1	nr-er_SVM	0.7527	256	3	0.58673569	-0.0089
nr-er_SVM	0.7616	2048	1	nr-er_SVM	0.7527	256	3	0.58673569	-0.0089
cas-N6512_FEST	0.8838	256	1	cas-N6512_FEST	0.875	256	2	0.142126644	-0.0088
nr-ahr_SVM	0.9077	2048	1	nr-ahr_SVM	0.8989	256	1	0.424054608	-0.0088
sr-mmp_SVM	0.888	2048	2	sr-mmp_SVM	0.8793	256	1	0.414378392	-0.0087
sr-mmp_RFC	0.8841	1024	2	sr-mmp_RFC	0.8754	256	2	0.420776854	-0.0087
sr-mmp_RFC	0.8841	1024	3	sr-mmp_RFC	0.8754	256	2	0.420776854	-0.0087
sr-mmp_RFC	0.8841	2048	3	sr-mmp_RFC	0.8754	256	2	0.420776854	-0.0087
nr-er_RFC	0.7699	2048	3	nr-er_RFC	0.7613	256	1	0.595788455	-0.0086
nr-er_FEST	0.7723	1024	3	nr-er_FEST	0.7638	256	1	0.598974053	-0.0085
nr-ahr_RFC	0.9005	256	2	nr-ahr_RFC	0.8922	256	3	0.464180335	-0.0083
nr-ahr_SVM	0.8923	256	2	nr-ahr_SVM	0.884	256	3	0.478359178	-0.0083
cas-N6512_SVM	0.7609	1024	3	cas-N6512_SVM	0.7527	256	3	0.32438948	-0.0082
nr-ahr_RFC	0.9096	2048	2	nr-ahr_RFC	0.9014	256	1	0.451869696	-0.0082
nr-er_SVM	0.7609	1024	3	nr-er_SVM	0.7527	256	3	0.61664076	-0.0082
cas-N6512_RFC	0.8822	1024	1	cas-N6512_RFC	0.8742	256	2	0.184205412	-0.008
sr-mmp_FEST	0.8832	2048	2	sr-mmp_FEST	0.8753	256	1	0.465529214	-0.0079
nr-er_RFC	0.7725	2048	2	nr-er_RFC	0.7646	256	2	0.624802693	-0.0079
nr-ahr_RFC	0.9083	2048	1	nr-ahr_RFC	0.9005	256	2	0.476442392	-0.0078
nr-ahr_FEST	0.8993	256	2	nr-ahr_FEST	0.8915	256	3	0.493201654	-0.0078

sr-mmp_RFC	0.8784	256	1	sr-mmp_RFC	0.8706	256	3	0.478229154	-0.0078
cas-N6512_RFC	0.8679	128	1	cas-N6512_RFC	0.8602	256	3	0.226039531	-0.0077
nr-er_FEST	0.7723	1024	3	nr-er_FEST	0.7646	256	2	0.633637596	-0.0077
cas-N6512_SVM	0.7725	2048	2	cas-N6512_SVM	0.7648	256	2	0.344565639	-0.0077
nr-er_SVM	0.7725	2048	2	nr-er_SVM	0.7648	256	2	0.633553005	-0.0077
nr-er_RFC	0.7722	1024	3	nr-er_RFC	0.7646	256	2	0.638073948	-0.0076
cas-N6512_RFC	0.8852	2048	2	cas-N6512_RFC	0.8776	256	1	0.201136009	-0.0076
sr-mmp_RFC	0.8859	2048	2	sr-mmp_RFC	0.8784	256	1	0.484017497	-0.0075
sr-mmp_FEST	0.8828	1024	2	sr-mmp_FEST	0.8753	256	1	0.488723467	-0.0075
nr-ahr_FEST	0.9068	2048	3	nr-ahr_FEST	0.8993	256	2	0.496168641	-0.0075
sr-mmp_FEST	0.8776	1024	1	sr-mmp_FEST	0.8702	256	3	0.501923864	-0.0074
nr-ahr_FEST	0.9095	2048	2	nr-ahr_FEST	0.9022	256	1	0.502356307	-0.0073
sr-mmp_RFC	0.8826	1024	1	sr-mmp_RFC	0.8754	256	2	0.506351921	-0.0072
sr-mmp_FEST	0.8825	2048	3	sr-mmp_FEST	0.8753	256	1	0.506497448	-0.0072
sr-mmp_FEST	0.8807	2048	1	sr-mmp_FEST	0.8735	256	2	0.509086769	-0.0072
sr-mmp_FEST	0.8806	1024	3	sr-mmp_FEST	0.8735	256	2	0.515059775	-0.0071
nr-ahr_RFC	0.9076	1024	2	nr-ahr_RFC	0.9005	256	2	0.517564311	-0.0071
cas-N6512_RFC	0.8811	2048	3	cas-N6512_RFC	0.8742	256	2	0.253137331	-0.0069
nr-ahr_RFC	0.9083	2048	1	nr-ahr_RFC	0.9014	256	1	0.527946998	-0.0069
nr-ahr_RFC	0.9074	1024	1	nr-ahr_RFC	0.9005	256	2	0.529616682	-0.0069
nr-er_FEST	0.7723	1024	3	nr-er_FEST	0.7654	256	3	0.669158461	-0.0069
cas-N6512_SVM	0.7694	1024	2	cas-N6512_SVM	0.7626	256	1	0.406174979	-0.0068
nr-er_SVM	0.7694	1024	2	nr-er_SVM	0.7626	256	1	0.67475441	-0.0068
nr-er_RFC	0.7725	2048	2	nr-er_RFC	0.7658	256	3	0.678088982	-0.0067
sr-mmp_RFC	0.882	2048	1	sr-mmp_RFC	0.8754	256	2	0.542843028	-0.0066
nr-ahr_FEST	0.9088	2048	1	nr-ahr_FEST	0.9022	256	1	0.544857771	-0.0066
nr-ahr_SVM	0.8989	256	1	nr-ahr_SVM	0.8923	256	2	0.561740744	-0.0066
sr-mmp_SVM	0.8857	1024	1	sr-mmp_SVM	0.8793	256	1	0.549881316	-0.0064
sr-mmp_SVM	0.8857	2048	3	sr-mmp_SVM	0.8793	256	1	0.549881316	-0.0064
nr-ahr_FEST	0.9086	1024	2	nr-ahr_FEST	0.9022	256	1	0.557294434	-0.0064
nr-er_RFC	0.7722	1024	3	nr-er_RFC	0.7658	256	3	0.691801818	-0.0064
nr-ahr_RFC	0.9076	1024	2	nr-ahr_RFC	0.9014	256	1	0.571252129	-0.0062
nr-ahr_SVM	0.905	1024	1	nr-ahr_SVM	0.8989	256	1	0.581765447	-0.0061

nr-ahr_RFC	0.8983	128	1	nr-ahr_RFC	0.8922	256	3	0.592280054	-0.0061
cas-N6512_RFC	0.8836	2048	1	cas-N6512_RFC	0.8776	256	1	0.314501044	-0.006
nr-ahr_RFC	0.9074	1024	1	nr-ahr_RFC	0.9014	256	1	0.583901797	-0.006
nr-ahr_FEST	0.9052	1024	1	nr-ahr_FEST	0.8993	256	2	0.593724921	-0.0059
cas-N6512_RFC	0.8834	1024	2	cas-N6512_RFC	0.8776	256	1	0.331105678	-0.0058
sr-mmp_RFC	0.8841	1024	2	sr-mmp_RFC	0.8784	256	1	0.595981692	-0.0057
sr-mmp_RFC	0.8841	1024	3	sr-mmp_RFC	0.8784	256	1	0.595981692	-0.0057
sr-mmp_RFC	0.8841	2048	3	sr-mmp_RFC	0.8784	256	1	0.595981692	-0.0057
nr-ahr_RFC	0.9061	2048	3	nr-ahr_RFC	0.9005	256	2	0.610984055	-0.0056
sr-mmp_FEST	0.8807	2048	1	sr-mmp_FEST	0.8753	256	1	0.619411229	-0.0054
sr-mmp_FEST	0.8806	1024	3	sr-mmp_FEST	0.8753	256	1	0.625972237	-0.0053
nr-er_RFC	0.7699	2048	3	nr-er_RFC	0.7646	256	2	0.743243208	-0.0053
sr-mmp_FEST	0.8753	256	1	sr-mmp_FEST	0.8702	256	3	0.644776218	-0.0051
nr-er_RFC	0.7664	1024	1	nr-er_RFC	0.7613	256	1	0.753595343	-0.0051
cas-N6512_FEST	0.8799	2048	3	cas-N6512_FEST	0.875	256	2	0.41744565	-0.0049
sr-mmp_RFC	0.8754	256	2	sr-mmp_RFC	0.8706	256	3	0.664100295	-0.0048
cas-N6512_SVM	0.7673	2048	3	cas-N6512_SVM	0.7626	256	1	0.566631791	-0.0047
nr-ahr_RFC	0.9061	2048	3	nr-ahr_RFC	0.9014	256	1	0.668820236	-0.0047
nr-er_SVM	0.7673	2048	3	nr-er_SVM	0.7626	256	1	0.772073439	-0.0047
nr-ahr_FEST	0.9068	2048	3	nr-ahr_FEST	0.9022	256	1	0.674429626	-0.0046
cas-N6512_RFC	0.8822	1024	1	cas-N6512_RFC	0.8776	256	1	0.441960002	-0.0046
cas-N6512_SVM	0.7694	1024	2	cas-N6512_SVM	0.7648	256	2	0.573414566	-0.0046
nr-er_SVM	0.7694	1024	2	nr-er_SVM	0.7648	256	2	0.776223206	-0.0046
sr-mmp_SVM	0.8838	2048	1	sr-mmp_SVM	0.8793	256	1	0.675213993	-0.0045
nr-ahr_SVM	0.8885	128	1	nr-ahr_SVM	0.884	256	3	0.702716428	-0.0045
sr-mmp_RFC	0.8826	1024	1	sr-mmp_RFC	0.8784	256	1	0.696806936	-0.0042
nr-ahr_SVM	0.9031	1024	2	nr-ahr_SVM	0.8989	256	1	0.70567586	-0.0042
sr-mmp_FEST	0.8776	1024	1	sr-mmp_FEST	0.8735	256	2	0.708384986	-0.0041
sr-mmp_SVM	0.8696	256	2	sr-mmp_SVM	0.8655	256	3	0.715379765	-0.0041
nr-er_RFC	0.7699	2048	3	nr-er_RFC	0.7658	256	3	0.799818801	-0.0041
cas-N6512_FEST	0.8878	1024	1	cas-N6512_FEST	0.8838	256	1	0.492974157	-0.004
sr-mmp_SVM	0.8833	1024	2	sr-mmp_SVM	0.8793	256	1	0.709805143	-0.004
cas-N6512_FEST	0.8876	2048	1	cas-N6512_FEST	0.8838	256	1	0.515032299	-0.0038

cas-N6512_SVM	0.7565	128	1	cas-N6512_SVM	0.7527	256	3	0.649140503	-0.0038
nr-er_SVM	0.7565	128	1	nr-er_SVM	0.7527	256	3	0.816992657	-0.0038
sr-mmp_RFC	0.882	2048	1	sr-mmp_RFC	0.8784	256	1	0.738664672	-0.0036
nr-ahr_FEST	0.9029	1024	3	nr-ahr_FEST	0.8993	256	2	0.746048527	-0.0036
nr-ahr_FEST	0.895	128	1	nr-ahr_FEST	0.8915	256	3	0.760503033	-0.0035
cas-N6512_RFC	0.8811	2048	3	cas-N6512_RFC	0.8776	256	1	0.559425843	-0.0035
cas-N6512_RFC	0.8776	256	1	cas-N6512_RFC	0.8742	256	2	0.576066159	-0.0034
nr-ahr_SVM	0.9023	2048	3	nr-ahr_SVM	0.8989	256	1	0.760211533	-0.0034
sr-mmp_FEST	0.8735	256	2	sr-mmp_FEST	0.8702	256	3	0.766122832	-0.0033
nr-er_FEST	0.767	1024	1	nr-er_FEST	0.7638	256	1	0.843574913	-0.0032
sr-mmp_RFC	0.8784	256	1	sr-mmp_RFC	0.8754	256	2	0.783387835	-0.003
nr-ahr_FEST	0.9052	1024	1	nr-ahr_FEST	0.9022	256	1	0.7848721	-0.003
nr-er_FEST	0.7668	2048	1	nr-er_FEST	0.7638	256	1	0.853254542	-0.003
nr-ahr_FEST	0.9022	256	1	nr-ahr_FEST	0.8993	256	2	0.794488459	-0.0029
sr-mmp_SVM	0.8682	128	1	sr-mmp_SVM	0.8655	256	3	0.810628765	-0.0027
nr-er_RFC	0.7639	2048	1	nr-er_RFC	0.7613	256	1	0.873048376	-0.0026
cas-N6512_FEST	0.8775	1024	3	cas-N6512_FEST	0.875	256	2	0.680553752	-0.0025
cas-N6512_SVM	0.7673	2048	3	cas-N6512_SVM	0.7648	256	2	0.760057987	-0.0025
nr-er_SVM	0.7673	2048	3	nr-er_SVM	0.7648	256	2	0.877389322	-0.0025
cas-N6512_RFC	0.8766	1024	3	cas-N6512_RFC	0.8742	256	2	0.69365422	-0.0024
nr-er_FEST	0.767	1024	1	nr-er_FEST	0.7646	256	2	0.882292893	-0.0024
nr-ahr_SVM	0.8864	128	2	nr-ahr_SVM	0.884	256	3	0.839313413	-0.0024
sr-mmp_FEST	0.8776	1024	1	sr-mmp_FEST	0.8753	256	1	0.833321392	-0.0023
nr-er_FEST	0.7668	2048	1	nr-er_FEST	0.7646	256	2	0.892052179	-0.0022
cas-N6512_SVM	0.7546	128	2	cas-N6512_SVM	0.7527	256	3	0.82033731	-0.0019
nr-er_SVM	0.7546	128	2	nr-er_SVM	0.7527	256	3	0.907981159	-0.0019
nr-er_RFC	0.7664	1024	1	nr-er_RFC	0.7646	256	2	0.911611163	-0.0018
sr-mmp_FEST	0.8753	256	1	sr-mmp_FEST	0.8735	256	2	0.870059004	-0.0018
nr-er_FEST	0.767	1024	1	nr-er_FEST	0.7654	256	3	0.921331656	-0.0016
cas-N6512_RFC	0.8617	128	2	cas-N6512_RFC	0.8602	256	3	0.81557589	-0.0015
nr-er_FEST	0.7668	2048	1	nr-er_FEST	0.7654	256	3	0.931147455	-0.0014
cas-N6512_FEST	0.8852	1024	2	cas-N6512_FEST	0.8838	256	1	0.811413749	-0.0014
cas-N6512_FEST	0.8852	2048	2	cas-N6512_FEST	0.8838	256	1	0.811413749	-0.0014

sr-mmp_SVM	0.8806	1024	3	sr-mmp_SVM	0.8793	256	1	0.904191653	-0.0013
sr-mmp_FEST	0.8715	128	1	sr-mmp_FEST	0.8702	256	3	0.90701484	-0.0013
nr-ahr_SVM	0.8935	1024	3	nr-ahr_SVM	0.8923	256	2	0.916880762	-0.0012
cas-N6512_FEST	0.8618	128	2	cas-N6512_FEST	0.8607	256	3	0.86404887	-0.0011
nr-ahr_RFC	0.9014	256	1	nr-ahr_RFC	0.9005	256	2	0.935514438	-0.0009
nr-ahr_FEST	0.9029	1024	3	nr-ahr_FEST	0.9022	256	1	0.949472515	-0.0007
nr-ahr_RFC	0.9012	1024	3	nr-ahr_RFC	0.9005	256	2	0.949844631	-0.0007
nr-er_RFC	0.7664	1024	1	nr-er_RFC	0.7658	256	3	0.970461919	-0.0006
sr-mmp_RFC	0.8709	128	1	sr-mmp_RFC	0.8706	256	3	0.978502604	-0.0003
nr-ahr_RFC	0.9012	1024	3	nr-ahr_RFC	0.9014	256	1	0.985633361	0.0002
nr-ahr_RFC	0.8916	128	2	nr-ahr_RFC	0.8922	256	3	0.958545966	0.0006
nr-er_RFC	0.7639	2048	1	nr-er_RFC	0.7646	256	2	0.965618703	0.0007
nr-ahr_FEST	0.8907	128	2	nr-ahr_FEST	0.8915	256	3	0.944917468	0.0008
nr-er_FEST	0.7638	256	1	nr-er_FEST	0.7646	256	2	0.960713147	0.0008
nr-er_FEST	0.7646	256	2	nr-er_FEST	0.7654	256	3	0.960675336	0.0008
cas-N6512_FEST	0.8741	128	1	cas-N6512_FEST	0.875	256	2	0.88296177	0.0009
cas-N6512_SVM	0.7616	1024	1	cas-N6512_SVM	0.7626	256	1	0.903457408	0.001
cas-N6512_SVM	0.7616	2048	1	cas-N6512_SVM	0.7626	256	1	0.903457408	0.001
nr-er_SVM	0.7616	1024	1	nr-er_SVM	0.7626	256	1	0.951024754	0.001
nr-er_SVM	0.7616	2048	1	nr-er_SVM	0.7626	256	1	0.951024754	0.001
cas-N6512_RFC	0.8766	1024	3	cas-N6512_RFC	0.8776	256	1	0.868750252	0.001
nr-er_RFC	0.7646	256	2	nr-er_RFC	0.7658	256	3	0.941028508	0.0012
sr-mmp_SVM	0.8682	128	1	sr-mmp_SVM	0.8696	256	2	0.90050297	0.0014
nr-er_FEST	0.7638	256	1	nr-er_FEST	0.7654	256	3	0.921483558	0.0016
cas-N6512_SVM	0.7609	1024	3	cas-N6512_SVM	0.7626	256	1	0.836733181	0.0017
nr-er_SVM	0.7609	1024	3	nr-er_SVM	0.7626	256	1	0.916874632	0.0017
nr-er_RFC	0.7596	128	1	nr-er_RFC	0.7613	256	1	0.917000183	0.0017
nr-er_RFC	0.7639	2048	1	nr-er_RFC	0.7658	256	3	0.906795429	0.0019
sr-mmp_FEST	0.8715	128	1	sr-mmp_FEST	0.8735	256	2	0.856645778	0.002
nr-ahr_RFC	0.8983	128	1	nr-ahr_RFC	0.9005	256	2	0.844239679	0.0022
cas-N6512_SVM	0.7626	256	1	cas-N6512_SVM	0.7648	256	2	0.788995791	0.0022
nr-er_SVM	0.7626	256	1	nr-er_SVM	0.7648	256	2	0.892310331	0.0022
nr-ahr_RFC	0.8983	128	1	nr-ahr_RFC	0.9014	256	1	0.781487508	0.0031

cas-N6512_SVM	0.7616	1024	1	cas-N6512_SVM	0.7648	256	2	0.697342496	0.0032
cas-N6512_SVM	0.7616	2048	1	cas-N6512_SVM	0.7648	256	2	0.697342496	0.0032
nr-er_SVM	0.7616	1024	1	nr-er_SVM	0.7648	256	2	0.843982132	0.0032
nr-er_SVM	0.7616	2048	1	nr-er_SVM	0.7648	256	2	0.843982132	0.0032
nr-er_RFC	0.7613	256	1	nr-er_RFC	0.7646	256	2	0.839219104	0.0033
sr-mmp_FEST	0.8715	128	1	sr-mmp_FEST	0.8753	256	1	0.730674482	0.0038
nr-ahr_SVM	0.8885	128	1	nr-ahr_SVM	0.8923	256	2	0.743432541	0.0038
cas-N6512_FEST	0.8799	2048	3	cas-N6512_FEST	0.8838	256	1	0.511045022	0.0039
cas-N6512_SVM	0.7609	1024	3	cas-N6512_SVM	0.7648	256	2	0.63572174	0.0039
nr-er_SVM	0.7609	1024	3	nr-er_SVM	0.7648	256	2	0.810520453	0.0039
nr-ahr_FEST	0.895	128	1	nr-ahr_FEST	0.8993	256	2	0.703624556	0.0043
nr-er_RFC	0.7569	128	2	nr-er_RFC	0.7613	256	1	0.787688269	0.0044
sr-mmp_RFC	0.8709	128	1	sr-mmp_RFC	0.8754	256	2	0.683777931	0.0045
sr-mmp_FEST	0.8657	128	2	sr-mmp_FEST	0.8702	256	3	0.688635813	0.0045
nr-er_RFC	0.7613	256	1	nr-er_RFC	0.7658	256	3	0.781878601	0.0045
nr-er_RFC	0.7566	128	3	nr-er_RFC	0.7613	256	1	0.773631992	0.0047
nr-er_FEST	0.7589	128	2	nr-er_FEST	0.7638	256	1	0.763642206	0.0049
nr-er_RFC	0.7596	128	1	nr-er_RFC	0.7646	256	2	0.7587607	0.005
nr-ahr_SVM	0.8787	128	3	nr-ahr_SVM	0.884	256	3	0.658662581	0.0053
nr-ahr_SVM	0.8935	1024	3	nr-ahr_SVM	0.8989	256	1	0.634143118	0.0054
nr-er_FEST	0.7589	128	2	nr-er_FEST	0.7646	256	2	0.726366967	0.0057
nr-ahr_SVM	0.8864	128	2	nr-ahr_SVM	0.8923	256	2	0.612722878	0.0059
cas-N6512_SVM	0.7565	128	1	cas-N6512_SVM	0.7626	256	1	0.461369239	0.0061
sr-mmp_RFC	0.8645	128	2	sr-mmp_RFC	0.8706	256	3	0.587454131	0.0061
nr-er_SVM	0.7565	128	1	nr-er_SVM	0.7626	256	1	0.708727059	0.0061
nr-er_RFC	0.7596	128	1	nr-er_RFC	0.7658	256	3	0.703136908	0.0062
cas-N6512_RFC	0.8679	128	1	cas-N6512_RFC	0.8742	256	2	0.309340711	0.0063
cas-N6512_FEST	0.8775	1024	3	cas-N6512_FEST	0.8838	256	1	0.290815095	0.0063
nr-er_FEST	0.7589	128	2	nr-er_FEST	0.7654	256	3	0.68969333	0.0065
nr-er_FEST	0.7573	128	3	nr-er_FEST	0.7638	256	1	0.690243392	0.0065
nr-ahr_FEST	0.895	128	1	nr-ahr_FEST	0.9022	256	1	0.521591255	0.0072
nr-er_FEST	0.7573	128	3	nr-er_FEST	0.7646	256	2	0.654306971	0.0073
nr-er_FEST	0.7565	128	1	nr-er_FEST	0.7638	256	1	0.654607151	0.0073

sr-mmp_RFC	0.8709	128	1	sr-mmp_RFC	0.8784	256	1	0.495106881	0.0075	
nr-er_RFC	0.7569	128	2	nr-er_RFC	0.7646	256	2	0.636772726	0.0077	
sr-mmp_FEST	0.8657	128	2	sr-mmp_FEST	0.8735	256	2	0.485084767	0.0078	
cas-N6512_SVM	0.7546	128	2	cas-N6512_SVM	0.7626	256	1	0.334825461	0.008	
nr-er_RFC	0.7566	128	3	nr-er_RFC	0.7646	256	2	0.623758582	0.008	
nr-er_SVM	0.7546	128	2	nr-er_SVM	0.7626	256	1	0.624557218	0.008	
nr-er_FEST	0.7573	128	3	nr-er_FEST	0.7654	256	3	0.619120453	0.0081	
nr-er_FEST	0.7565	128	1	nr-er_FEST	0.7646	256	2	0.619447183	0.0081	
cas-N6512_SVM	0.7565	128	1	cas-N6512_SVM	0.7648	256	2	0.315277783	0.0083	
nr-er_SVM	0.7565	128	1	nr-er_SVM	0.7648	256	2	0.610782199	0.0083	
nr-ahr_FEST	0.8907	128	2	nr-ahr_FEST	0.8993	256	2	0.450666046	0.0086	
nr-ahr_RFC	0.8916	128	2	nr-ahr_RFC	0.9005	256	2	0.433069704	0.0089	
nr-er_RFC	0.7569	128	2	nr-er_RFC	0.7658	256	3	0.584928315	0.0089	
nr-er_FEST	0.7565	128	1	nr-er_FEST	0.7654	256	3	0.585103625	0.0089	
nr-er_RFC	0.7566	128	3	nr-er_RFC	0.7658	256	3	0.572403471	0.0092	
sr-mmp_FEST	0.8657	128	2	sr-mmp_FEST	0.8753	256	1	0.38884156	0.0096	
cas-N6512_FEST	0.8741	128	1	cas-N6512_FEST	0.8838	256	1	0.106296584	0.0097	
cas-N6512_RFC	0.8679	128	1	cas-N6512_RFC	0.8776	256	1	0.115121749	0.0097	
nr-ahr_RFC	0.8825	128	3	nr-ahr_RFC	0.8922	256	3	0.408743421	0.0097	
nr-ahr_RFC	0.8916	128	2	nr-ahr_RFC	0.9014	256	1	0.387127249	0.0098	
cas-N6512_SVM	0.7546	128	2	cas-N6512_SVM	0.7648	256	2	0.21792811	0.0102	
nr-er_SVM	0.7546	128	2	nr-er_SVM	0.7648	256	2	0.532103346	0.0102	
nr-ahr_SVM	0.8885	128	1	nr-ahr_SVM	0.8989	256	1	0.364134306	0.0104	
sr-mmp_SVM	0.855	128	2	sr-mmp_SVM	0.8655	256	3	0.360680578	0.0105	
sr-mmp_RFC	0.8645	128	2	sr-mmp_RFC	0.8754	256	2	0.328686492	0.0109	
sr-mmp_SVM	0.8682	128	1	sr-mmp_SVM	0.8793	256	1	0.314027203	0.0111	
nr-ahr_FEST	0.8907	128	2	nr-ahr_FEST	0.9022	256	1	0.31023905	0.0115	
cas-N6512_SVM	0.7407	128	3	cas-N6512_SVM	0.7527	256	3	0.156151175	0.012	
nr-er_SVM	0.7407	128	3	nr-er_SVM	0.7527	256	3	0.468601115	0.012	
nr-ahr_FEST	0.8791	128	3	nr-ahr_FEST	0.8915	256	3	0.294505981	0.0124	
nr-ahr_SVM	0.8864	128	2	nr-ahr_SVM	0.8989	256	1	0.277322921	0.0125	
nr-ahr_SVM	0.8787	128	3	nr-ahr_SVM	0.8923	256	2	0.2498862	0.0136	
sr-mmp_RFC	0.8645	128	2	sr-mmp_RFC	0.8784	256	1	0.210704932	0.0139	

sr-mmp_FEST	0.8558	128	3	sr-mmp_FEST	0.8702	256	3	0.206326676	0.0144
sr-mmp_SVM	0.855	128	2	sr-mmp_SVM	0.8696	256	2	0.201008877	0.0146
sr-mmp_SVM	0.8506	128	3	sr-mmp_SVM	0.8655	256	3	0.197299304	0.0149
sr-mmp_RFC	0.8552	128	3	sr-mmp_RFC	0.8706	256	3	0.176647415	0.0154
sr-mmp_FEST	0.8558	128	3	sr-mmp_FEST	0.8735	256	2	0.118492658	0.0177
nr-ahr_RFC	0.8825	128	3	nr-ahr_RFC	0.9005	256	2	0.119276887	0.018
nr-ahr_RFC	0.8825	128	3	nr-ahr_RFC	0.9014	256	1	0.101284618	0.0189
sr-mmp_SVM	0.8506	128	3	sr-mmp_SVM	0.8696	256	2	0.098152004	0.019
sr-mmp_FEST	0.8558	128	3	sr-mmp_FEST	0.8753	256	1	0.084586365	0.0195
nr-ahr_SVM	0.8787	128	3	nr-ahr_SVM	0.8989	256	1	0.083528435	0.0202
nr-ahr_FEST	0.8791	128	3	nr-ahr_FEST	0.8993	256	2	0.083064324	0.0202
sr-mmp_RFC	0.8552	128	3	sr-mmp_RFC	0.8754	256	2	0.074210058	0.0202
nr-er_SVM	0.7407	128	3	nr-er_SVM	0.7626	256	1	0.183524213	0.0219
nr-er_SVM	0.7407	128	3	nr-er_SVM	0.7648	256	2	0.142794596	0.0241
nr-er_SVM	0.7694	1024	2	nr-er_SVM	0.7407	128	3	0.080122152	-0.0287
nr-er_FEST	0.779	1024	2	nr-er_FEST	0.7565	128	1	0.163894199	-0.0225
sr-mmp_FEST	0.8776	1024	1	sr-mmp_FEST	0.8558	128	3	0.05298495	-0.0218
nr-er_FEST	0.779	1024	2	nr-er_FEST	0.7573	128	3	0.179201751	-0.0217
nr-er_SVM	0.7616	1024	1	nr-er_SVM	0.7407	128	3	0.204611494	-0.0209
nr-er_RFC	0.777	1024	2	nr-er_RFC	0.7566	128	3	0.207461406	-0.0204
nr-er_SVM	0.7609	1024	3	nr-er_SVM	0.7407	128	3	0.220369767	-0.0202
nr-er_FEST	0.779	1024	2	nr-er_FEST	0.7589	128	2	0.213007275	-0.0201
nr-er_RFC	0.777	1024	2	nr-er_RFC	0.7569	128	2	0.214143182	-0.0201
sr-mmp_RFC	0.8841	1024	2	sr-mmp_RFC	0.8645	128	2	0.074806683	-0.0196
sr-mmp_RFC	0.8841	1024	3	sr-mmp_RFC	0.8645	128	2	0.074806683	-0.0196
nr-ahr_RFC	0.9012	1024	3	nr-ahr_RFC	0.8825	128	3	0.105089235	-0.0187
nr-ahr_SVM	0.905	1024	1	nr-ahr_SVM	0.8864	128	2	0.101668388	-0.0186
sr-mmp_RFC	0.8826	1024	1	sr-mmp_RFC	0.8645	128	2	0.10076583	-0.0181
nr-ahr_FEST	0.9086	1024	2	nr-ahr_FEST	0.8907	128	2	0.109331024	-0.0179
sr-mmp_SVM	0.8682	128	1	sr-mmp_SVM	0.8506	128	3	0.126295832	-0.0176
sr-mmp_SVM	0.8857	1024	1	sr-mmp_SVM	0.8682	128	1	0.10858291	-0.0175
nr-er_RFC	0.777	1024	2	nr-er_RFC	0.7596	128	1	0.281447079	-0.0174
sr-mmp_FEST	0.8828	1024	2	sr-mmp_FEST	0.8657	128	2	0.120199395	-0.0171

nr-ahr_SVM	0.9031	1024	2	nr-ahr_SVM	0.8864	128	2	0.143243649	-0.0167
nr-ahr_SVM	0.905	1024	1	nr-ahr_SVM	0.8885	128	1	0.144823045	-0.0165
nr-ahr_RFC	0.9076	1024	2	nr-ahr_RFC	0.8916	128	2	0.152495279	-0.016
nr-ahr_FEST	0.895	128	1	nr-ahr_FEST	0.8791	128	3	0.176056076	-0.0159
nr-ahr_RFC	0.9074	1024	1	nr-ahr_RFC	0.8916	128	2	0.157869894	-0.0158
nr-ahr_RFC	0.8983	128	1	nr-ahr_RFC	0.8825	128	3	0.17336417	-0.0158
nr-er_FEST	0.7723	1024	3	nr-er_FEST	0.7565	128	1	0.330389485	-0.0158
nr-er_SVM	0.7565	128	1	nr-er_SVM	0.7407	128	3	0.338954202	-0.0158
cas-N6512_SVM	0.7565	128	1	cas-N6512_SVM	0.7407	128	3	0.061053825	-0.0158
sr-mmp_FEST	0.8715	128	1	sr-mmp_FEST	0.8558	128	3	0.167426656	-0.0157
sr-mmp_RFC	0.8709	128	1	sr-mmp_RFC	0.8552	128	3	0.168174736	-0.0157
nr-er_RFC	0.7722	1024	3	nr-er_RFC	0.7566	128	3	0.336550546	-0.0156
nr-er_RFC	0.7722	1024	3	nr-er_RFC	0.7569	128	2	0.345842536	-0.0153
sr-mmp_SVM	0.8833	1024	2	sr-mmp_SVM	0.8682	128	1	0.167941502	-0.0151
nr-er_FEST	0.7723	1024	3	nr-er_FEST	0.7573	128	3	0.355241465	-0.015
sr-mmp_FEST	0.8806	1024	3	sr-mmp_FEST	0.8657	128	2	0.177320459	-0.0149
nr-ahr_SVM	0.8935	1024	3	nr-ahr_SVM	0.8787	128	3	0.209496072	-0.0148
nr-er_SVM	0.7694	1024	2	nr-er_SVM	0.7546	128	2	0.363296799	-0.0148
cas-N6512_SVM	0.7694	1024	2	cas-N6512_SVM	0.7546	128	2	0.072640238	-0.0148
nr-ahr_SVM	0.9031	1024	2	nr-ahr_SVM	0.8885	128	1	0.198781132	-0.0146
nr-ahr_FEST	0.9052	1024	1	nr-ahr_FEST	0.8907	128	2	0.197899017	-0.0145
nr-er_SVM	0.7546	128	2	nr-er_SVM	0.7407	128	3	0.400701052	-0.0139
cas-N6512_SVM	0.7546	128	2	cas-N6512_SVM	0.7407	128	3	0.099917562	-0.0139
nr-ahr_FEST	0.9086	1024	2	nr-ahr_FEST	0.895	128	1	0.219609288	-0.0136
nr-er_FEST	0.7723	1024	3	nr-er_FEST	0.7589	128	2	0.408450142	-0.0134
sr-mmp_RFC	0.8841	1024	2	sr-mmp_RFC	0.8709	128	1	0.225386659	-0.0132
sr-mmp_RFC	0.8841	1024	3	sr-mmp_RFC	0.8709	128	1	0.225386659	-0.0132
sr-mmp_SVM	0.8682	128	1	sr-mmp_SVM	0.855	128	2	0.248635986	-0.0132
cas-N6512_SVM	0.7694	1024	2	cas-N6512_SVM	0.7565	128	1	0.11705879	-0.0129
nr-er_SVM	0.7694	1024	2	nr-er_SVM	0.7565	128	1	0.42763664	-0.0129
nr-er_RFC	0.7722	1024	3	nr-er_RFC	0.7596	128	1	0.436839348	-0.0126
sr-mmp_SVM	0.8806	1024	3	sr-mmp_SVM	0.8682	128	1	0.259676266	-0.0124
nr-ahr_FEST	0.9029	1024	3	nr-ahr_FEST	0.8907	128	2	0.281004987	-0.0122

sr-mmp_FEST	0.8776	1024	1	sr-mmp_FEST	0.8657	128	2	0.28365701	-0.0119
sr-mmp_RFC	0.8826	1024	1	sr-mmp_RFC	0.8709	128	1	0.283810128	-0.0117
nr-ahr_FEST	0.8907	128	2	nr-ahr_FEST	0.8791	128	3	0.327474363	-0.0116
sr-mmp_FEST	0.8828	1024	2	sr-mmp_FEST	0.8715	128	1	0.299953874	-0.0113
cas-N6512_FEST	0.8852	1024	2	cas-N6512_FEST	0.8741	128	1	0.063807797	-0.0111
nr-er_FEST	0.767	1024	1	nr-er_FEST	0.7565	128	1	0.51911232	-0.0105
nr-ahr_FEST	0.9052	1024	1	nr-ahr_FEST	0.895	128	1	0.360819139	-0.0102
sr-mmp_FEST	0.8657	128	2	sr-mmp_FEST	0.8558	128	3	0.388108448	-0.0099
nr-ahr_SVM	0.8885	128	1	nr-ahr_SVM	0.8787	128	3	0.410288067	-0.0098
nr-er_RFC	0.7664	1024	1	nr-er_RFC	0.7566	128	3	0.547474383	-0.0098
nr-er_FEST	0.767	1024	1	nr-er_FEST	0.7573	128	3	0.551265784	-0.0097
nr-ahr_RFC	0.9012	1024	3	nr-ahr_RFC	0.8916	128	2	0.397086185	-0.0096
nr-er_RFC	0.7664	1024	1	nr-er_RFC	0.7569	128	2	0.559736047	-0.0095
sr-mmp_RFC	0.8645	128	2	sr-mmp_RFC	0.8552	128	3	0.418711528	-0.0093
nr-ahr_RFC	0.9076	1024	2	nr-ahr_RFC	0.8983	128	1	0.39893059	-0.0093
sr-mmp_FEST	0.8806	1024	3	sr-mmp_FEST	0.8715	128	1	0.405628403	-0.0091
nr-ahr_RFC	0.9074	1024	1	nr-ahr_RFC	0.8983	128	1	0.409359246	-0.0091
nr-ahr_RFC	0.8916	128	2	nr-ahr_RFC	0.8825	128	3	0.43884981	-0.0091
cas-N6512_RFC	0.8766	1024	3	cas-N6512_RFC	0.8679	128	1	0.158419971	-0.0087
nr-er_FEST	0.767	1024	1	nr-er_FEST	0.7589	128	2	0.618459331	-0.0081
nr-ahr_FEST	0.9029	1024	3	nr-ahr_FEST	0.895	128	1	0.481275456	-0.0079
nr-ahr_SVM	0.8864	128	2	nr-ahr_SVM	0.8787	128	3	0.519238113	-0.0077
nr-ahr_SVM	0.8935	1024	3	nr-ahr_SVM	0.8864	128	2	0.541500648	-0.0071
cas-N6512_SVM	0.7616	1024	1	cas-N6512_SVM	0.7546	128	2	0.399147849	-0.007
nr-er_SVM	0.7616	1024	1	nr-er_SVM	0.7546	128	2	0.668667937	-0.007
nr-er_RFC	0.7664	1024	1	nr-er_RFC	0.7596	128	1	0.675858846	-0.0068
nr-ahr_RFC	0.8983	128	1	nr-ahr_RFC	0.8916	128	2	0.556862462	-0.0067
sr-mmp_RFC	0.8709	128	1	sr-mmp_RFC	0.8645	128	2	0.569034121	-0.0064
cas-N6512_SVM	0.7609	1024	3	cas-N6512_SVM	0.7546	128	2	0.448232338	-0.0063
nr-er_SVM	0.7609	1024	3	nr-er_SVM	0.7546	128	2	0.700220411	-0.0063
cas-N6512_RFC	0.8679	128	1	cas-N6512_RFC	0.8617	128	2	0.328357352	-0.0062
sr-mmp_FEST	0.8776	1024	1	sr-mmp_FEST	0.8715	128	1	0.579122781	-0.0061
sr-mmp_FEST	0.8715	128	1	sr-mmp_FEST	0.8657	128	2	0.604800551	-0.0058

cas-N6512_SVM	0.7616	1024	1	cas-N6512_SVM	0.7565	128	1	0.538357304	-0.0051	
nr-er_SVM	0.7616	1024	1	nr-er_SVM	0.7565	128	1	0.754931668	-0.0051	
nr-ahr_SVM	0.8935	1024	3	nr-ahr_SVM	0.8885	128	1	0.665980566	-0.005	
cas-N6512_SVM	0.7609	1024	3	cas-N6512_SVM	0.7565	128	1	0.595751168	-0.0044	
nr-er_SVM	0.7609	1024	3	nr-er_SVM	0.7565	128	1	0.787783115	-0.0044	
sr-mmp_SVM	0.855	128	2	sr-mmp_SVM	0.8506	128	3	0.707490817	-0.0044	
nr-ahr_FEST	0.895	128	1	nr-ahr_FEST	0.8907	128	2	0.70847202	-0.0043	
cas-N6512_FEST	0.8775	1024	3	cas-N6512_FEST	0.8741	128	1	0.576218435	-0.0034	
nr-er_RFC	0.7596	128	1	nr-er_RFC	0.7566	128	3	0.854475145	-0.003	
nr-ahr_RFC	0.9012	1024	3	nr-ahr_RFC	0.8983	128	1	0.795346288	-0.0029	
nr-er_RFC	0.7596	128	1	nr-er_RFC	0.7569	128	2	0.868866705	-0.0027	
nr-ahr_SVM	0.8885	128	1	nr-ahr_SVM	0.8864	128	2	0.8580264	-0.0021	
cas-N6512_SVM	0.7565	128	1	cas-N6512_SVM	0.7546	128	2	0.819766793	-0.0019	
nr-er_SVM	0.7565	128	1	nr-er_SVM	0.7546	128	2	0.907789259	-0.0019	
nr-er_FEST	0.7589	128	2	nr-er_FEST	0.7573	128	3	0.922076321	-0.0016	
nr-er_RFC	0.7569	128	2	nr-er_RFC	0.7566	128	3	0.985389216	-0.0003	
nr-er_FEST	0.7565	128	1	nr-er_FEST	0.7573	128	3	0.961044561	0.0008	
nr-er_FEST	0.7565	128	1	nr-er_FEST	0.7589	128	2	0.883398762	0.0024	

infile_a = infile a dataset_method; auc_a = ROC area file a; fptype_a = fimgerprint type file a; radius_a = fingerprint radius file a;
infile_b = infile b dataset_method; auc_b = ROC area file b; fptype_b = fimgerprint type file b; radius_b = fingerprint radius file b;
P: non-directional (two-tailed) = p-value; difference auc_b-auc_a; stat significant = statistical significance at $p < 0.5$
